# Diels–Alder reaction of β-fluoro-β-nitrostyrenes with cyclic dienes

**DOI:** 10.3762/bjoc.17.27

**Published:** 2021-01-27

**Authors:** Savva A Ponomarev, Roman V Larkovich, Alexander S Aldoshin, Andrey A Tabolin, Sema L Ioffe, Jonathan Groß, Till Opatz, Valentine G Nenajdenko

**Affiliations:** 1 Department of Chemistry, Lomonosov Moscow State University, Leninskie gory 1, Moscow, 119991, Russian Federation; 2 N. D. Zelinsky Institute of Organic Chemistry, Russian Academy of Sciences Leninsky prosp. 47, Moscow 119991, Russian Federation; 3 Department of Chemistry Johannes Gutenberg-University, Duesbergweg 10–14, D-55128 Mainz, Germany

**Keywords:** Diels–Alder reaction, fluorine, nitrostyrene, norbornene, stereochemistry

## Abstract

The Diels–Alder reaction of β-fluoro-β-nitrostyrenes with cyclic 1,3-dienes was investigated. A series of novel monofluorinated norbornenes was prepared in up to 97% yield. The reaction with 1,3-cyclohexadiene permits the preparation of monofluorinated bicyclo[2.2.2]oct-2-enes. The kinetic data of the reactions with 1,3-cyclopentadiene and 1,3-cyclohexadiene were used to calculate activation parameters. Furthermore, the synthetic utility of the cycloadducts obtained was demonstrated.

## Introduction

Organofluorine compounds play an exceptionally important role in various fields of science and technology. The incorporation of fluorine into molecules can significantly influence their pharmacokinetic and physicochemical properties and enhance their metabolic and chemical stability [1–5]. For instance, nearly a quarter of the currently manufactured agrochemical and pharmaceutical products contains at least one fluorine atom [6–8]. Fluorinated functional materials have also found wide application as durable ion exchange membranes, e.g., in fuel cells [9–11], as thermoplastic polymers [12–14], in electronic and optoelectronic technologies [15], and in liquid crystal display applications [16–21], etc [22]. The use of fluorinated building blocks is a very convenient approach and in many cases represents an indispensable alternative to late-stage fluorinations in the preparation of such unique materials [23].

The Diels–Alder reaction is considered a versatile and powerful tool for assembling a variety of fluorinated carbo- and heterocycles using either the diene [24–30] or the dienophile component [31–39] as fluorine-containing building blocks. The application of [4 + 2] cycloadditions for the preparation of fluorinated bicyclic compounds has attracted much attention [40–47]. In this regard, the development of new protocols to relevant monofluorinated bicyclic molecules involving novel versatile fluorine-containing building blocks is of key importance. Fluoroalkenes are recognized to be one of the most widely used fluorine-containing building blocks [48–49]. Recently, we have developed an efficient stereoselective synthesis of β-fluoro-β-nitrostyrenes **1** based on the radical nitration of 2-bromo-2-fluorostyrenes [50]. This process takes place with simultaneous elimination of bromine, and gives the target structures solely in the Z-isomeric form in high yields (up to 92%). These fluorine-containing olefins activated by a nitro group proved to be the appealing building blocks for the construction of numerous monofluorinated compounds [51–56]. This paper is devoted to a new synthetic approach to novel monofluorinated bicyclic compounds, namely norbornenes and bicyclo[2.2.2]oct-2-enes and their subsequent functionalization. The present study is our follow-up work on the Diels–Alder reaction involving β-fluoro-β-nitrostyrenes [57].

A recent review reported that by 2018, the total number of publications and patents related to the production and use of norbornene and norbornadiene derivatives had exceeded 30,000 [58]. Indeed, norbornene and its derivatives have found application in medicine, agriculture, microelectronics, and rocket technology as well as in production of polymeric materials, efficient gas separation membranes and solar energy converters [58]. Considering the high interest in such structures and the unique role of fluorine, we believe that novel norbornene derivatives obtained in the framework of this study can become relevant compounds in practical use.

## Results and Discussion

**Scheme 1:**
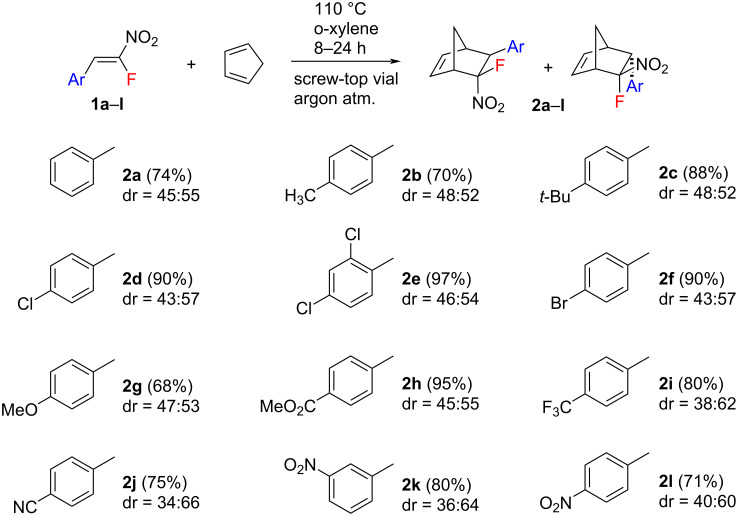
Scope of the nitrostyrenes **1** in the Diels–Alder reaction with CPD (dr = *exo*:*endo*).

**Figure 1:**
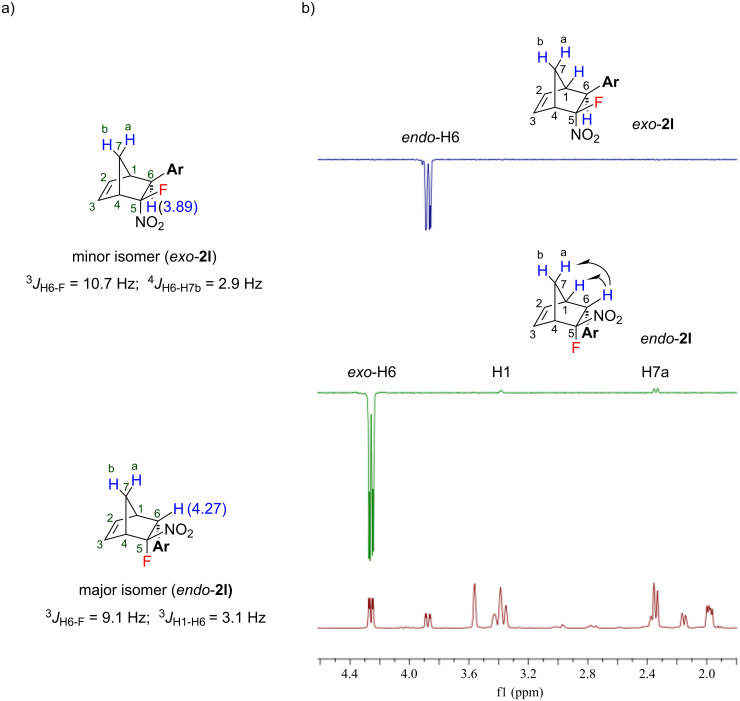
The structure assignment of norbornenes **2** by ^1^H (a) and NOE (b) NMR spectroscopy.

Initially, we studied the Diels–Alder reaction of β-fluoro-β-nitrostyrenes **1** with 1,3-cyclopentadiene (CPD) to prepare a series of novel monofluorinated norbornenes. The starting nitrostyrenes were prepared and used in the *Z*-isomer form. The transformations were conducted in screw-top vials in *o*-xylene at 110 °C using a fivefold excess of the diene (Scheme 1). The reaction proceeded smoothly under these conditions to give the target cycloadducts **2** as a mixture of *exo* and *endo*-isomers in high isolated yield (up to 97%). It should be noted that in the present work we indicate an isomer as e*xo* or *endo* according to the stereo-position of the fluorine atom. Thus *exo*-**2** and *endo*-**2** means 5-*exo*-fluoro-5-*endo*-nitro-6-*exo*-arylnorbornene and 5-*endo*-fluoro-5-*exo*-nitro-6-*endo*-arylnorbornene, respectively (Figure 1). Both diastereomers are formed in a nearly 1:1 ratio for the majority of the substituents on the aryl group of the nitrostyrenes **1**. However, a higher diastereoselectivity towards the *endo*-isomer was observed when strong electron-withdrawing groups (EWGs) were present in the dienophile. For example, in the case of the 4-cyano and the 3-nitro-substituted derivative, the ratio of *endo*/*exo* was 2:1.

The stereochemistry of the products **2a–l** can be unambiguously assigned using ^1^H NMR spectroscopy. According to the literature data [59] the dienophile-derived proton at C6 resonates at lower field in the *exo*-form than the corresponding proton of the *endo*-isomer. For example, in the case of **2l**, the ^1^H NMR spectrum shows a doublet of doublet signal for H6 at 3.89 ppm for the minor isomer and at 4.27 ppm for the major isomer (Figure 1a). A significant chemical shift difference is observed for the aryl proton signals of the *exo*- and *endo*-isomeric norbornenes **2**. Most probably such significant difference in the chemical shifts can be explained by the double bond anisotropy of the norbornene molecule [60]. The stereochemical assignments are in full accordance with the values of vicinal (^3^*J*_H6-F_ and ^3^*J*_H1-H6_) and long-range coupling constants (^4^*J*_H6-H7b_). According to the literature data, the value of ^3^*J*_H1-H6_ is larger than that of ^4^*J*_H6-H7b_ [32,61–63]. For example, the ^1^H NMR spectrum of the minor isomer of **2l** showed the coupling constants ^3^*J*_H6-F_ = 10.7 Hz and ^4^*J*_H6-H7b_ = 2.9 Hz consistent with an *exo*-geometry. In contrast, the major isomer having constants ^3^*J*_H6-F_ = 9.1 Hz and ^3^*J*_H1-H6_ = 3.1 Hz was ascribed to the *endo*-form (Figure 1). It should be noted that this observation applies for all cases investigated. The value of the coupling constant ^3^*J*_H6-F_ between the *exo*-F and *endo*-H6 was always larger than the corresponding value between the *endo*-F and *exo*-H6. The stereochemical assignments were additionally confirmed by nuclear Overhauser effect spectroscopy (NOE). The peak of H6 was selected to be selectively excited for each isomer. As expected, in the case of *endo*-**2l** the NOE peaks resulted from the interaction of *exo*-H6 with H1 and H7a were observed. Whereas for *exo*-**2l** due to the opposite side position of *endo*-H6 there was no interaction observed. Thus, using these spectral data all the pairs of *exo*- and *endo*-isomers **2** obtained can be assigned unambiguously.

**Figure 2:**
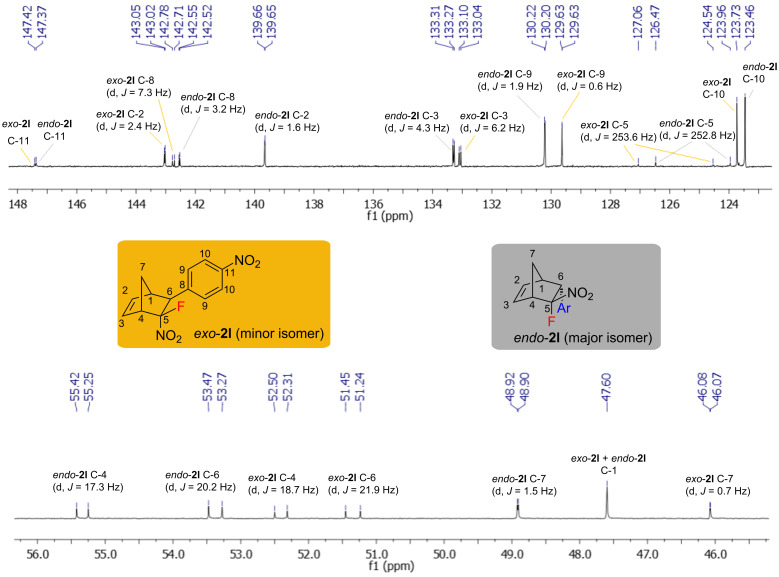
^13^C NMR spectrum of the mixture of *exo*- and *endo*-isomers of norbornene **2l**.

Moreover, the ^13^C NMR spectra of the *exo* and *endo*-isomers exhibit a significant difference (approximately 3 ppm) in the chemical shifts for some carbon atoms (Figure 2). A considerable difference in the chemical shifts was observed for C-7 of the methylene bridge (46.1 for the *exo-* vs 48.9 ppm for the *endo*-isomer), C-6 (51.3 for the *exo* vs 53.4 for the *endo-*isomer), C-4 (52.4 for the *exo*- vs 55.3 ppm for the *endo*-isomer), and C-2 (139.7 for the *endo*- vs 143.0 ppm for the *exo*-isomer). The same pattern in the chemical shifts and coupling constants was observed for all structures **2** synthesized.

For further insights into the mechanistic background of the *endo-exo* selectivity, the Diels–Alder reaction of CPD with the model nitrostyrene **1h** was simulated in silico to predict the reaction pathway, the reaction rate constants, and the activation enthalpies. Density functional theory calculations were conducted for the reactants, products, and transition states using the B3LYP [64–66] and M062X [67] level of theory in combination with a Pople basis set and the IEFPCM [68] solvation model for *o*-xylene. Both functionals are already known in the literature for the investigation of cycloadditions [69–72]. For the computational details the reader is referred to Supporting Information File 1.

**Figure 3:**
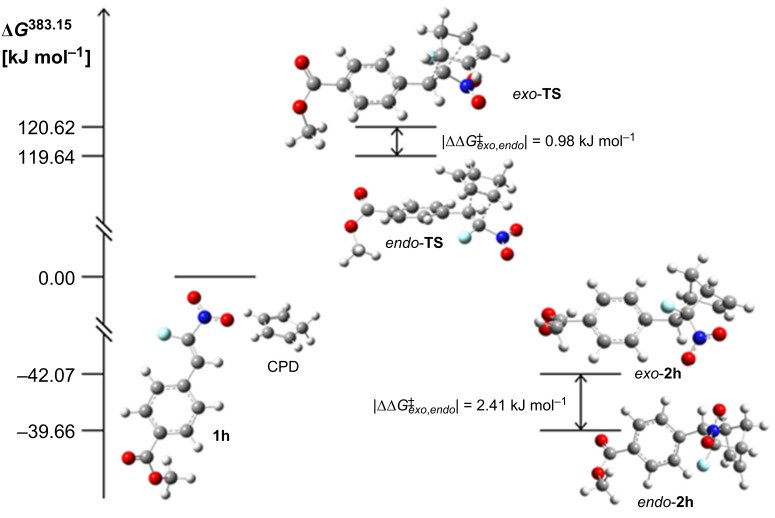
The predicted reaction pathway for the Diels–Alder reaction of nitrostyrene **1h** with CPD is displayed. The path leading to the *endo-***2h** isomer has a lower lying transition state and is more exergonic than the path resulting in the *exo-***2h** isomer. Energies are given in kJ mol^−1^ and were calculated with M062X/6-311+G(d,p) at 383.15 K in *o*-xylene (the corresponding B3LYP reaction pathway is not shown).

The predicted reaction pathways for the formation of the *exo*- and *endo*-isomeric norbornene **2h** using M062X are displayed in Figure 3. For each isomer one transition state *exo*-**TS** and *endo*-**TS** was identified. The former transition state is higher in energy and leads to the less exergonic product *exo*-**2h**. The *exo* and *endo*-isomers were predicted to have free energies of activation (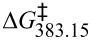) of 120.62 and 119.64 kJ mol^−1^, respectively. The corresponding predicted reaction free energies (Δ*G*_383.15_) are −39.66 and −42.07 kJ mol^−1^. With the former values of Δ*G*^‡^, the reaction rate coefficient *k* can be calculated using the Eyring equation (Equation 1) [73–74]:

[1] $$k(T)=\frac{k_{\text{B}}T}{hc_{0}}e^{-\Delta G_{383.15\text{K}}^{‡}/RT}$$

For *T* = 110 °C, the predicted ratio of *k*_endo_/*k*_exo_ = 1.36 (1.68 for B3LYP) is in good accordance with the experimentally observed diastereomeric ratio of 1.22. The larger discrepancy in case of the B3LYP functional may be due to the fact that dispersion effects are not included, whereas M062X includes nonlocal effects of electronic dispersion [70,75].

**Scheme 2:**
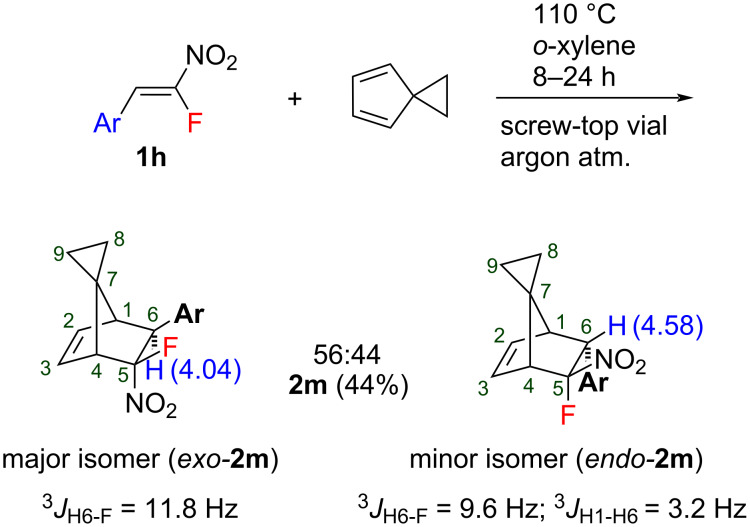
The Diels–Alder reaction of nitrostyrene **1h** with spiro[2.4]hepta-4,6-diene.

We also demonstrate the preparation of norbornene structures substituted at the methylene bridge. The reaction of model nitrostyrene **1h** with spiro[2.4]hepta-4,6-diene was carried out (Scheme 2). As a result, the corresponding norbornene **2m** having a cyclopropane ring was obtained in moderate yield (44%). The cycloaddition proceeds much more slowly as a result of the high steric demand of the cyclopropyl ring of the spirodiene compared to the CH_2_ group of cyclopentadiene. We believe that this is the reason of the lower yield in comparison to the reaction with CPD. The stereochemical assignment was performed using ^1^H NMR spectroscopy (Scheme 2) to show similar peculiarities of the spectra. In contrast to the reaction with CPD, a slight prevalence in the formation of the *exo*-isomer (*exo*:*endo* = 56:44) was observed for the product **2m**.

**Scheme 3:**
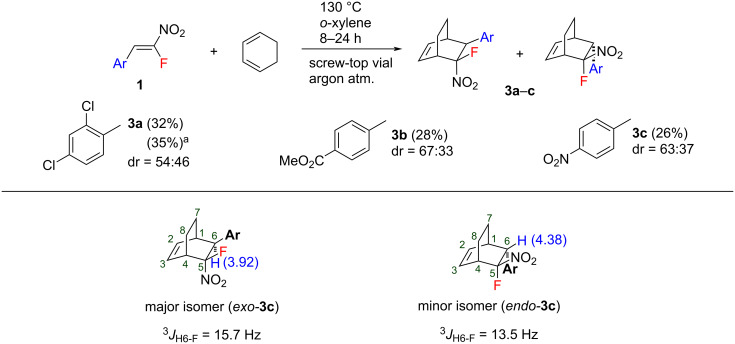
Diels–Alder reaction of nitrostyrenes **1** with CHD (dr = *exo*:*endo*). (а) Reaction under microwave activation.

Next, the reaction with 1,3-cyclohexadiene (CHD) was investigated. It was found that the reaction is very sensitive to the structure of starting diene and in the case of CHD proceeds much more slowly. Both thermal and microwave (MW) activation (Scheme 3) was investigated to accelerate the reaction with CHD. However, in all cases, the yields of the target cycloadducts **3** were below 35% despite the full conversion of the nitrostyrenes **1** which is common for this type of dienophiles (Scheme 3). The stereochemical assignment was made similarly to the norbornene structures using ^1^H NMR spectroscopy (Scheme 3). Larger values of ^3^*J*_H6-F_ were observed for the *exo*-F isomers. The presence of a strong EWG on the aryl substituent led to higher stereoselectivity. For example, approximately a 2:1 ratio was observed for the nitro- and carboxymethyl-substituted products **3b**, **3c**, whereas in the absence of a strong EWG, the ratio was about 1:1 (**3a**). However, in contrast to CPD derivatives, the major products formed in the reaction with CHD have *exo*-configuration.

**Scheme 4:**
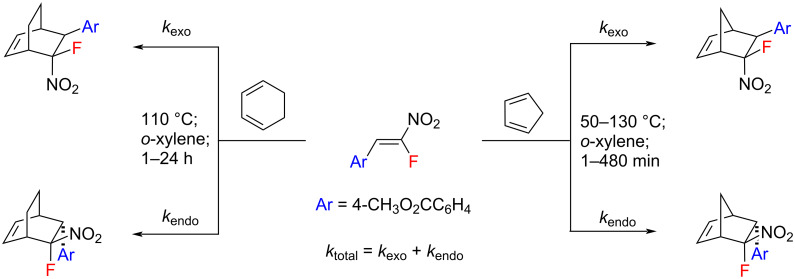
Kinetic study of reactions of **1h** with CPD and CHD.

To gain deeper insights into the reaction, we carried out some kinetic studies to evaluate and compare the reactivities of CHD and CPD in the reactions with model nitrostyrene **1h** (Scheme 4). All the kinetic runs were performed using a ≈43–49 molar excess of the diene in *o*-xylene (1:1) to provide pseudo-first order conditions. Conversions (*F*) of **1** were measured by ^1^H NMR spectroscopy. The reactions were found to proceed under the kinetic control since the isomer ratio remained constant throughout the reaction course regardless of the temperature (Table 1).

**Table 1:** Kinetic parameters for the reactions of **1h** with CPD and CHD.

entry	diene	*T*, °C	molar ratio *exo*/*endo*	k*·10^4^ s^−1^	*k*_total_·10^5^ L/mol·s	*k*_exo_·10^5^_,_ L/mol·s	*k*_endo_·10^5^*_,_* L/mol·s	R_corr_

1	CPD	50	46:54	1.00	1.67	0.090	0.077	0.997
2	CPD	80	46:54	5.78	9.72	5.26	4.46	0.998
3	CPD	110	46:54	35.62	59.91	32.39	27.53	0.990
4	CPD	130	46:54	46.20	77.71	41.99	35.72	0.999
5	CHD	110	61:39	0.12	0.224	0.137	0.087	0.999

**Figure 4:**
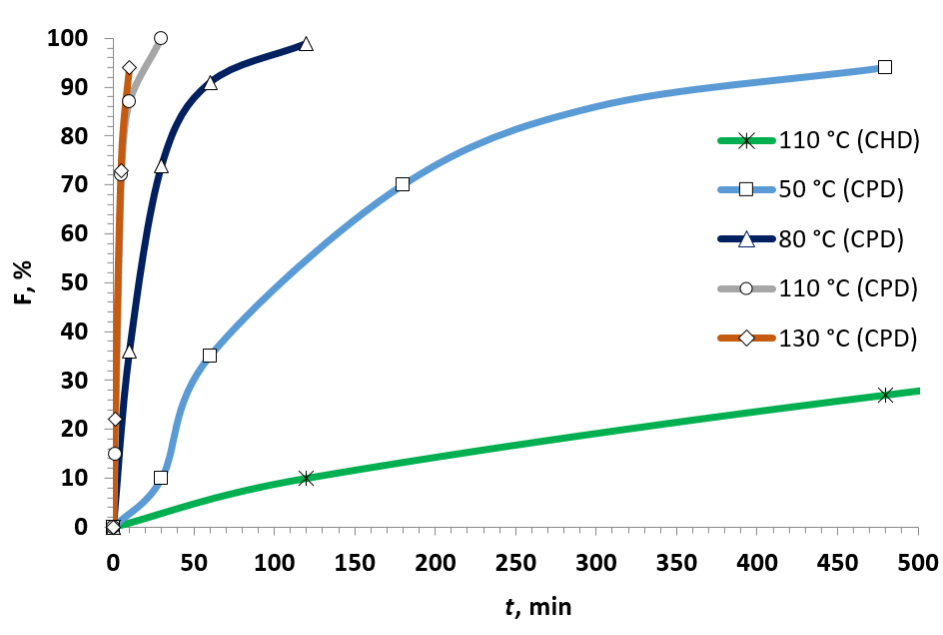
Kinetic curves for the reactions of nitrostyrene **1h** with CPD (50–130 °C) and CHD at 110 °C.

The total effective pseudo-first order rate constants k* were obtained by plotting the experimental values of ln(*c*_0_/*c*) versus time with good correlations (Table 1). The overall second-order rate total constants *k*_total_ were calculated from the effective k* and initial concentration of the diene (Table 1). The individual constants for the *endo* and *exo*-isomers (*k*_endo_ and *k*_exo_) were evaluated by multiplication of *k*_total_ with the molar fractions of the isomers (Table 1). The data obtained demonstrated that the overall reaction rate for CHD is 267 times lower than that for CPD at 110 °C (Figure 4, Table 1). Such a large difference in the reactivity of CHD and CPD was described in the literature. For example, in model reactions with tetracyanoethene, the difference was 2600-fold at 20 °C [76]. The activation parameters were estimated for the reaction of **1h** with CPD by plotting ln(*k*/*T*) versus 1/*T* according to the Eyring equation (Equations 2–4) [77].

[2]
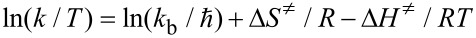


[3] $$\ln(k_{endo}/T)=1.89-6208/T\text{ (}R_{\text{corr}}=0.989)$$

[4] $$\ln(k_{exo}/T)=1.72-6207/T\text{ (}R_{\text{corr}}=0.989)$$

The activation enthalpies (Δ*H*^≠^) for *exo-* and *endo-***1h** were found to be identical for both reaction pathways (51.6 kJ mol^−1^). Whereas the entropies of activation (Δ*S*^≠^) were −181.8 and −183.1 J mol^−1^ K^−1^ for the formation of the *endo* and *exo*-isomers, respectively. The values obtained are typical for concerted [4 + 2]-cycloaddition reactions [60]. The free energies of activation (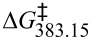) were calculated for 121.26 kJ mol^−1^ for *endo***-1h** and 121.75 kJ mol^−1^ for *exo***-1h** and were consistent with the predicted ones.

**Scheme 5:**
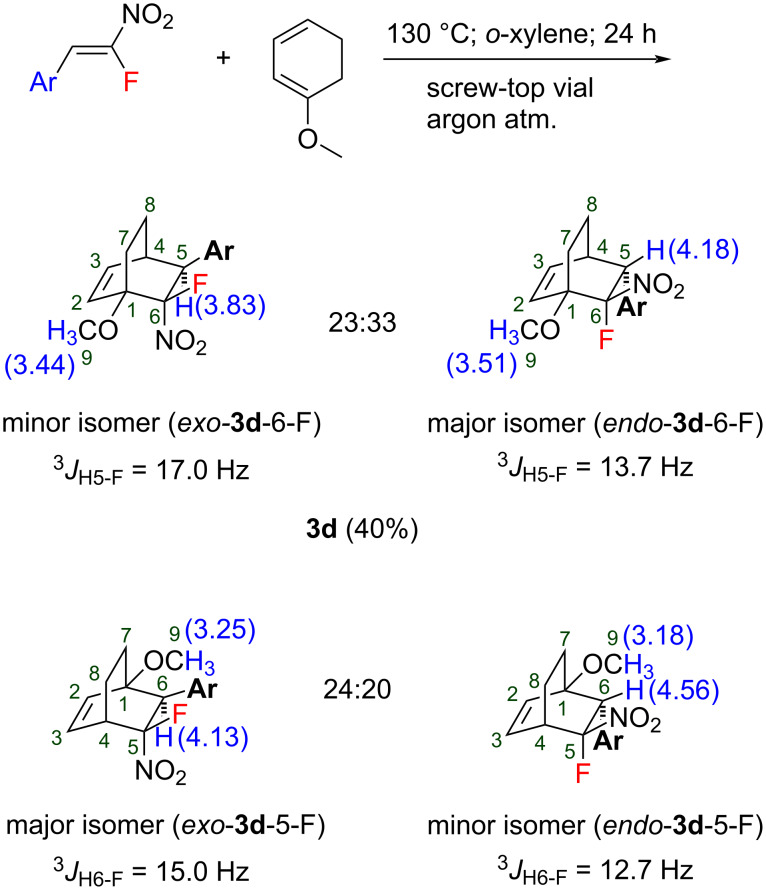
The Diels–Alder reaction of the nitrostyrene **1h** with 1-methoxy-1,3-cyclohexadiene.

Next, the reaction with some other cyclic dienes was investigated. The reaction with the unsymmetrical 1-methoxy-1,3-cyclohexadiene (Scheme 5) led to the formation of a mixture of four products (regioisomers and stereoisomers, respectively) **3d** in 40% overall yield. Two pairs of regioisomers were partially separated by column chromatography with sufficiently slow elution and analyzed by ^1^H NMR spectroscopy. The structure assignment was made as depicted in Scheme 5. The structures of two pairs of regioisomers were assigned by chemical shifts of the singlet of the methoxy group. The products having the MeO and NO_2_ groups in the adjacent position have the signal of the methoxy protons shifted to a lower field. The assignment of the *exo*/*endo*-isomers was carried out by the position of the benzylic proton (H5 or H6) and its coupling constant to fluorine (^3^*J*_H5-F_ or ^3^*J*_H6-F_).

The reaction with 7- and 8-membered cyclic dienes (1,3-cycloheptadiene and 1,3-cyclooctadiene) did not result in the formation of the corresponding cycloadducts confirming that the reaction is very sensitive to the structure of the dienes. Moreover, it was found that furan did not react with nitrostyrenes **1**.

**Scheme 6:**
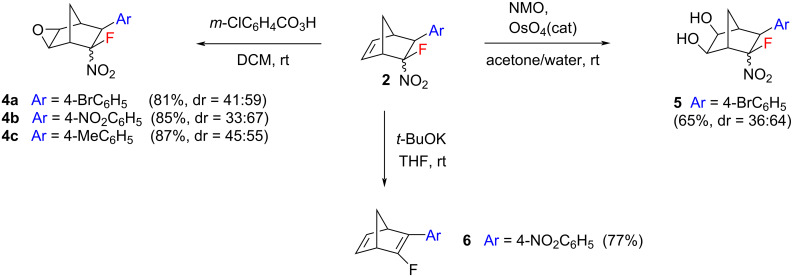
Selected chemical transformations of norbornenes **2** (dr = *exo*:*endo*).

Furthermore, we performed some subsequent transformations of the fluorinated norbornenes prepared to investigate their chemical properties and to demonstrate their utility (Scheme 6). These reactions were carried out to involve either the double bond or the nitro group of the norbornene products. The treatment of cycloadducts **2** with *m*-chloroperbenzoic acid afforded a series of novel fluorinated epoxynorbornane derivatives **4** in high yields (up to 87%). In all cases, the formation of mixtures of only two products was observed in ratios similar to those of the starting mixture **2**. We believe that this is a result of an *exo*-epoxidation which is preferred in norbornene systems [78–79]. Such a functionalization is very attractive to produce new reactive building blocks bearing the norbornane scaffold. This approach can pave a straightforward way to numerous fluorine-containing bicyclic compounds not previously available. The s*yn*-dihydroxylation of compound **2f** with the *N*-methylmorpholine-*N*-oxide (NMO)–OsO_4_ system resulted in a mixture of the corresponding diols **5** in a 36:64 ratio in 65% yield. Again, *exo*-dihydroxylation is to be expected [80–82]. The treatment of norbornene **2l** with *t*-BuOK resulted in the selective elimination of nitrous acid to form the desired monofluorinated norbornadiene **6** in 77% yield. No competitive elimination of HF was observed. The Diels–Alder reaction–base-induced HNO_2_ elimination sequence opens a straightforward way to novel fluorinated norbornadienes from β-fluoro-β-nitrostyrenes and CPD.

## Conclusion

In summary, the Diels–Alder reaction of β-fluoro-β-nitrostyrenes with cyclic 1,3-dienes was investigated. A series of novel monofluorinated norbornenes was prepared in high yield up to 97%. A number of novel monofluorinated bicyclo[2.2.2]oct-2-enes was obtained in up to 40% yield. The reactivity of CPD and its homologues was evaluated and compared. The reaction rate for CHD proved to be 267 times lower than that for CPD in a model reaction, whereas 1,3-cycloheptadiene and 1,3-cyclooctadiene were found to be unable to react. The activation parameters of the reaction of nitrostyrene **1h** with CPD were estimated. In addition, the synthetic utility of the norbornenes obtained was demonstrated. All the structures obtained in this work were elucidated by NMR spectroscopy and elemental analysis or HRMS.

## Supporting Information

File 1 Copies of spectra, experimental section, and computational details of DFT calculations.
