# Supplementary material for: Diels–Alder reaction of β-fluoro-β-nitrostyrenes with cyclic dienes
Source: Beilstein J Org Chem. 2021 Jan 27;17:283–92. doi: 10.3762/bjoc.17.27 (PMC7849230; doi:10.3762/bjoc.17.27)
Supplement: File 1 — Copies of spectra, experimental section, and computational details of DFT calculations. [file Beilstein_J_Org_Chem-17-283-s001.pdf]

BEILSTEIN JOURNAL OF ORGANIC CHEMISTRY

Supporting Information

for

Diels–Alder reaction of β-fluoro-β-nitrostyrenes with cyclic dienes

Savva A. Ponomarev, Roman V. Larkovich, Alexander S. Aldoshin, Andrey A. Tabolin, Sema L. Ioffe, Jonathan Groß, Till Opatz and Valentine G. Nenajdenko

Beilstein J. Org. Chem. **2021,** *17,* 283–292. doi:10.3762/bjoc.17.27

Copies of spectra, experimental section, and computational details of DFT calculations

License and Terms: This is a supporting information file under the terms of the Creative Commons Attribution License (https://creativecommons.org/licenses/by/4.0). Please note that the reuse, redistribution and reproduction in particular requires that the author(s) and source are credited and that individual graphics may be subject to special legal provisions.
The license is subject to the *Beilstein Journal of Organic Chemistry* terms and conditions: (https://www.beilstein-journals.org/bjoc/terms)

Table of contents

1. NMR spectra....................S2

5-Fluoro-5-nitro-6-phenylbicyclo[2.2.1]hept-2-ene (**2a**)....................S2

5-Fluoro-5-nitro-6-(p-tolyl)bicyclo[2.2.1]hept-2-ene (**2b**)....................S5

6-(4-(*Tert*-butyl)phenyl)-5-fluoro-5-nitrobicyclo[2.2.1]hept-2-ene (**2c**)....................S8

6-(4-Chlorophenyl)-5-fluoro-5-nitrobicyclo[2.2.1]hept-2-ene (**2d**)....................S11

6-(2,4-Dichlorophenyl)-5-fluoro-5-nitrobicyclo[2.2.1]hept-2-ene (**2e**)....................S14

6-(4-Bromophenyl)-5-fluoro-5-nitrobicyclo[2.2.1]hept-2-ene (**2f**)....................S17

5-Fluoro-6-(4-methoxyphenyl)-5-nitrobicyclo[2.2.1]hept-2-ene (**2g**)....................S20

Methyl 4-(3-fluoro-3-nitrobicyclo[2.2.1]hept-5-en-2-yl)benzoate (**2h**)....................S23

5-Fluoro-5-nitro-6-(4-(trifluoromethyl)phenyl)bicyclo[2.2.1]hept-2-ene (**2i**)....................S26

4-(3-Fluoro-3-nitrobicyclo[2.2.1]hept-5-en-2-yl)benzonitrile (**2j**)....................S29

5-Fluoro-5-nitro-6-(3-nitrophenyl)bicyclo[2.2.1]hept-2-ene (**2k**)....................S32

5-Fluoro-5-nitro-6-(4-nitrophenyl)bicyclo[2.2.1]hept-2-ene (**2l**)....................S35

Methyl 4-(5-fluoro-5-nitrospiro[bicyclo[2.2.1]hept[2]ene-7,1'-cyclopropan]-6-yl)benzoate (**2m**)........S38

6-(2,4-Dichlorophenyl)-5-fluoro-5-nitrobicyclo[2.2.2]oct-2-ene (**3a**)....................S41

Methyl 4-(3-fluoro-3-nitrobicyclo[2.2.2]oct-5-en-2-yl)benzoate (**3b**)....................S44

5-Fluoro-5-nitro-6-(4-nitrophenyl)bicyclo[2.2.2]oct-2-ene (**3c**)....................S47

Methyl 4-(3-fluoro-4-methoxy-3-nitrobicyclo[2.2.2]oct-5-en-2-yl)benzoate and
methyl 4-(3-fluoro-1-methoxy-3-nitrobicyclo[2.2.2]oct-5-en-2-yl)benzoate (**3d**)....................S50

7-(4-Bromophenyl)-6-fluoro-6-nitro-3-oxatricyclo[3.2.1.0^{2,4}]octane (**4a**)....................S57

6-Fluoro-6-nitro-7-(4-nitrophenyl)-3-oxatricyclo[3.2.1.0^{2,4}]octane (**4b**)....................S60

6-Fluoro-6-nitro-7-(p-tolyl)-3-oxatricyclo[3.2.1.0^{2,4}]octane (**4c**)....................S65

6-(4-Bromophenyl)-5-fluoro-5-nitrobicyclo[2.2.1]heptane-2,3-diol (**5**)....................S67

2-Fluoro-3-(4-nitrophenyl)bicyclo[2.2.1]hepta-2,5-diene (**6**)....................S70

2. Experimental section....................S72

3. Computational details of DFT calculations....................S85

4. References....................S91

1. NMR spectra

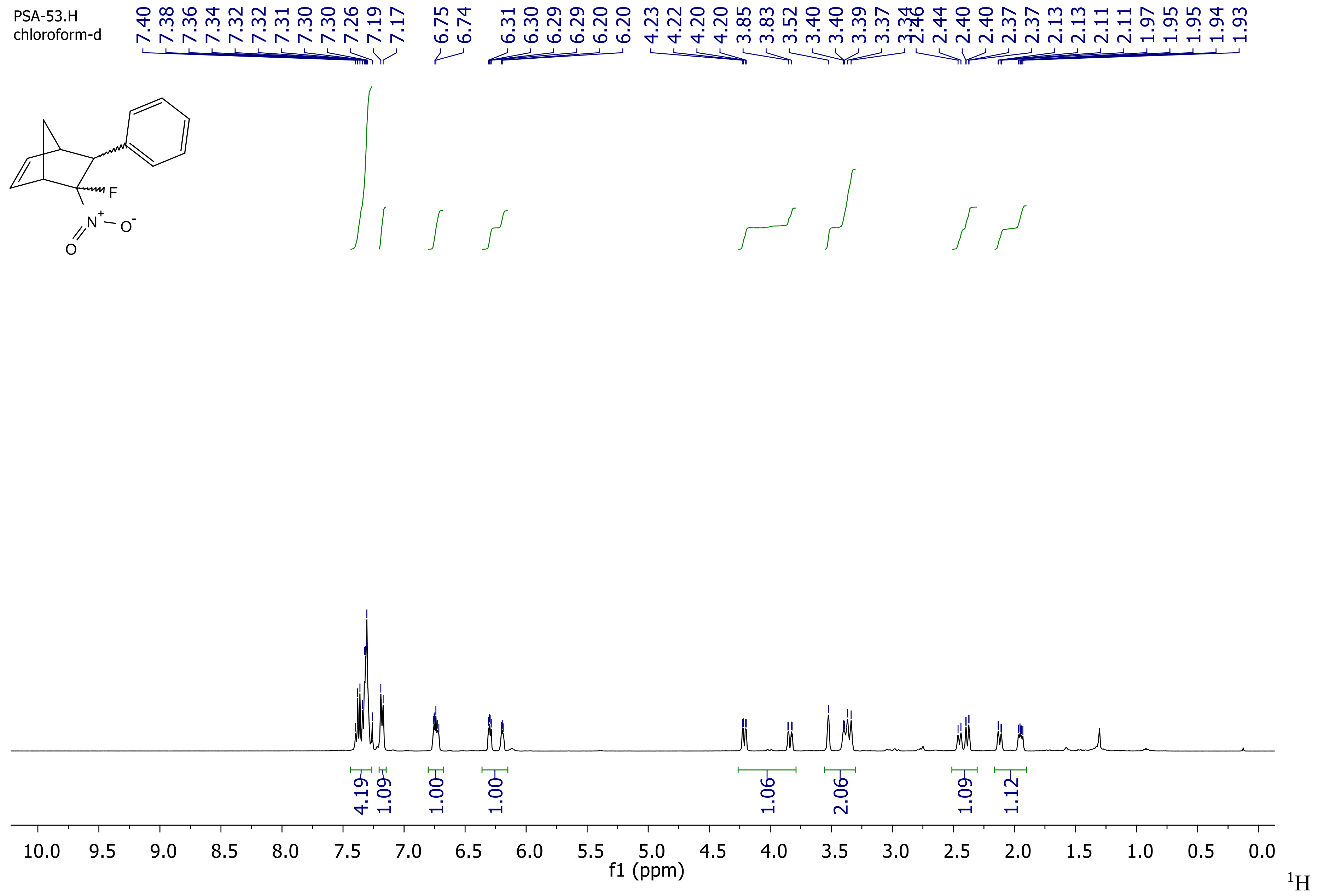

NMR spectrum of 5-fluoro-5-nitro-6-phenylbicyclo[2.2.1]hept-2-ene (**2a**)

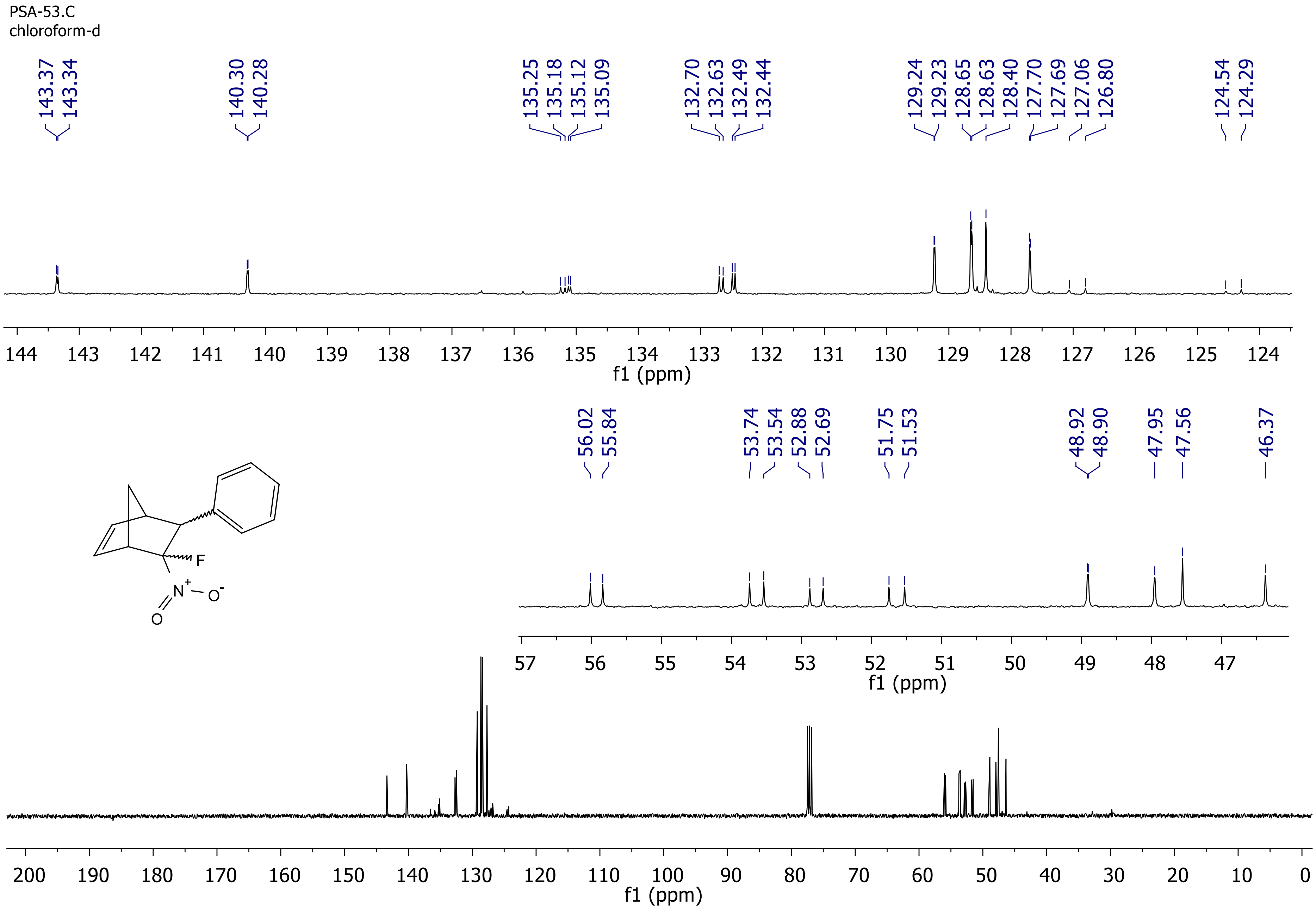

^{13}C NMR spectrum of 5-fluoro-5-nitro-6-phenylbicyclo[2.2.1]hept-2-ene (**2a**)

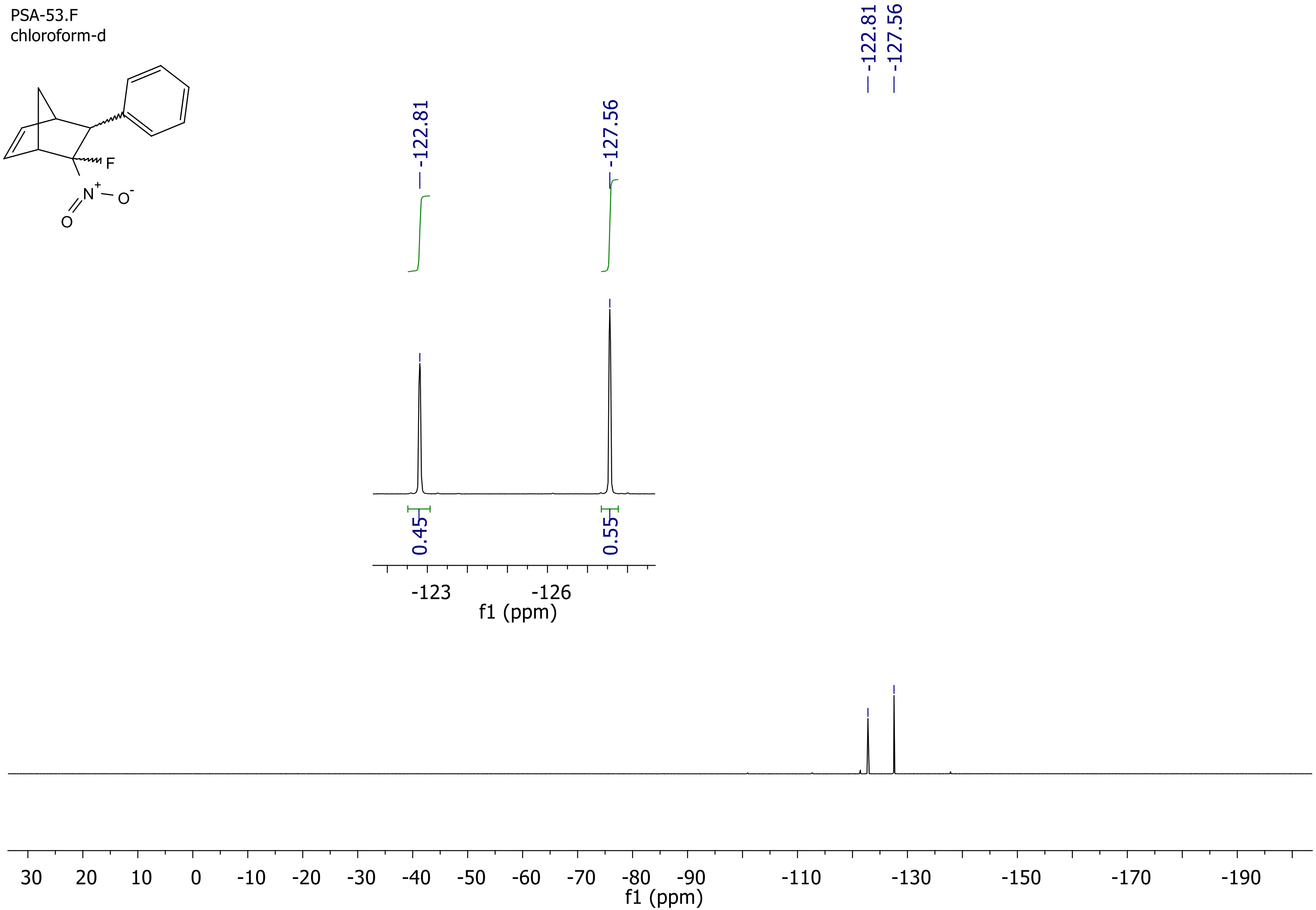

^{19}F NMR spectrum of 5-fluoro-5-nitro-6-phenylbicyclo[2.2.1]hept-2-ene (**2a**)

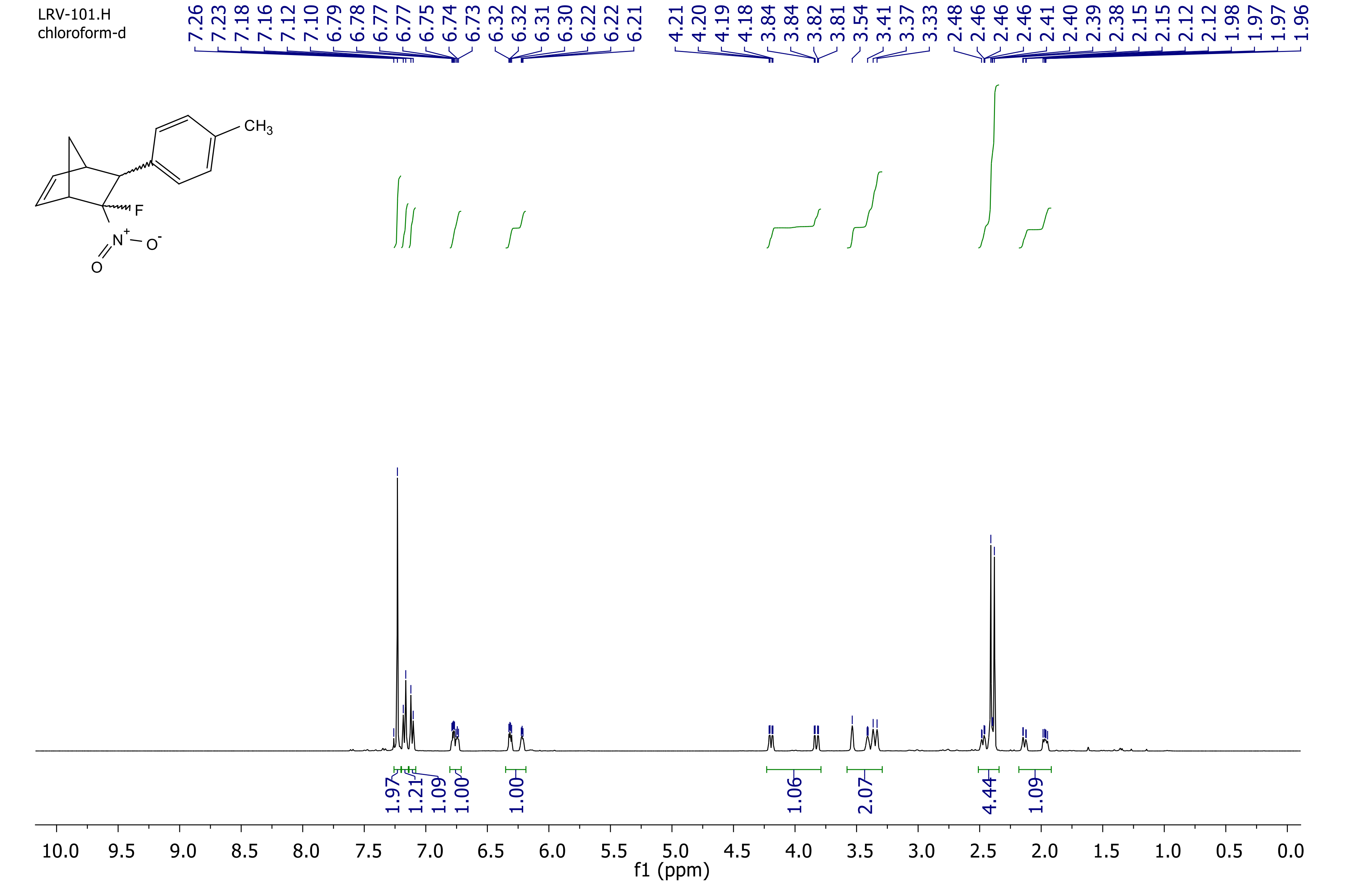

^{1}H NMR spectrum of 5-fluoro-5-nitro-6-(p-tolyl)bicyclo[2.2.1]hept-2-ene (**2b**)

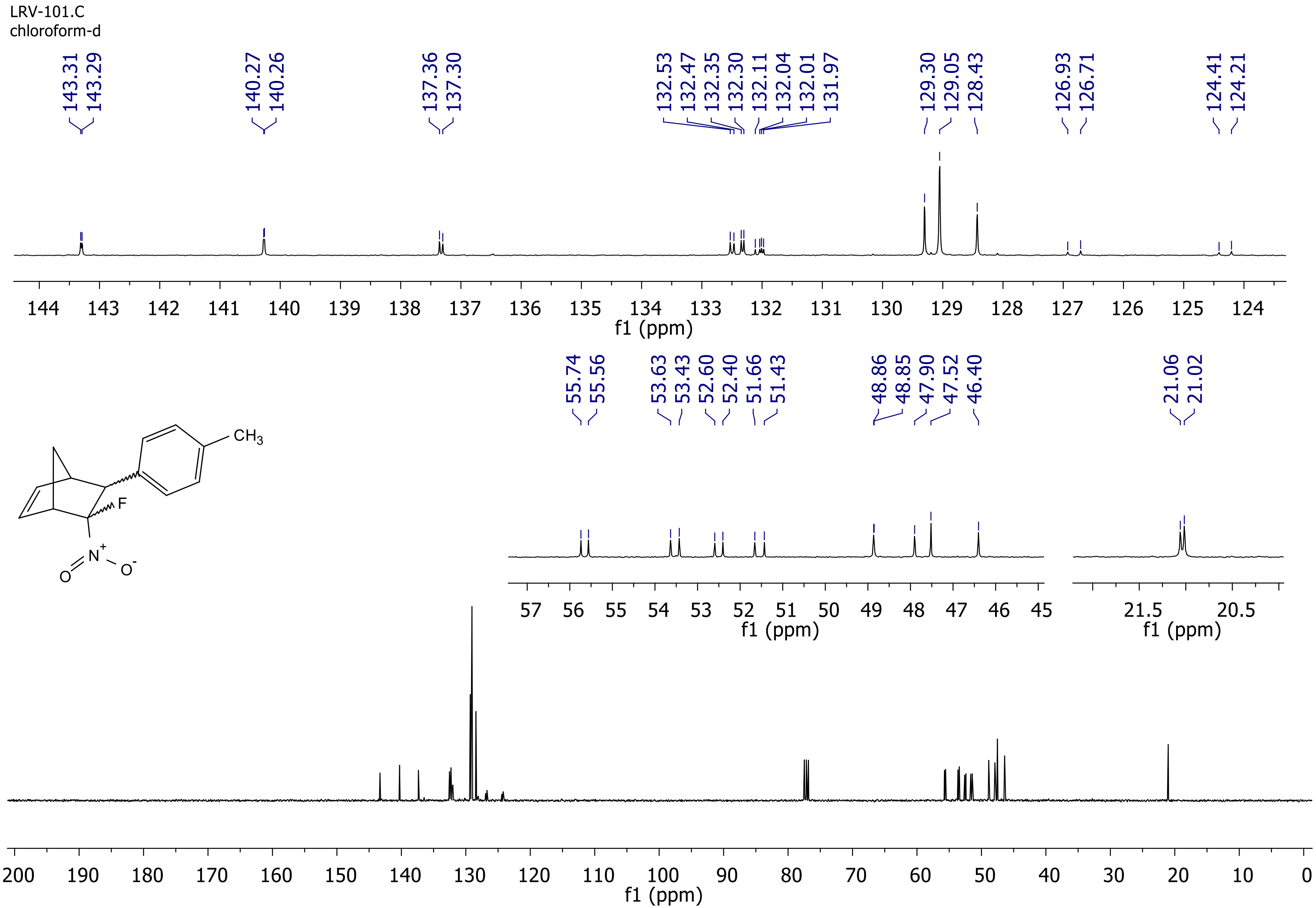

^{13}C NMR spectrum of 5-fluoro-5-nitro-6-(p-tolyl)bicyclo[2.2.1]hept-2-ene (**2b**)

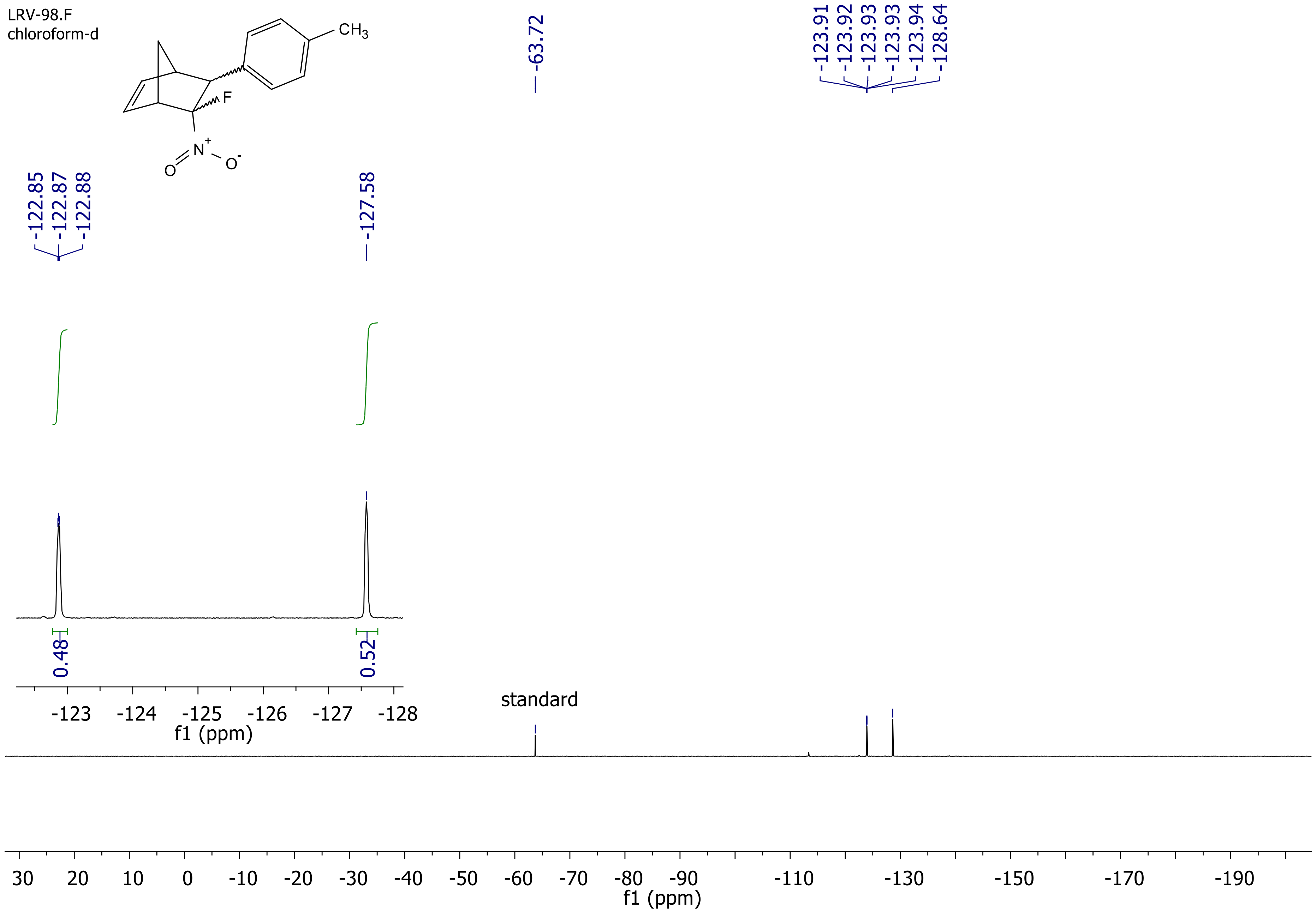

^{19}F NMR spectrum of 5-fluoro-5-nitro-6-(p-tolyl)bicyclo[2.2.1]hept-2-ene (**2b**)

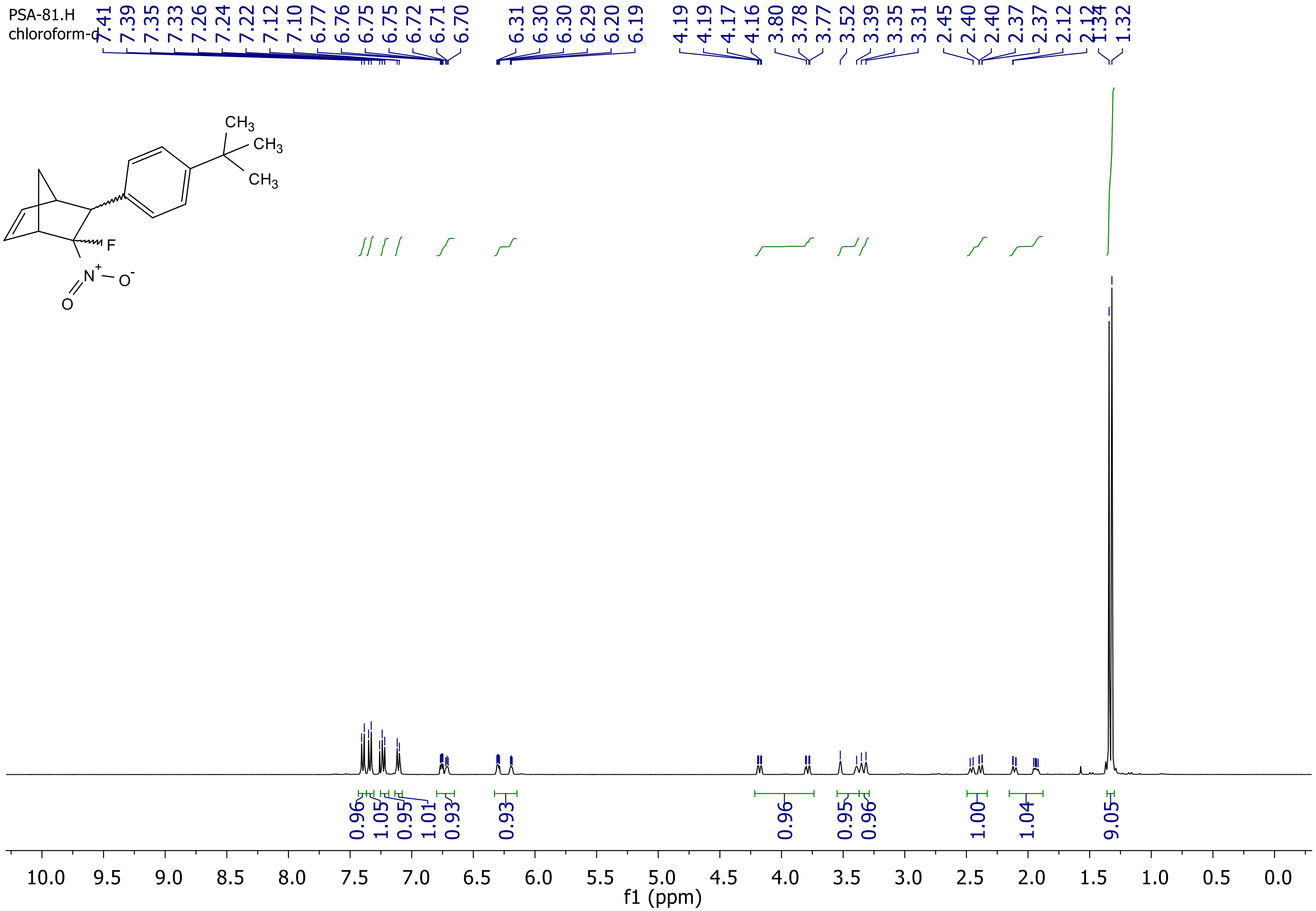

^{1}H NMR spectrum of 6-(4-(tert-butyl)phenyl)-5-fluoro-5-nitrobicyclo[2.2.1]hept-2-ene (**2c**)

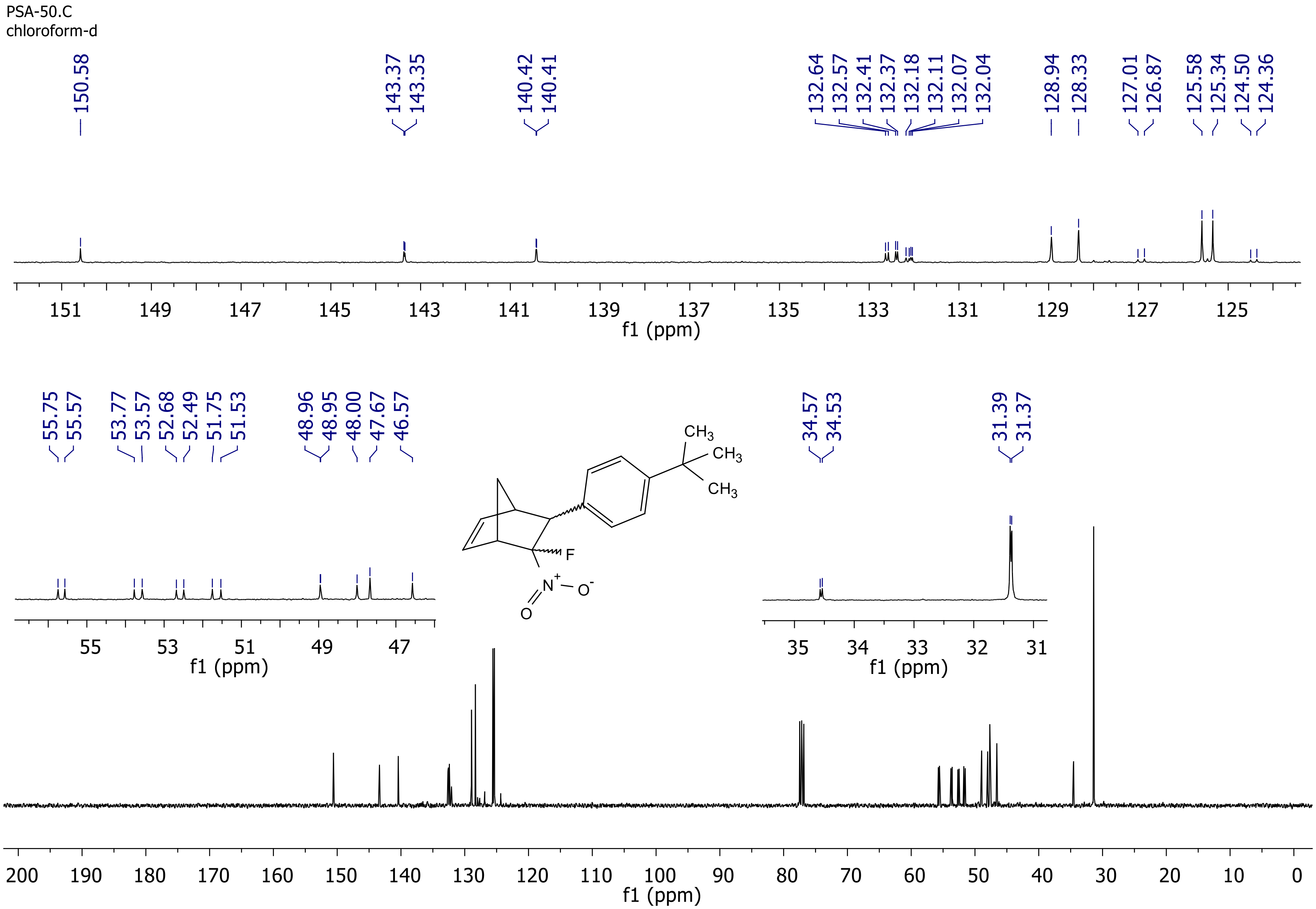

^{13}C NMR spectrum of 6-(4-(tert-butyl)phenyl)-5-fluoro-5-nitrobicyclo[2.2.1]hept-2-ene (**2c**)

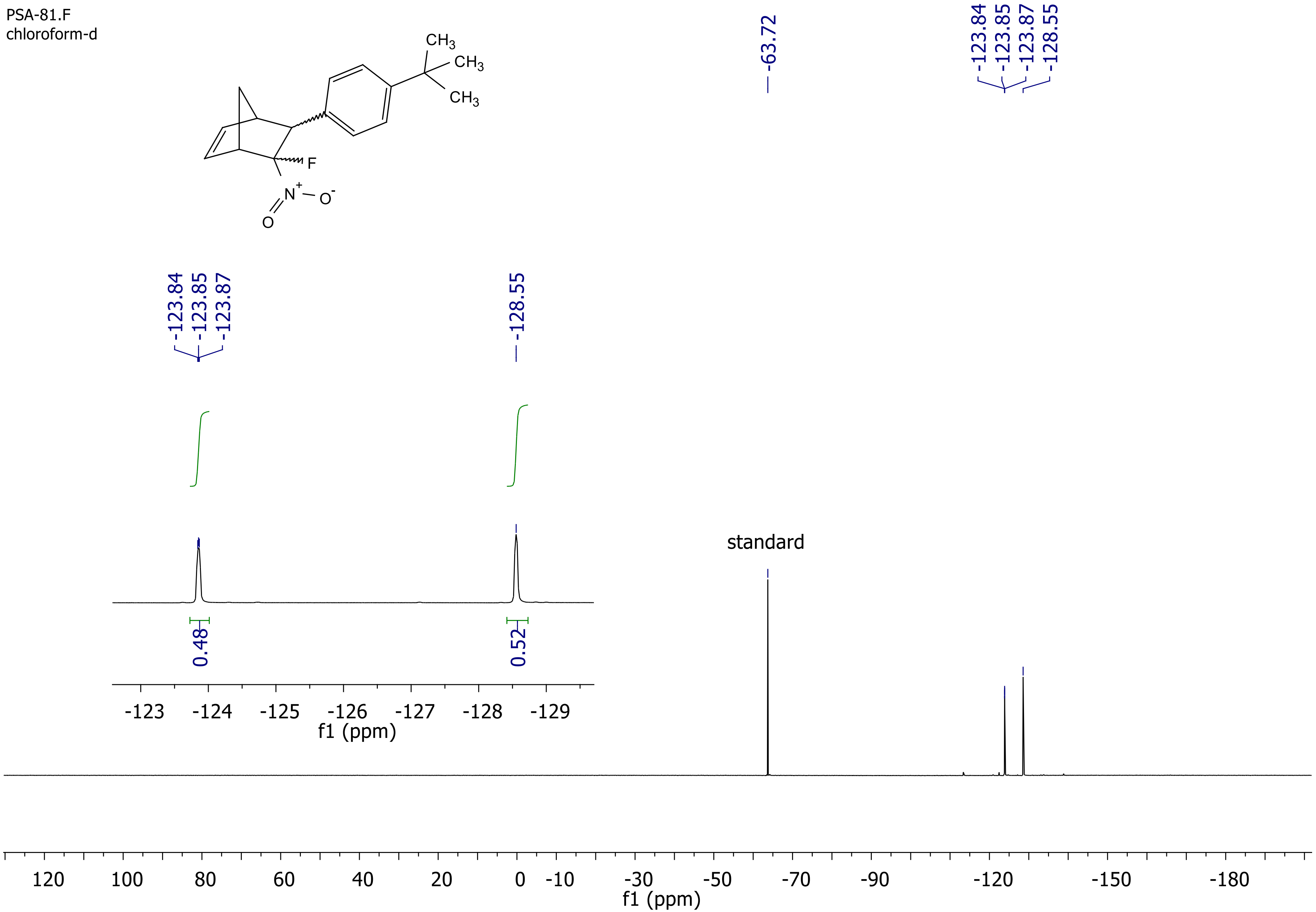

^{19}F NMR spectrum of 6-(4-(tert-butyl)phenyl)-5-fluoro-5-nitrobicyclo[2.2.1]hept-2-ene (**2c**)

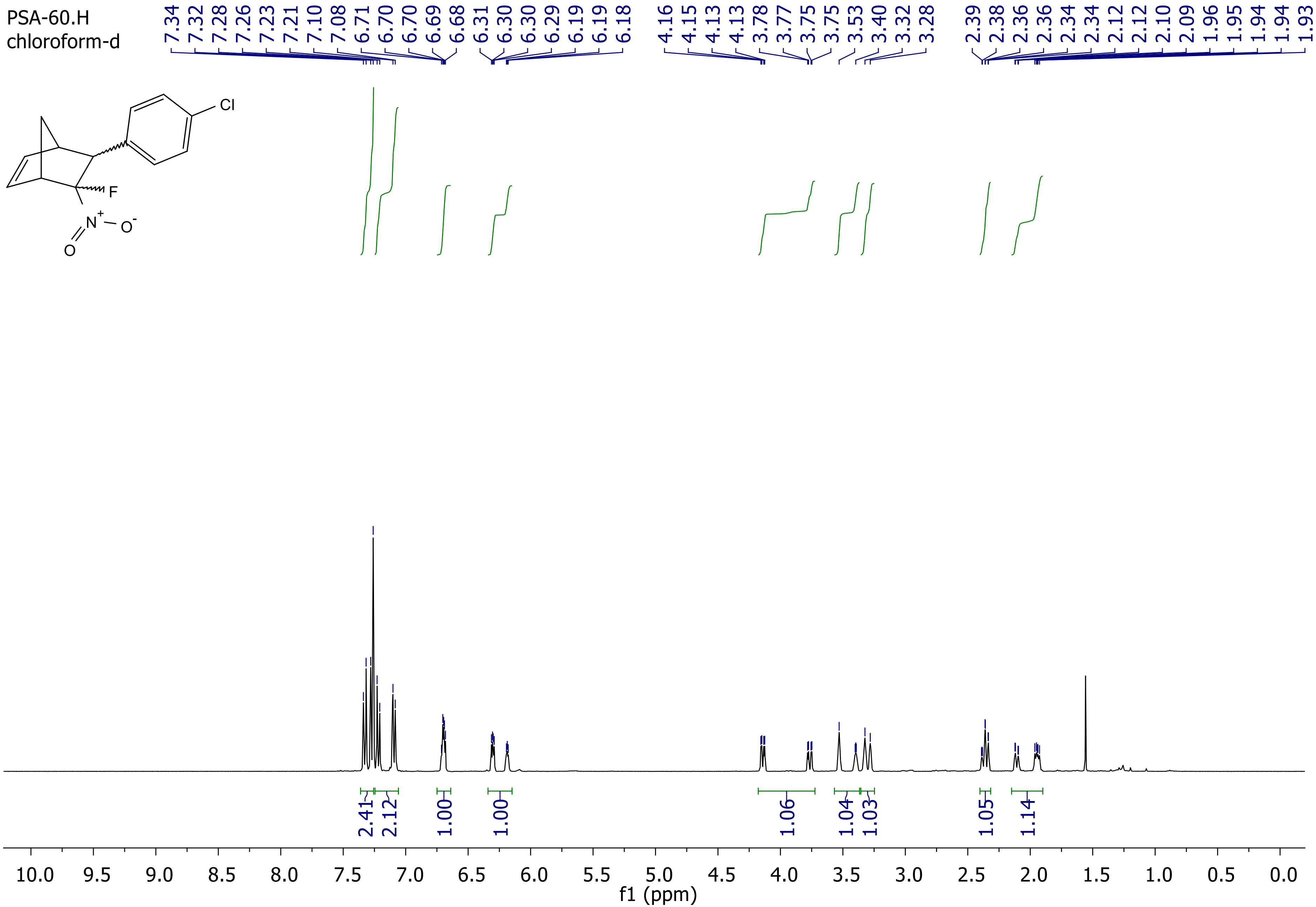

^{1}H NMR spectrum of 6-(4-chlorophenyl)-5-fluoro-5-nitrobicyclo[2.2.1]hept-2-ene (**2d**)

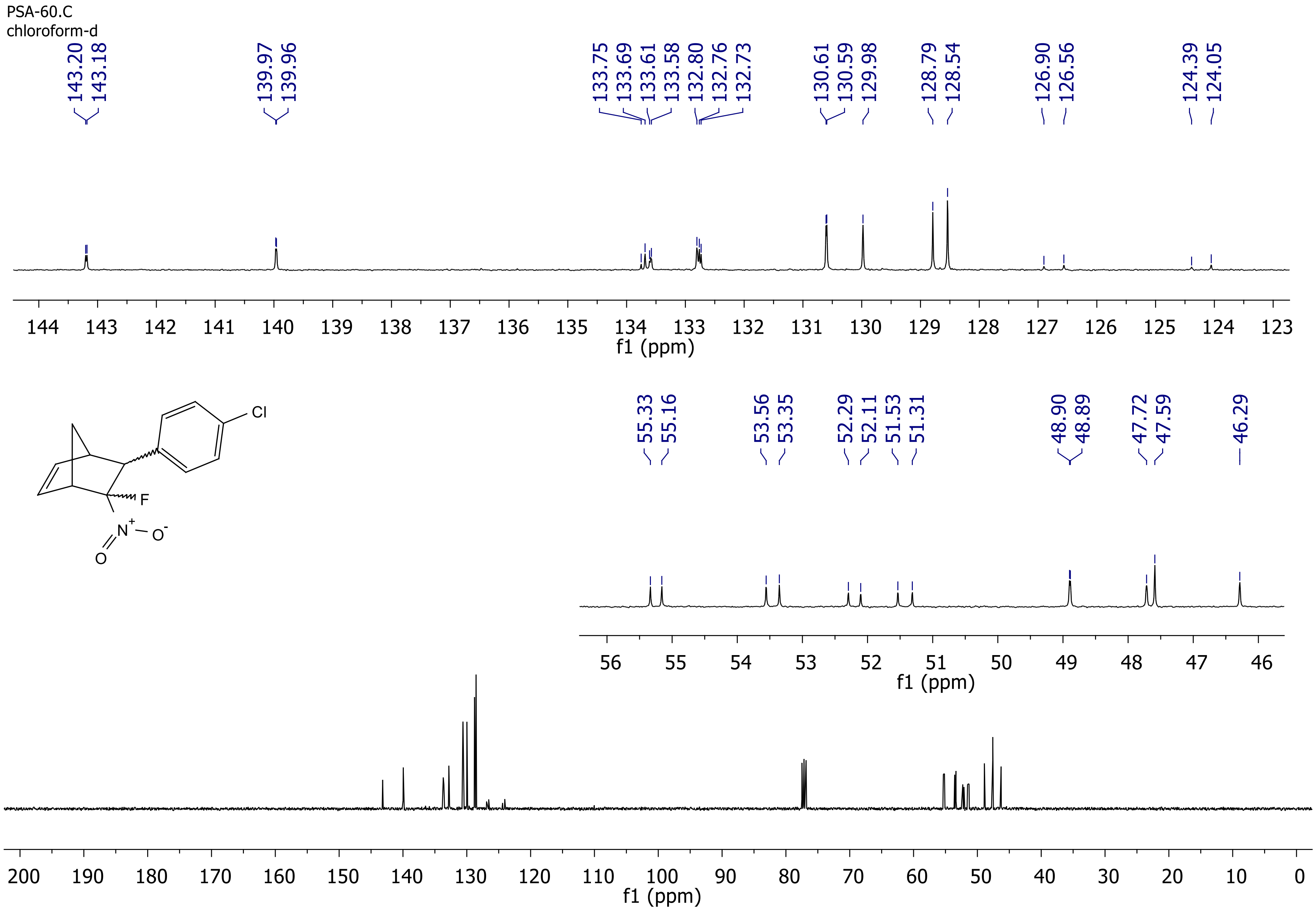

^{13}C NMR spectrum of 6-(4-chlorophenyl)-5-fluoro-5-nitrobicyclo[2.2.1]hept-2-ene (**2d**)

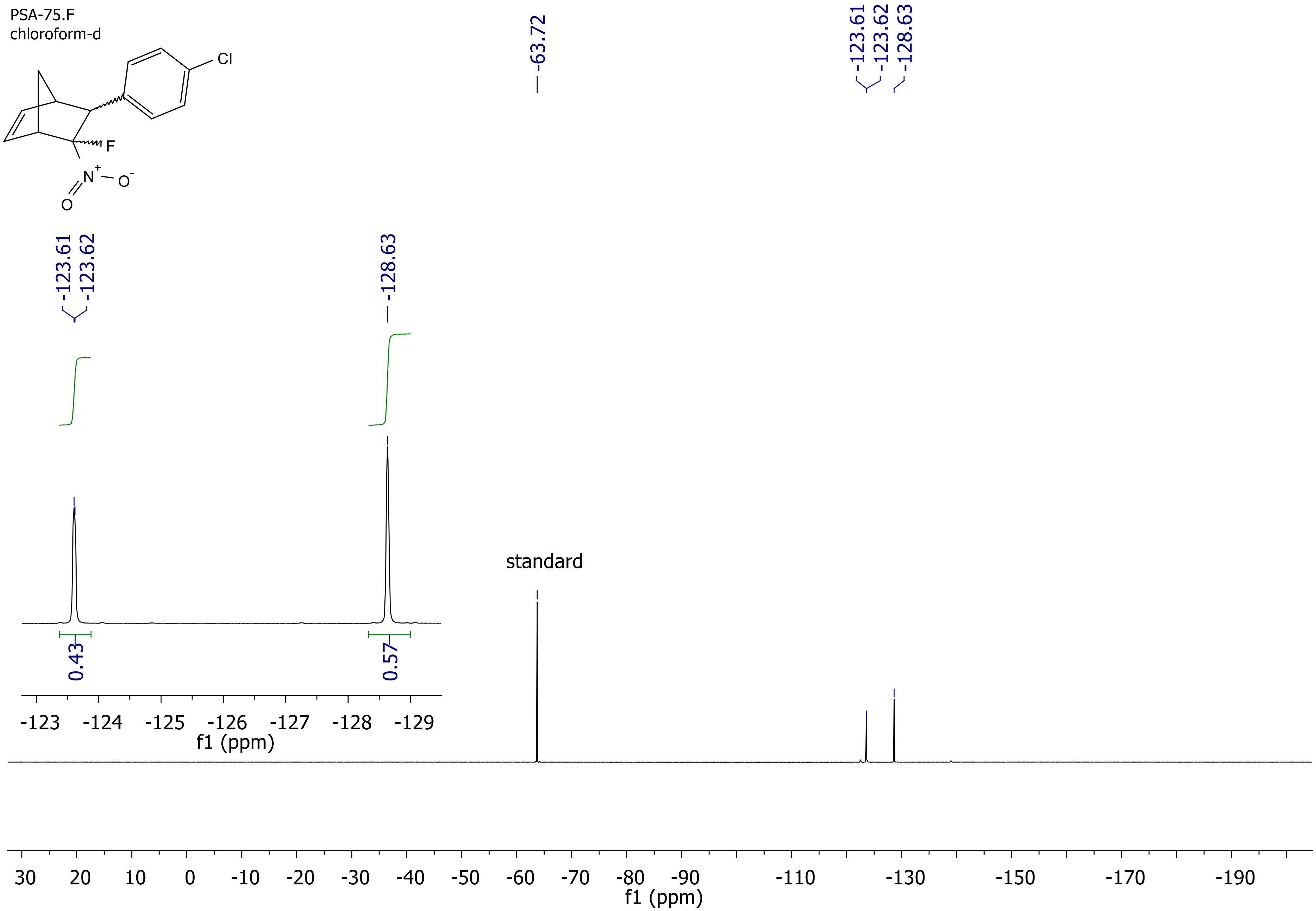

^{19}F NMR spectrum of 6-(4-chlorophenyl)-5-fluoro-5-nitrobicyclo[2.2.1]hept-2-ene (**2d**)

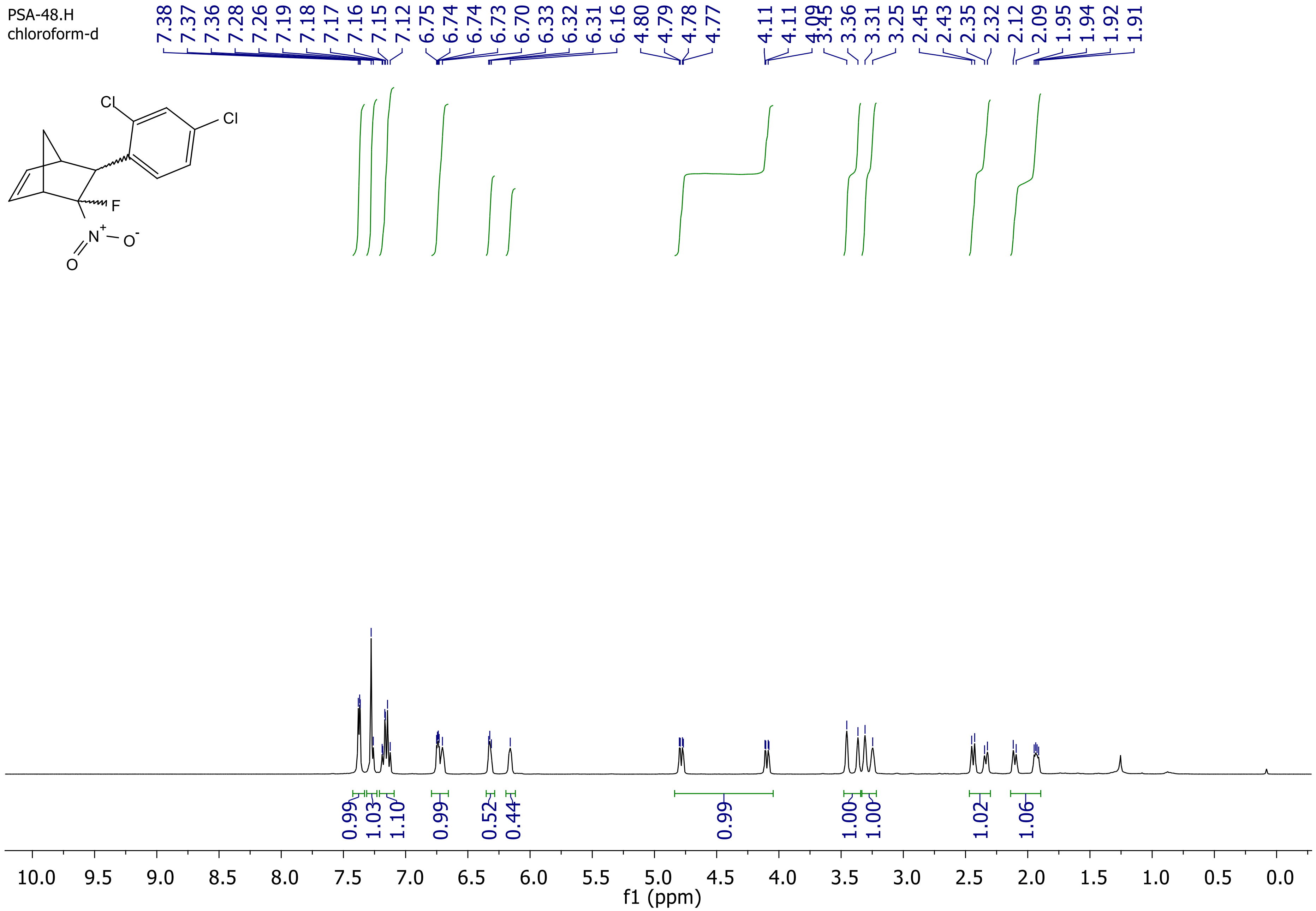

^{1}H NMR spectrum of 6-(2,4-dichlorophenyl)-5-fluoro-5-nitrobicyclo[2.2.1]hept-2-ene (**2e**)

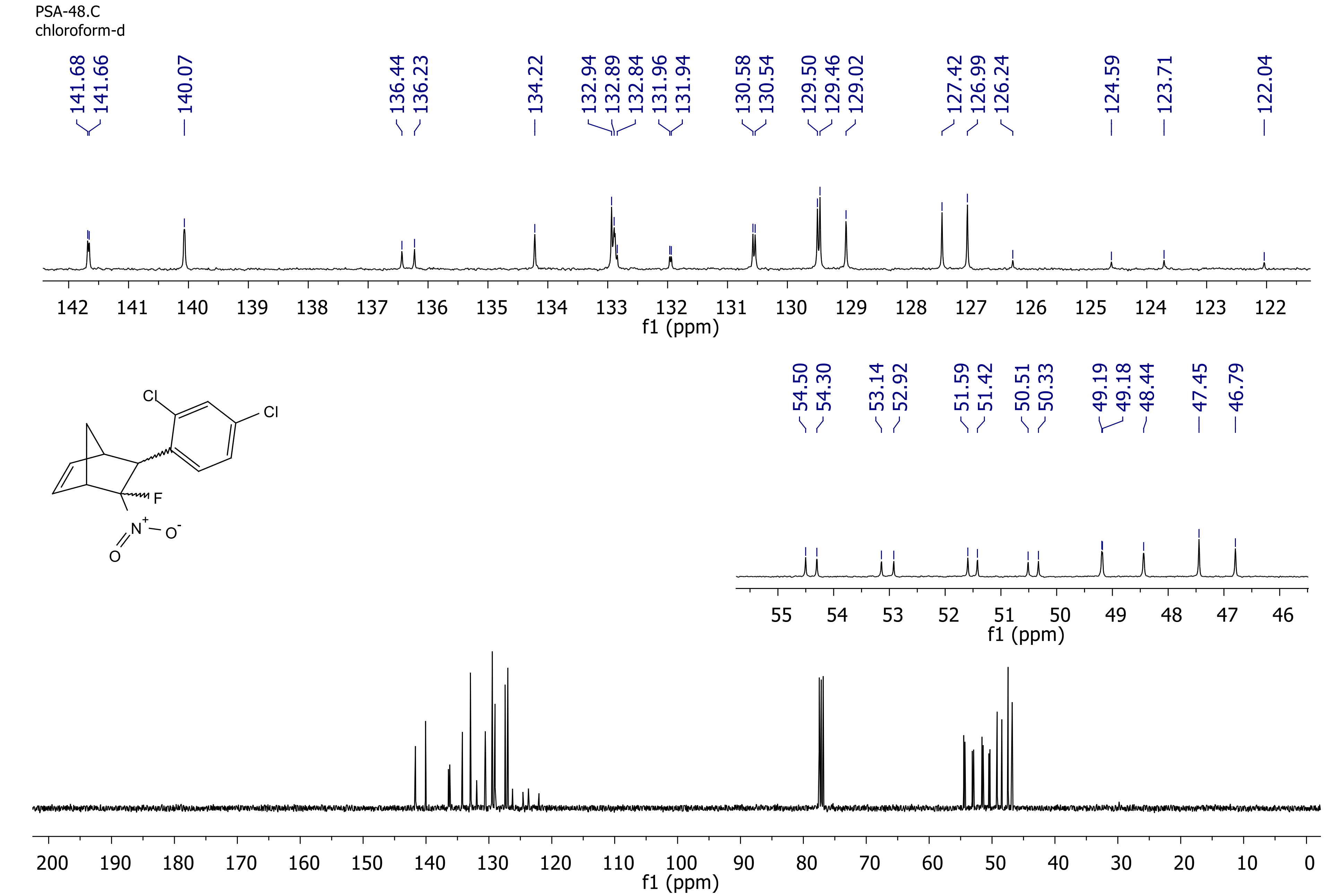

^{13}C NMR spectrum of 6-(2,4-dichlorophenyl)-5-fluoro-5-nitrobicyclo[2.2.1]hept-2-ene (**2e**)

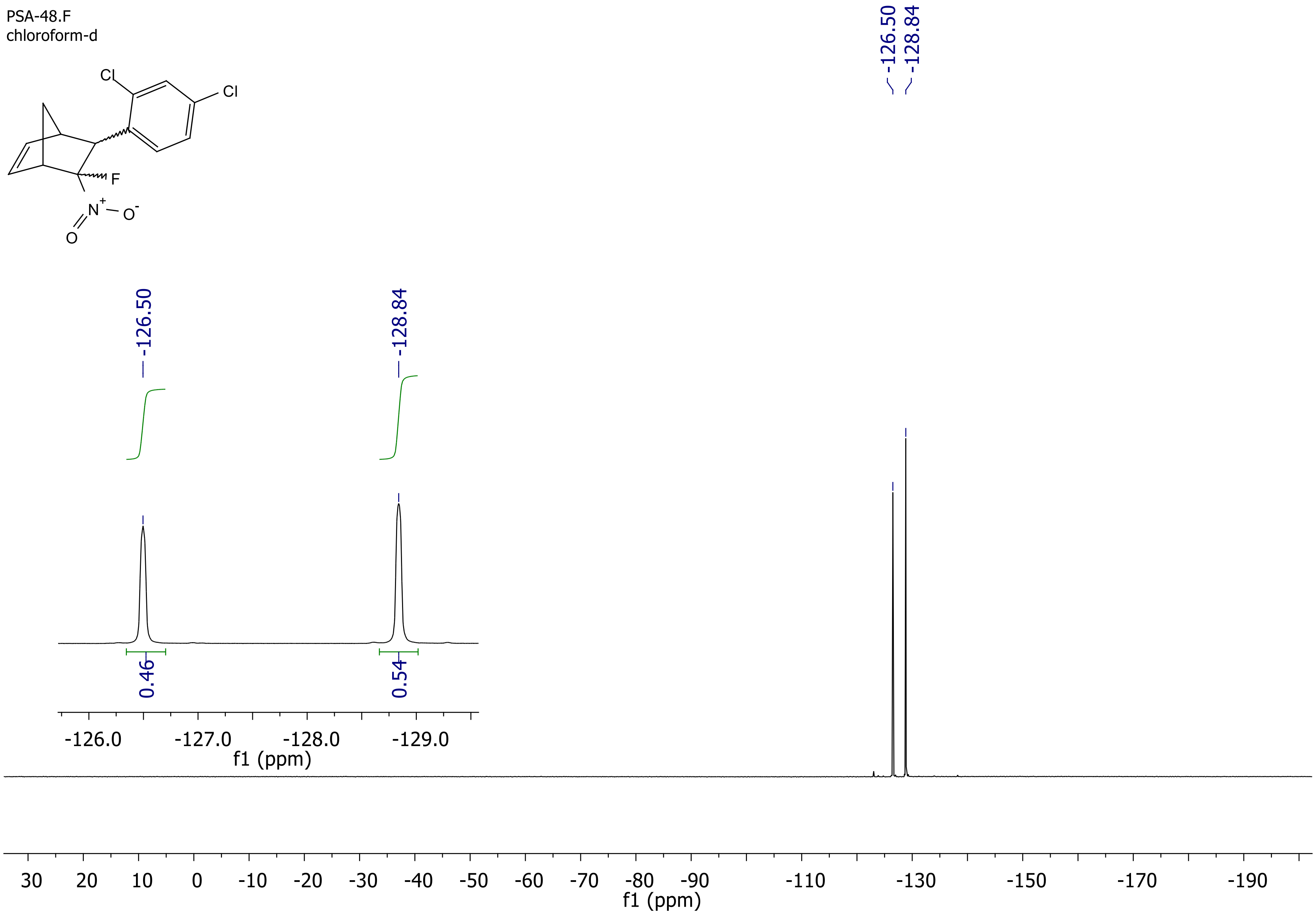

^{19}F NMR spectrum of 6-(2,4-dichlorophenyl)-5-fluoro-5-nitrobicyclo[2.2.1]hept-2-ene (**2e**)

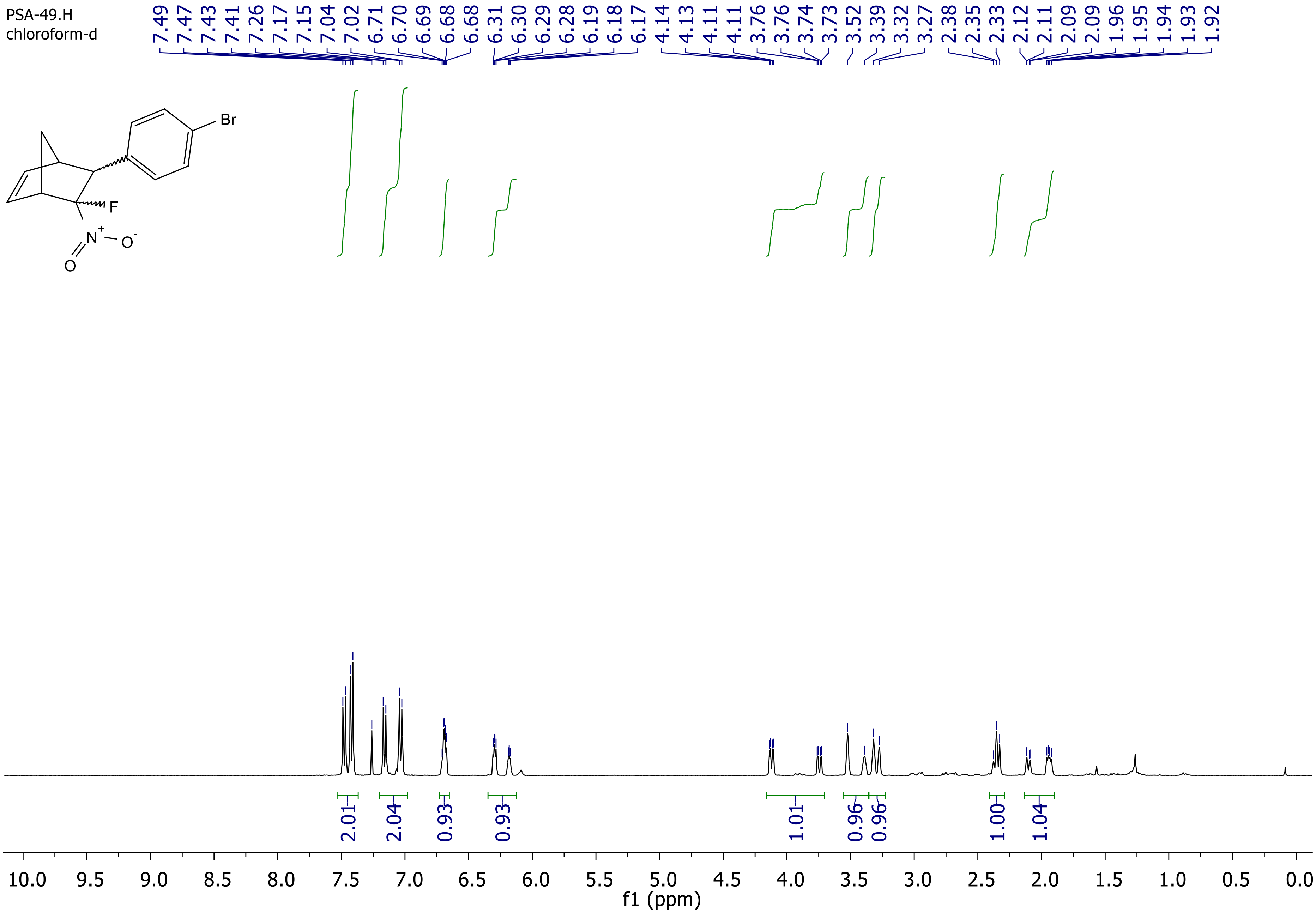

^{1}H NMR spectrum of 6-(4-bromophenyl)-5-fluoro-5-nitrobicyclo[2.2.1]hept-2-ene (**2f**)

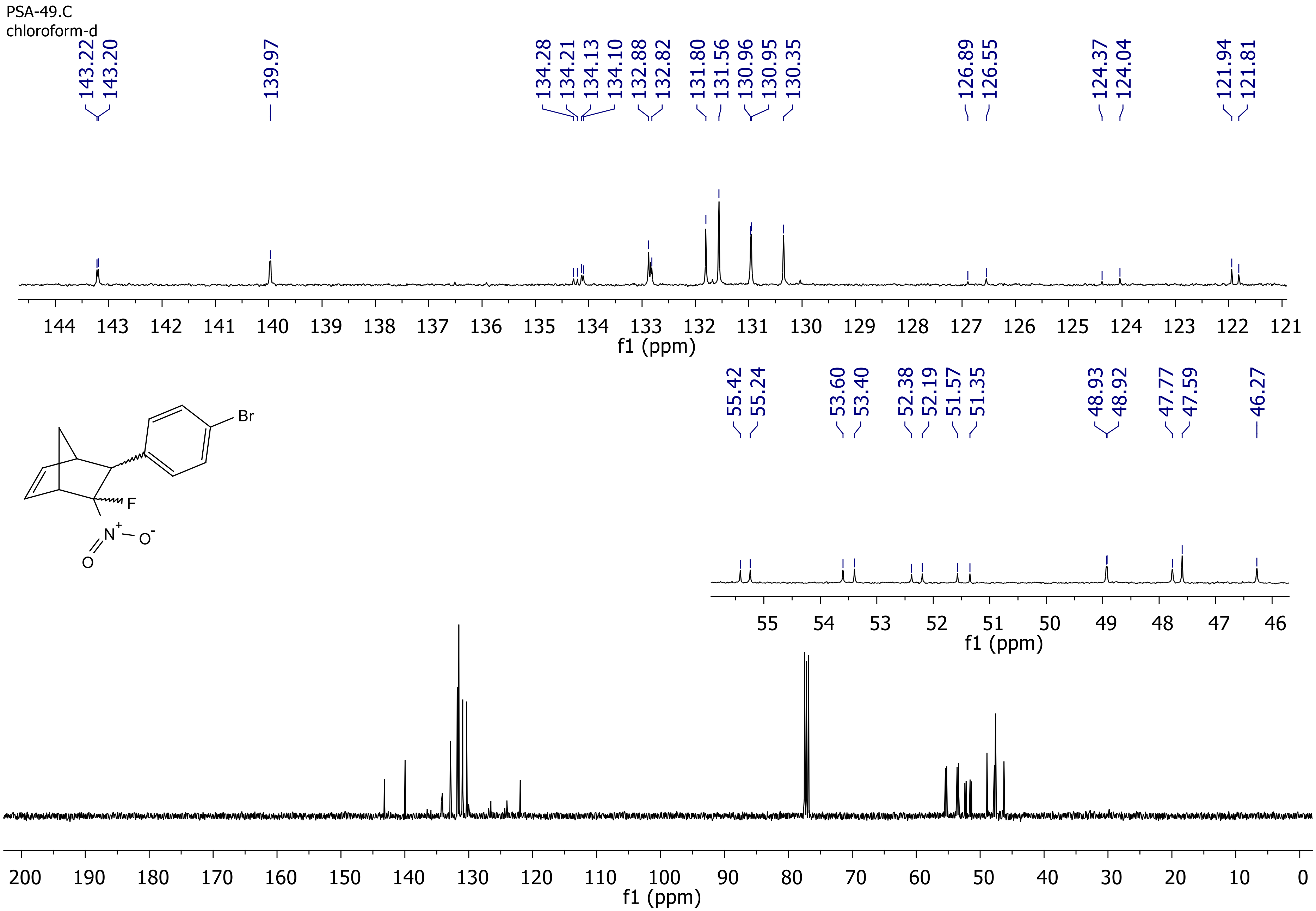

^{13}C NMR spectrum of 6-(4-bromophenyl)-5-fluoro-5-nitrobicyclo[2.2.1]hept-2-ene (**2f**)

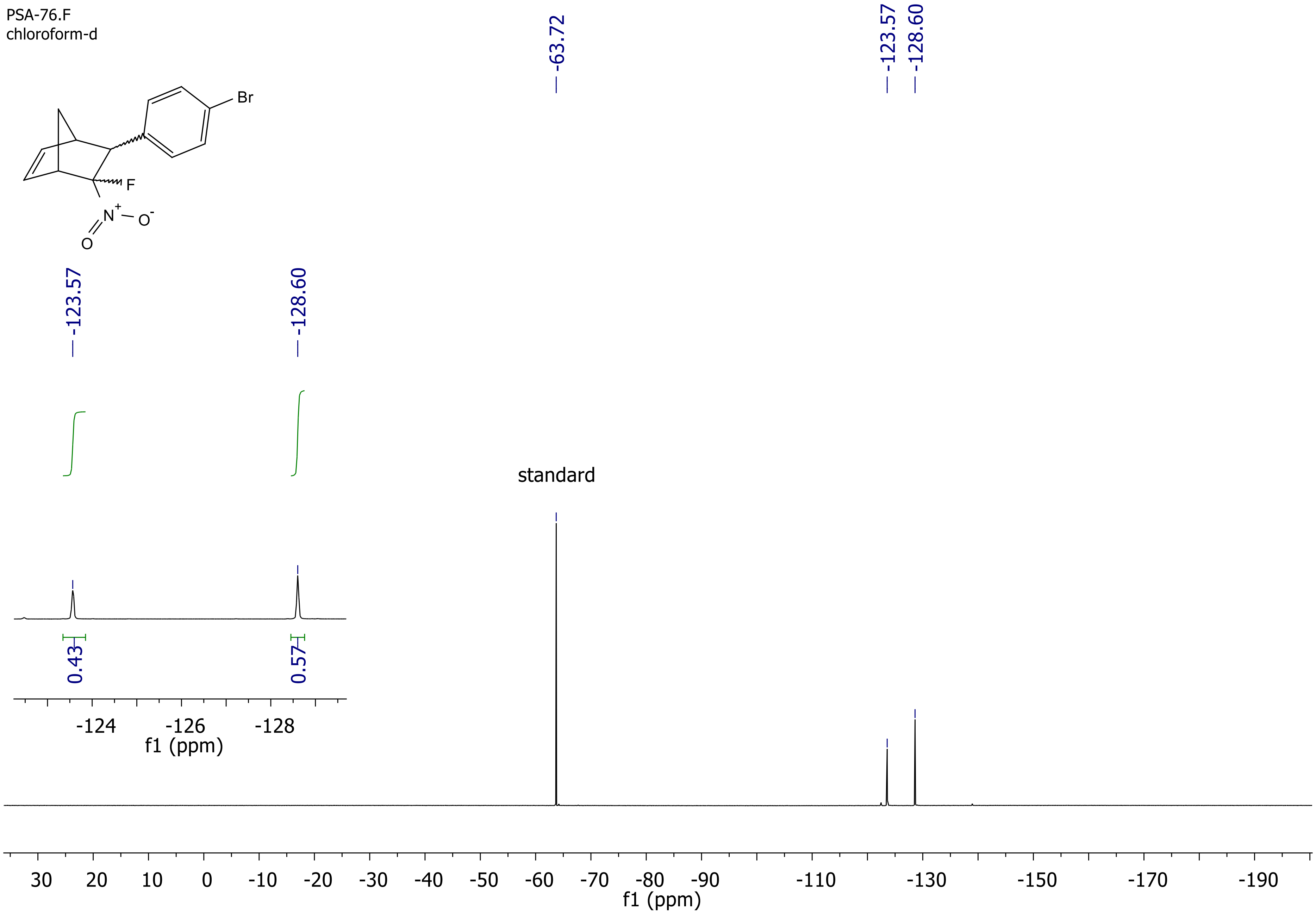

^{19}F NMR spectrum of 6-(4-bromophenyl)-5-fluoro-5-nitrobicyclo[2.2.1]hept-2-ene (**2f**)

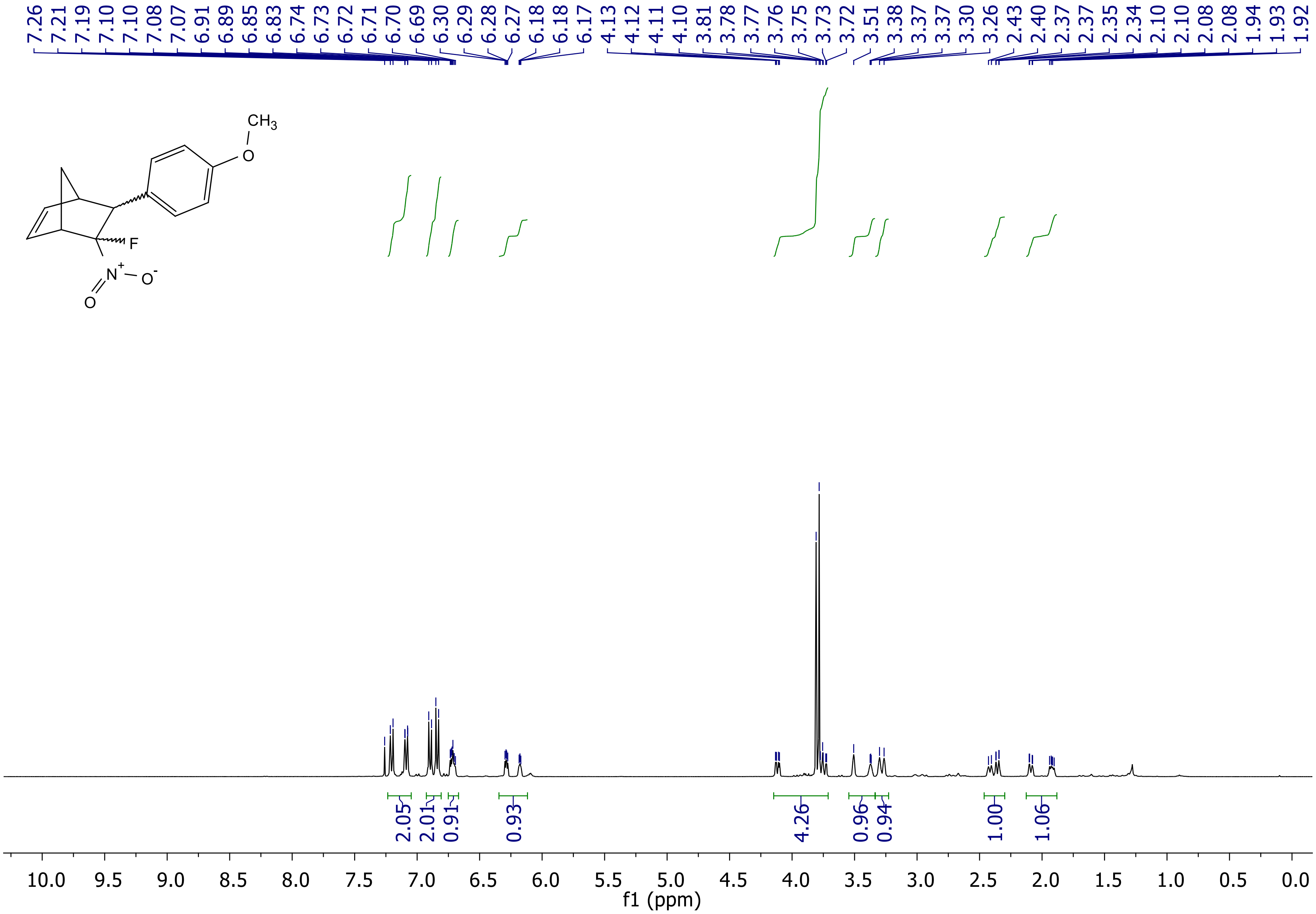

^{1}H NMR spectrum of 5-fluoro-6-(4-methoxyphenyl)-5-nitrobicyclo[2.2.1]hept-2-ene (**2g**)

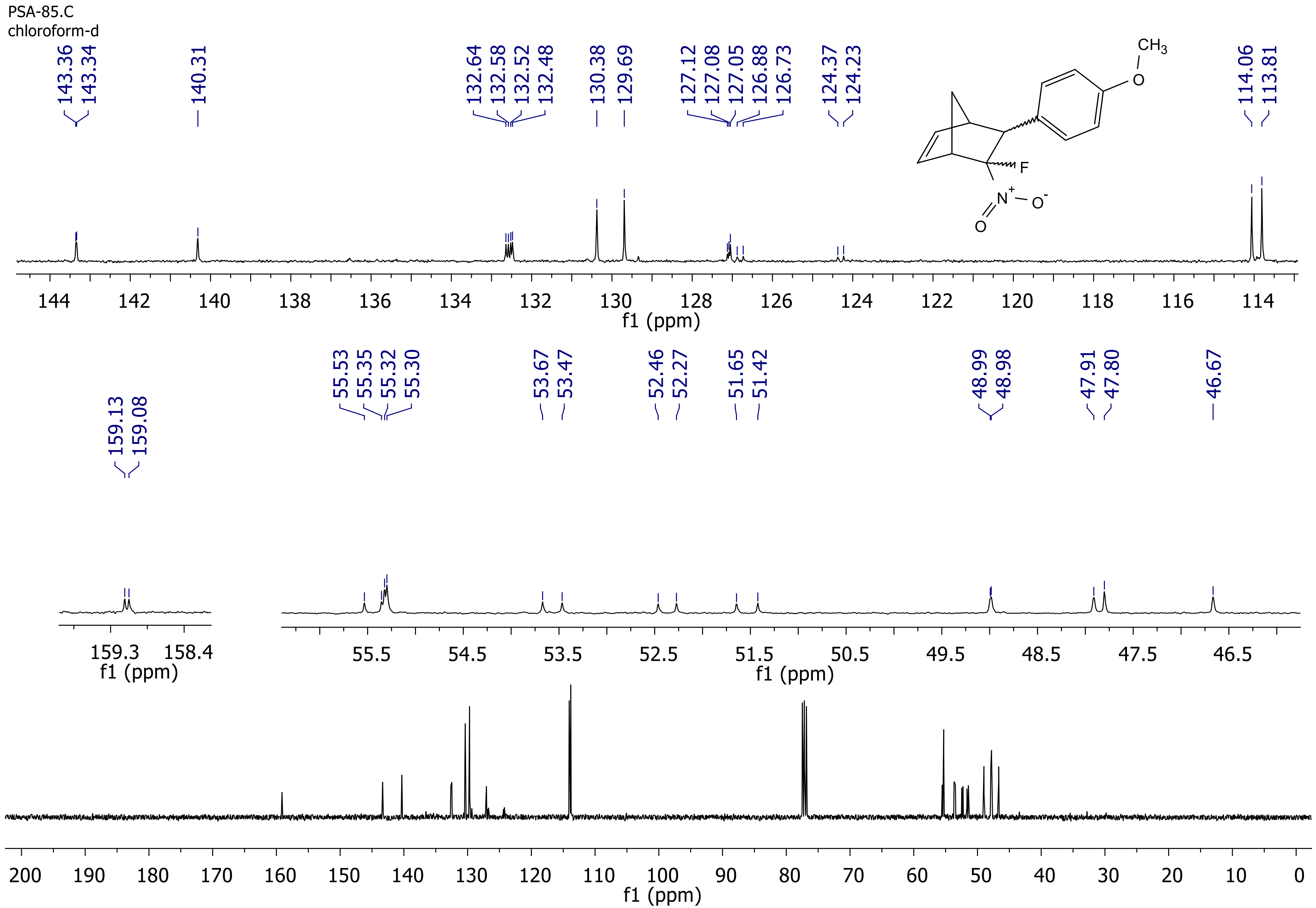

^{13}C NMR spectrum of 5-fluoro-6-(4-methoxyphenyl)-5-nitrobicyclo[2.2.1]hept-2-ene (**2g**)

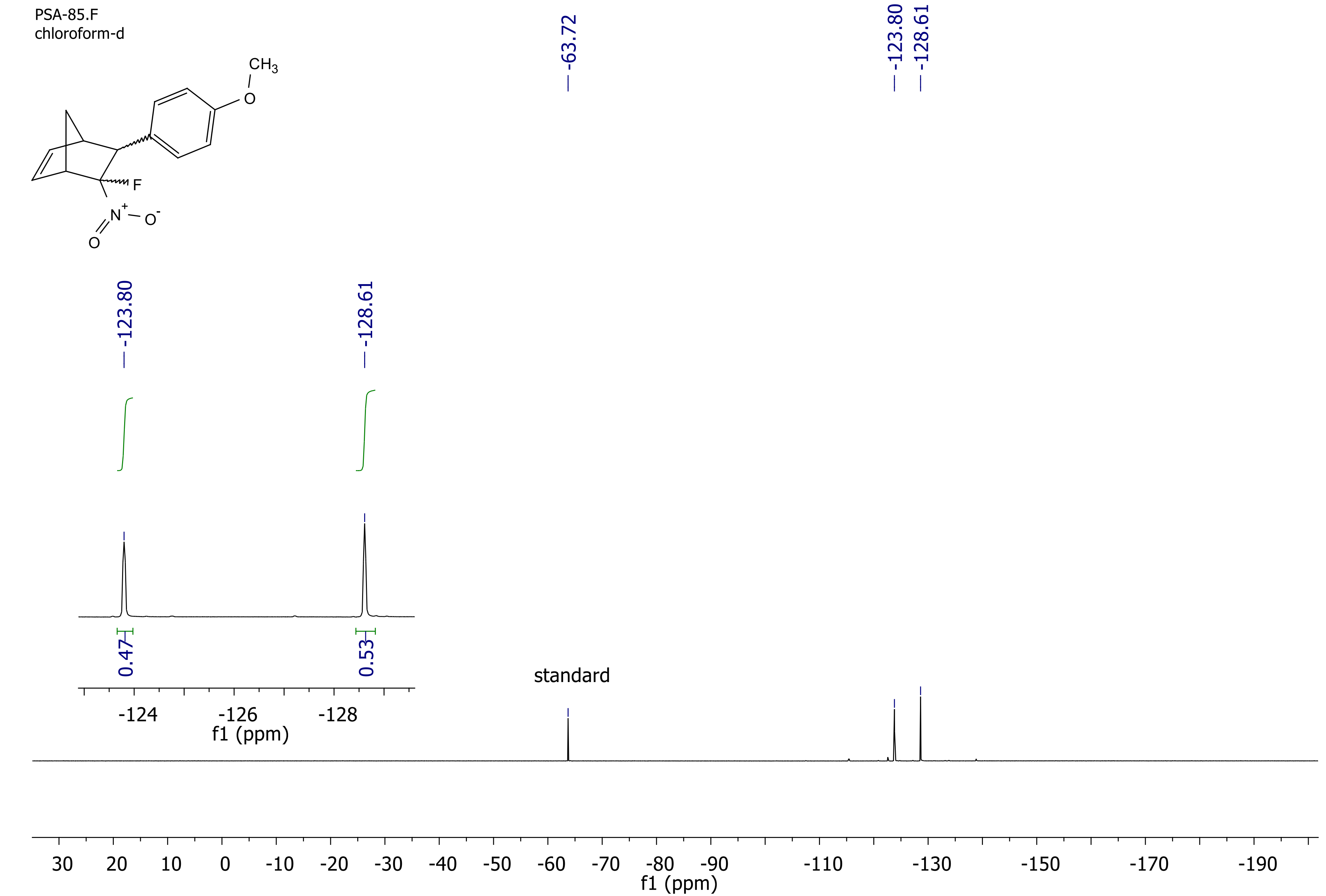

^{19}F NMR spectrum of 5-fluoro-6-(4-methoxyphenyl)-5-nitrobicyclo[2.2.1]hept-2-ene (**2g**)

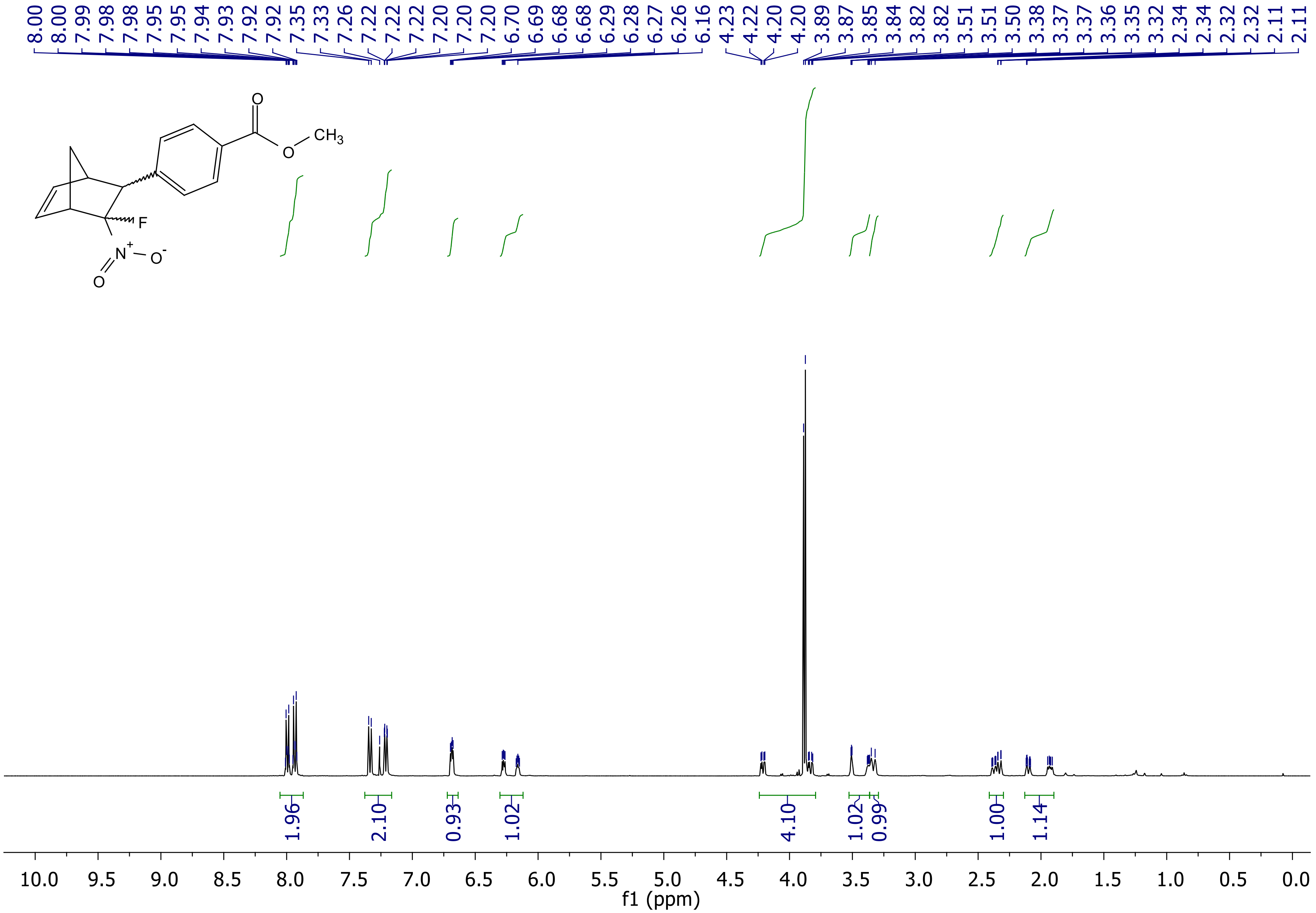

^{1}H NMR spectrum of methyl 4-(3-fluoro-3-nitrobicyclo[2.2.1]hept-5-en-2-yl)benzoate (**2h**)

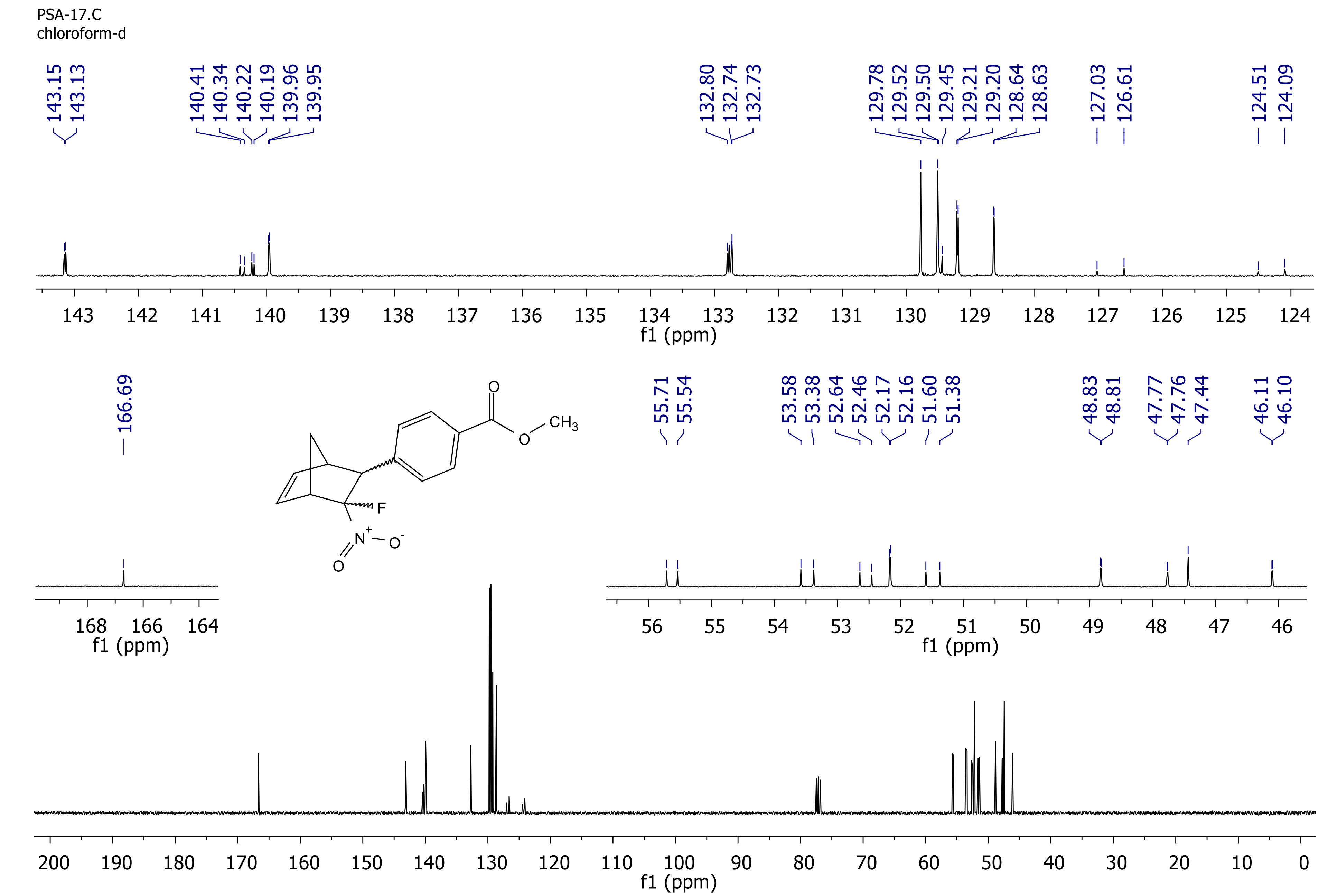

^{13}C NMR spectrum of methyl 4-(3-fluoro-3-nitrobicyclo[2.2.1]hept-5-en-2-yl)benzoate (**2h**)

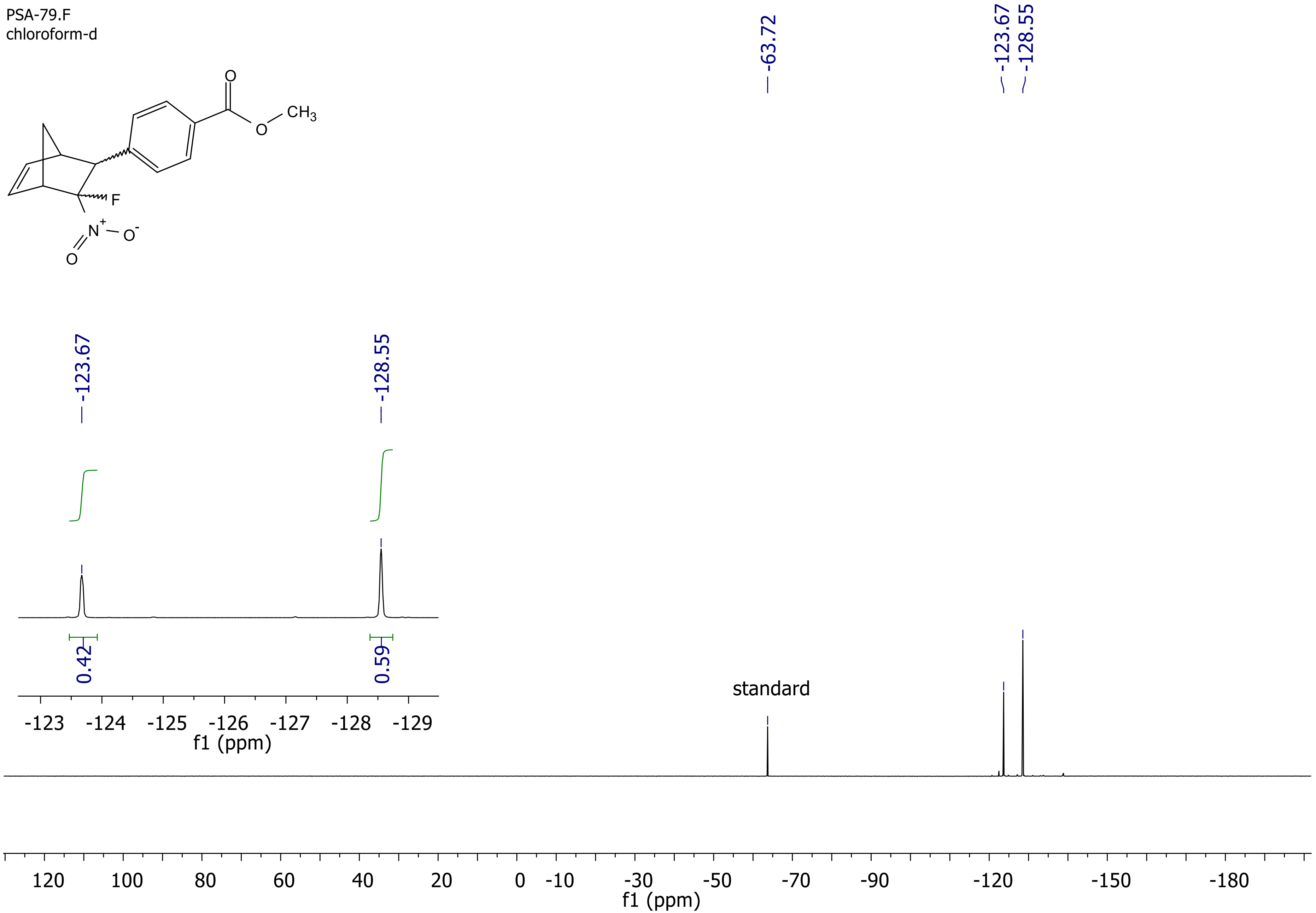

^{19}F NMR spectrum of methyl 4-(3-fluoro-3-nitrobicyclo[2.2.1]hept-5-en-2-yl)benzoate (**2h**)

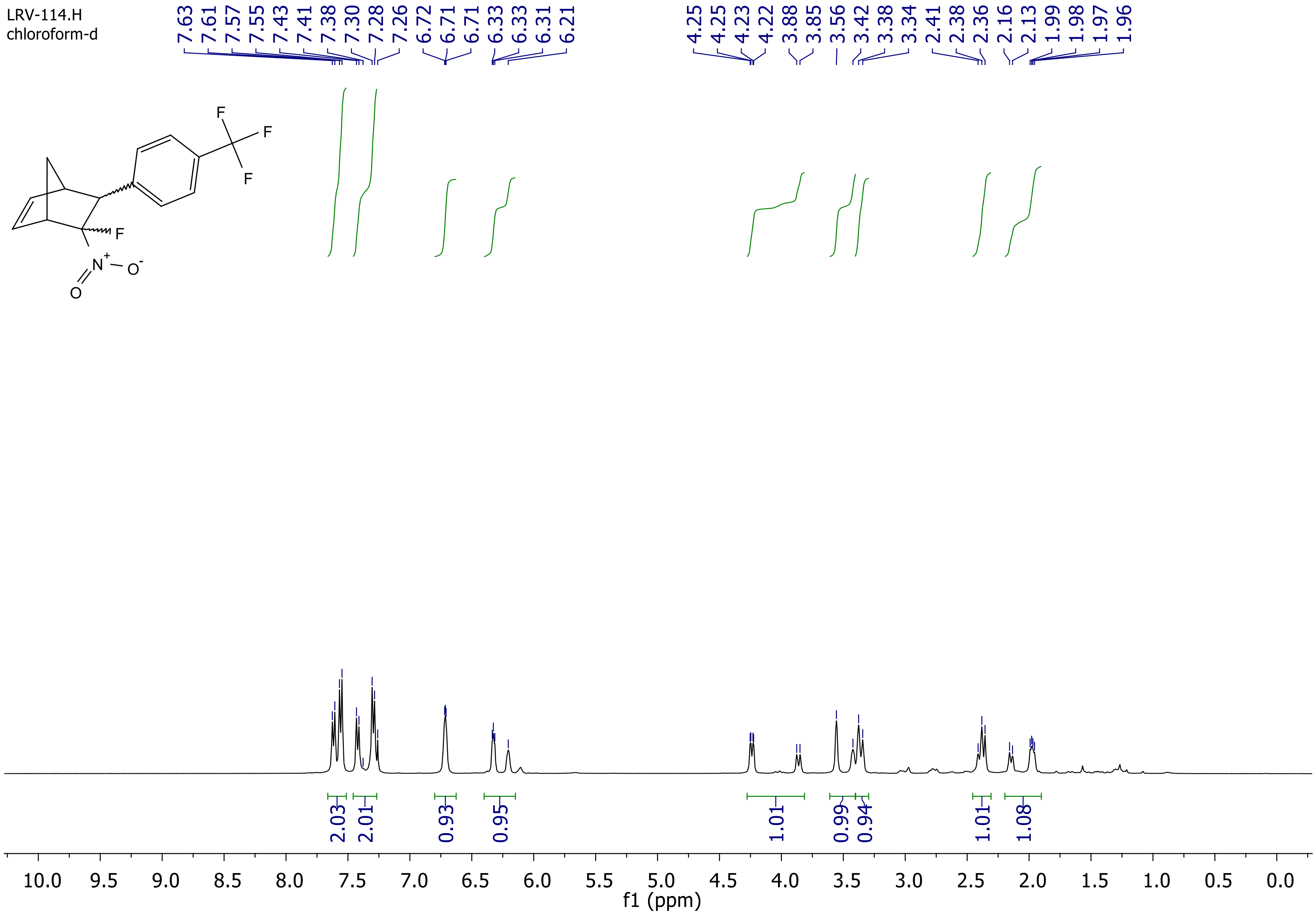

^{1}H NMR spectrum of 5-fluoro-5-nitro-6-(4-(trifluoromethyl)phenyl)bicyclo[2.2.1]hept-2-ene (**2i**)

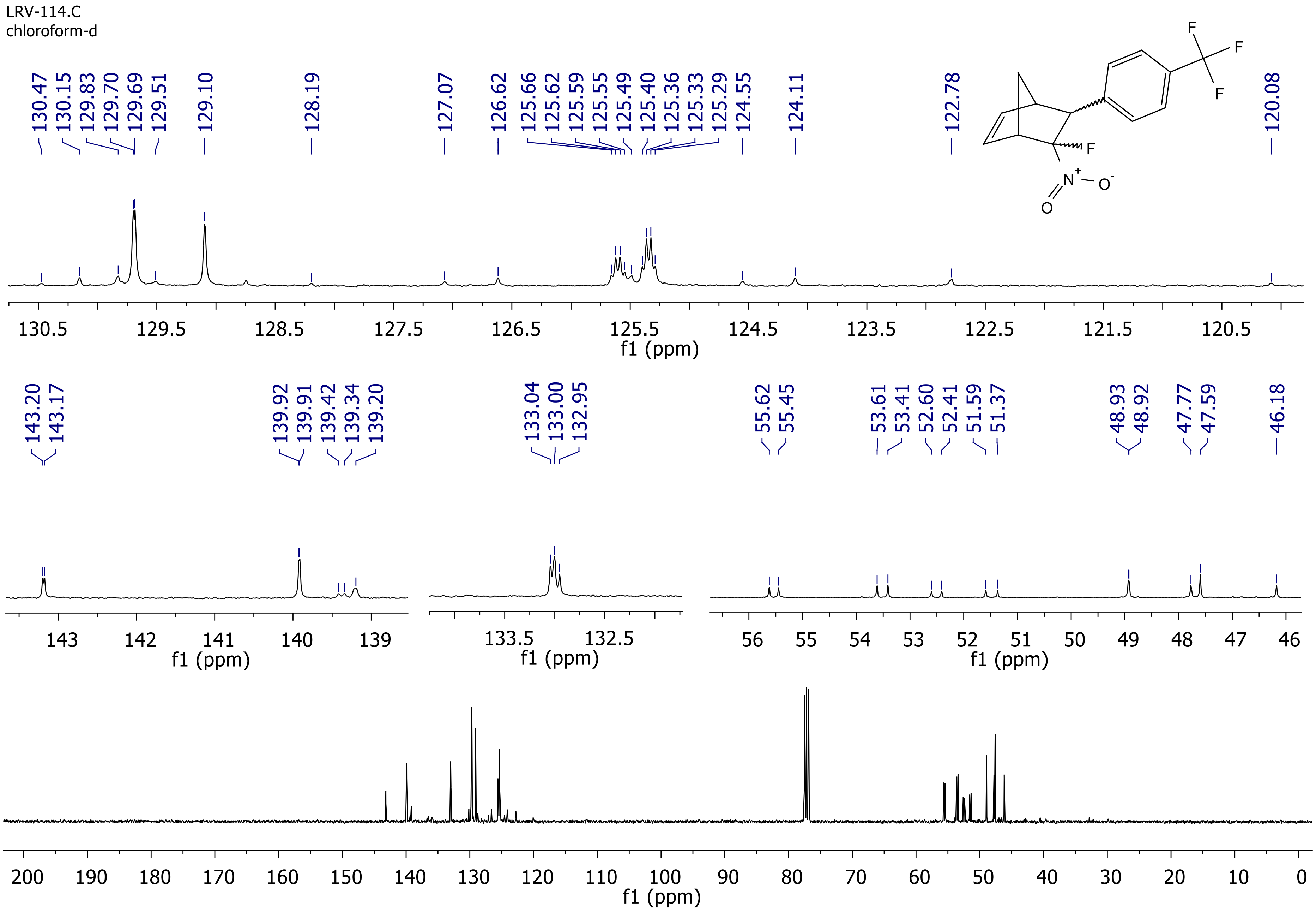

^{13}C NMR spectrum of 5-fluoro-5-nitro-6-(4-(trifluoromethyl)phenyl)bicyclo[2.2.1]hept-2-ene (**2i**)

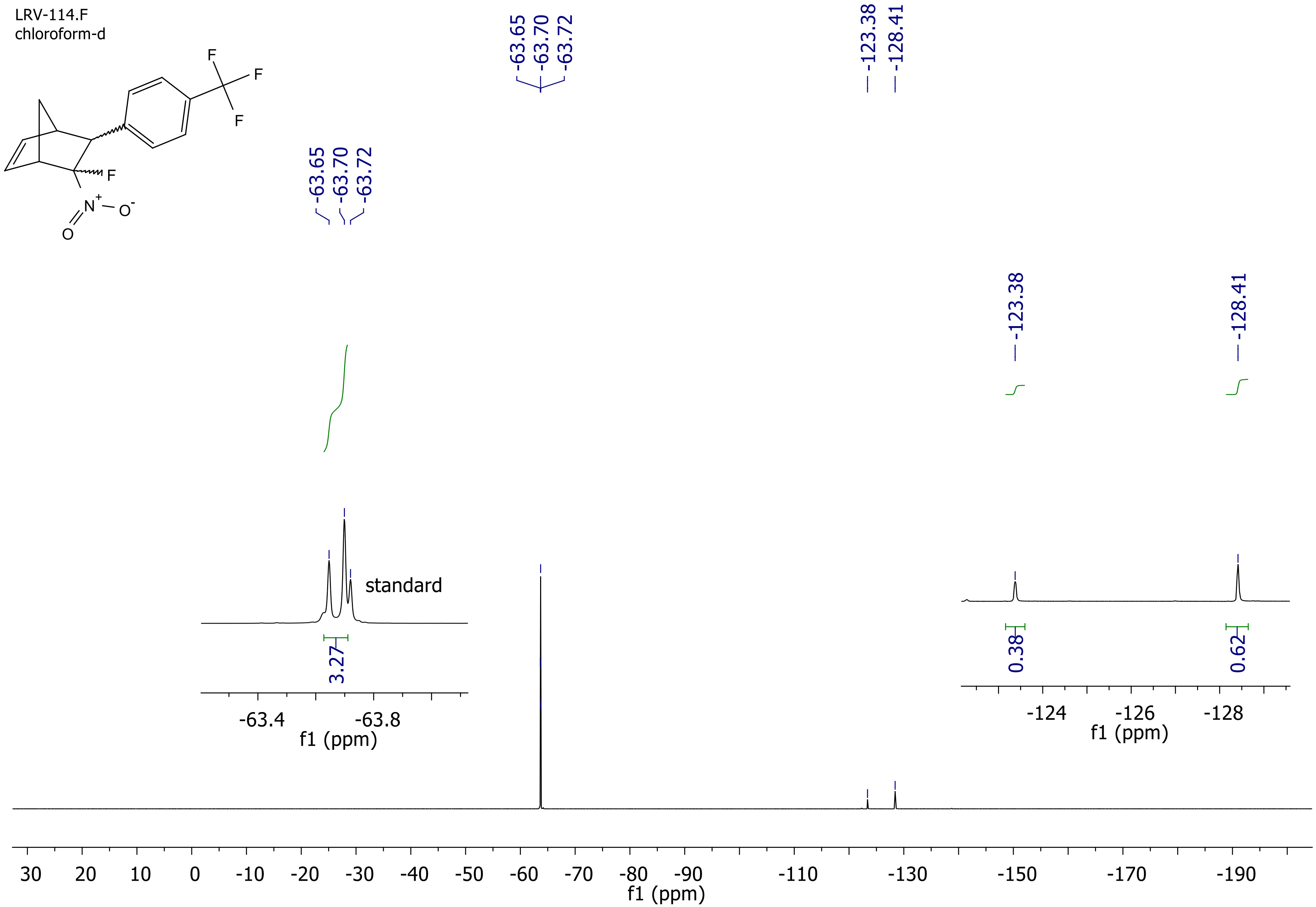

^{19}F NMR spectrum of 5-fluoro-5-nitro-6-(4-(trifluoromethyl)phenyl)bicyclo[2.2.1]hept-2-ene (**2i**)

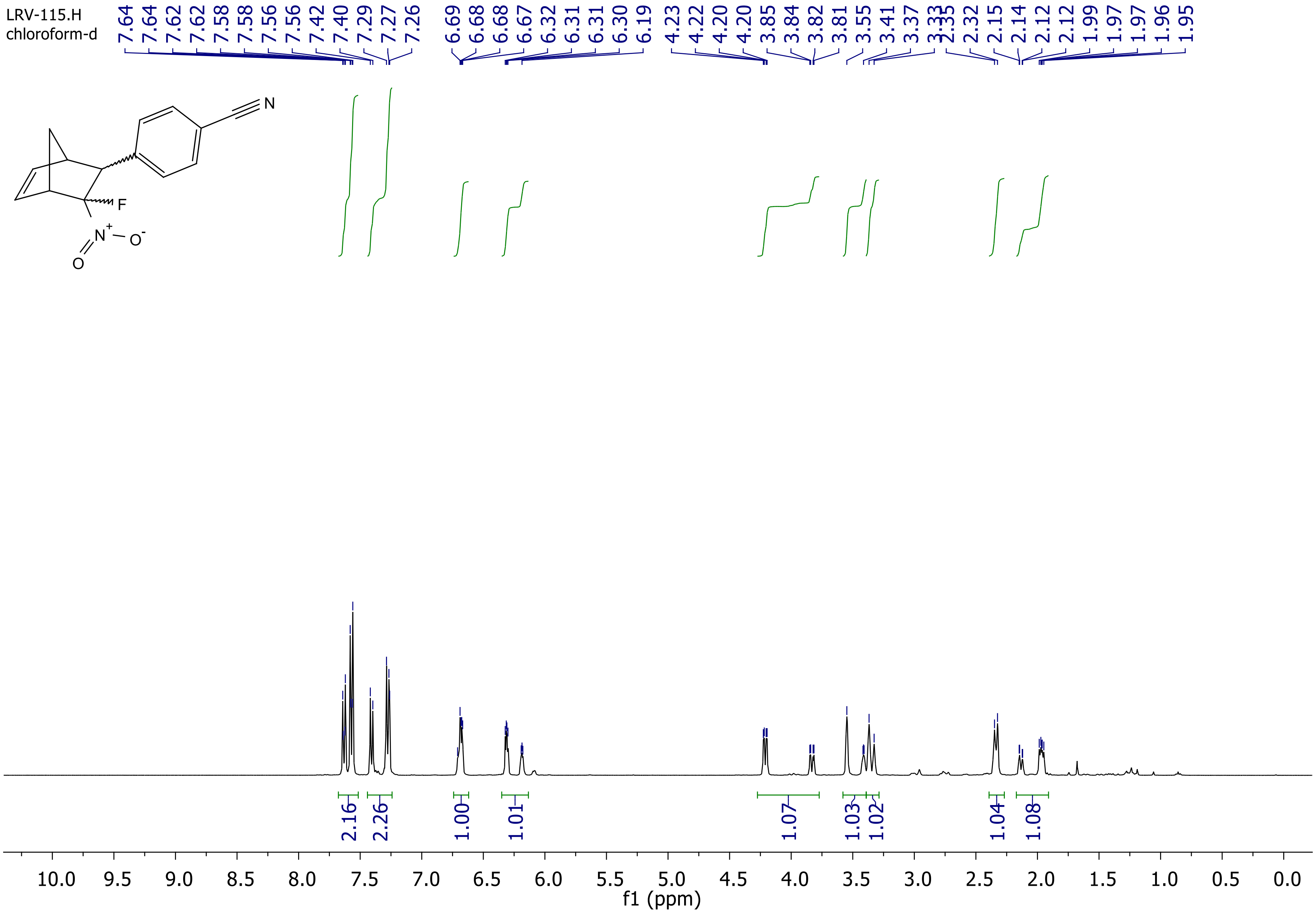

^{1}H NMR spectrum of 4-(3-fluoro-3-nitrobicyclo[2.2.1]hept-5-en-2-yl)benzonitrile (**2j**)

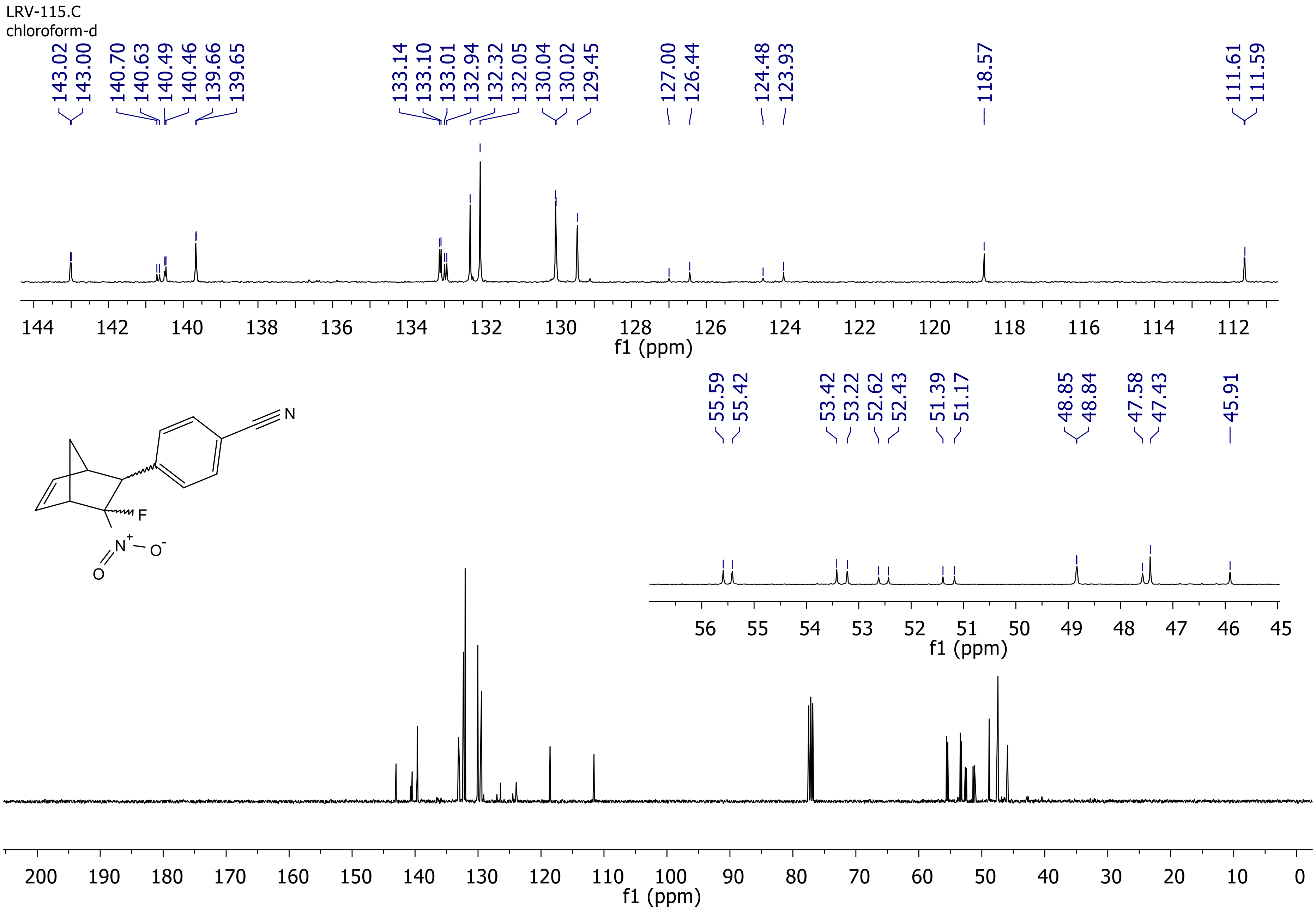

^{13}C NMR spectrum of 4-(3-fluoro-3-nitrobicyclo[2.2.1]hept-5-en-2-yl)benzonitrile (**2j**)

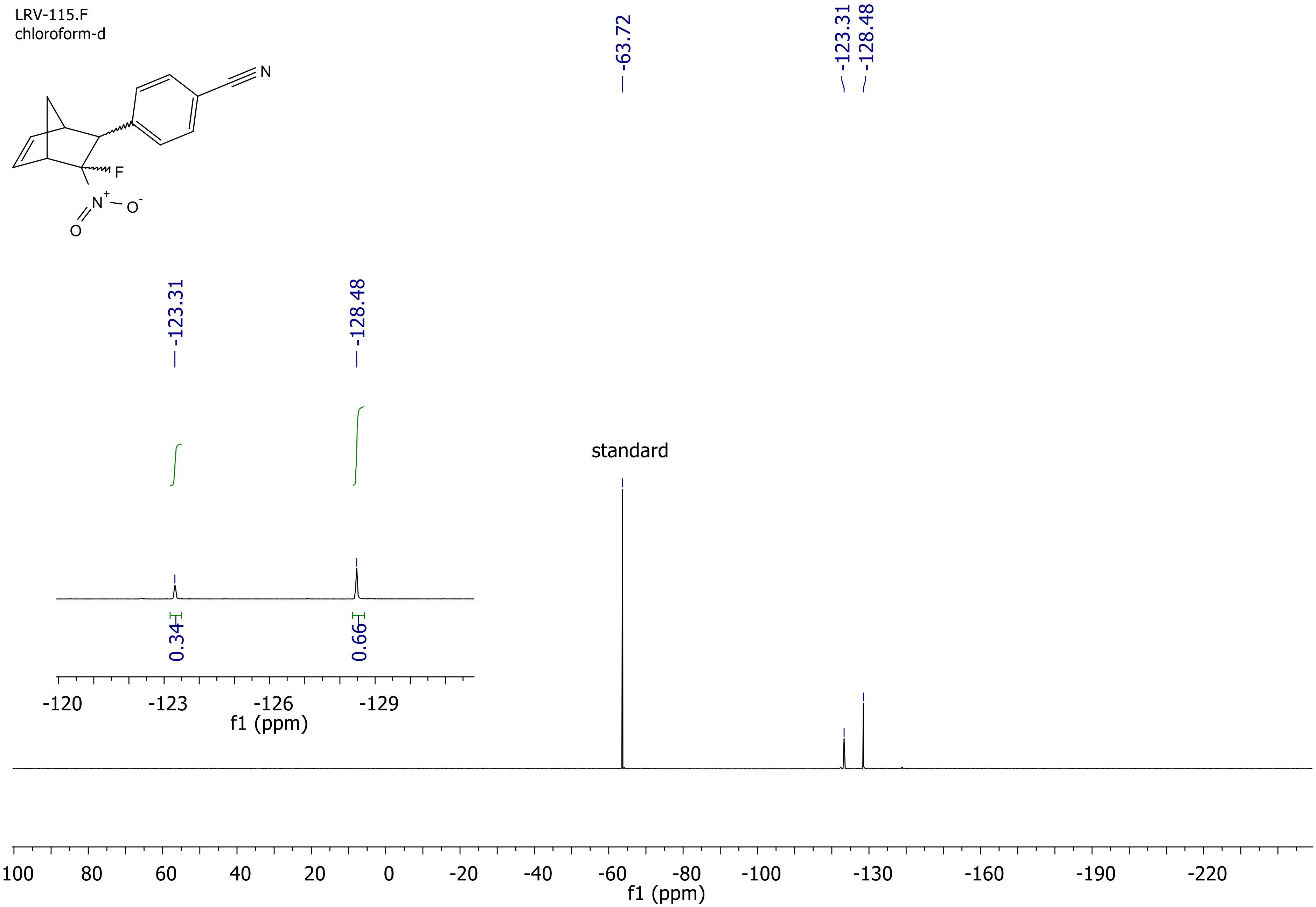

^{19}F NMR spectrum of 4-(3-fluoro-3-nitrobicyclo[2.2.1]hept-5-en-2-yl)benzonitrile (**2j**)

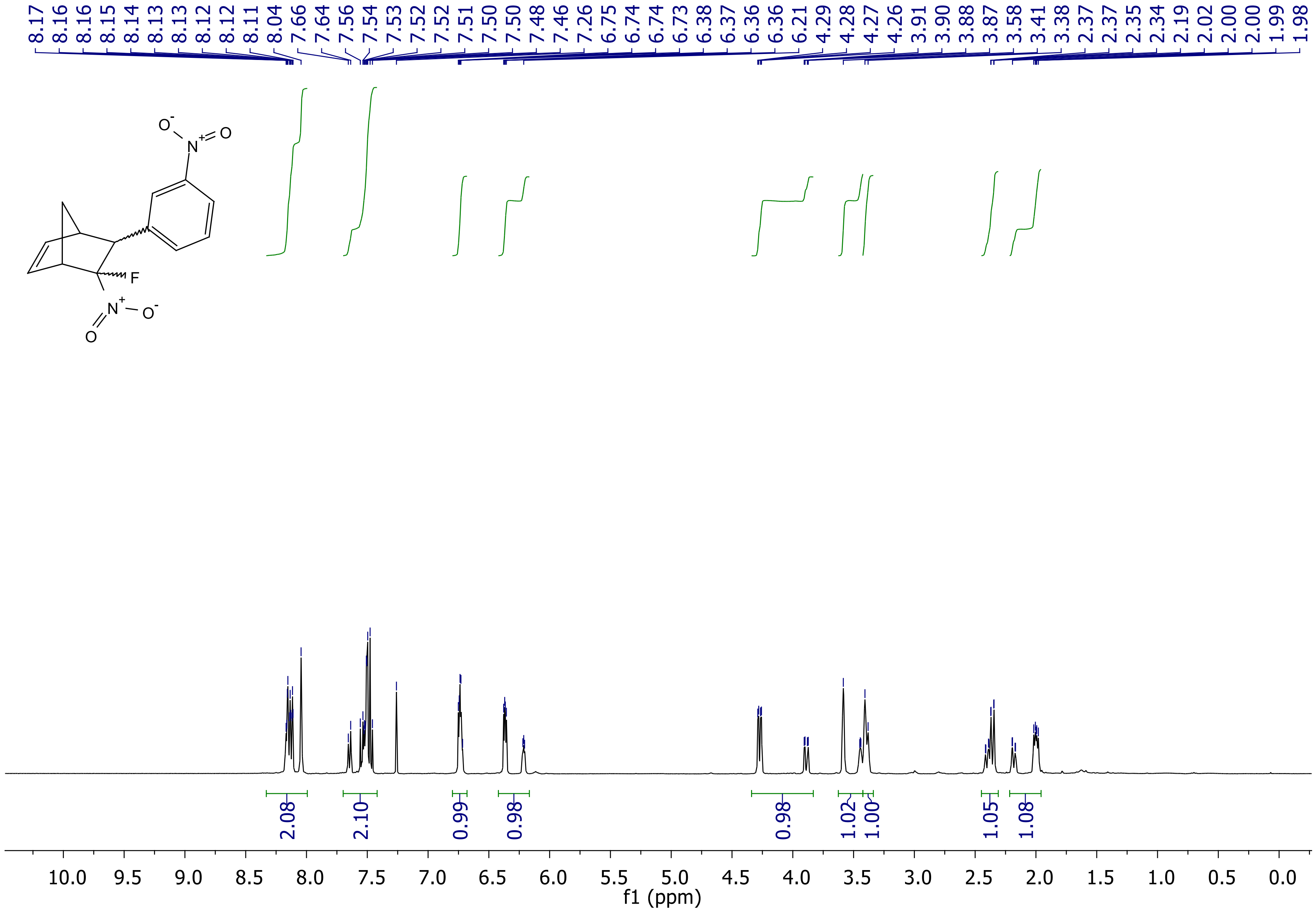

^{1}H NMR spectrum of 5-fluoro-5-nitro-6-(3-nitrophenyl)bicyclo[2.2.1]hept-2-ene (**2k**)

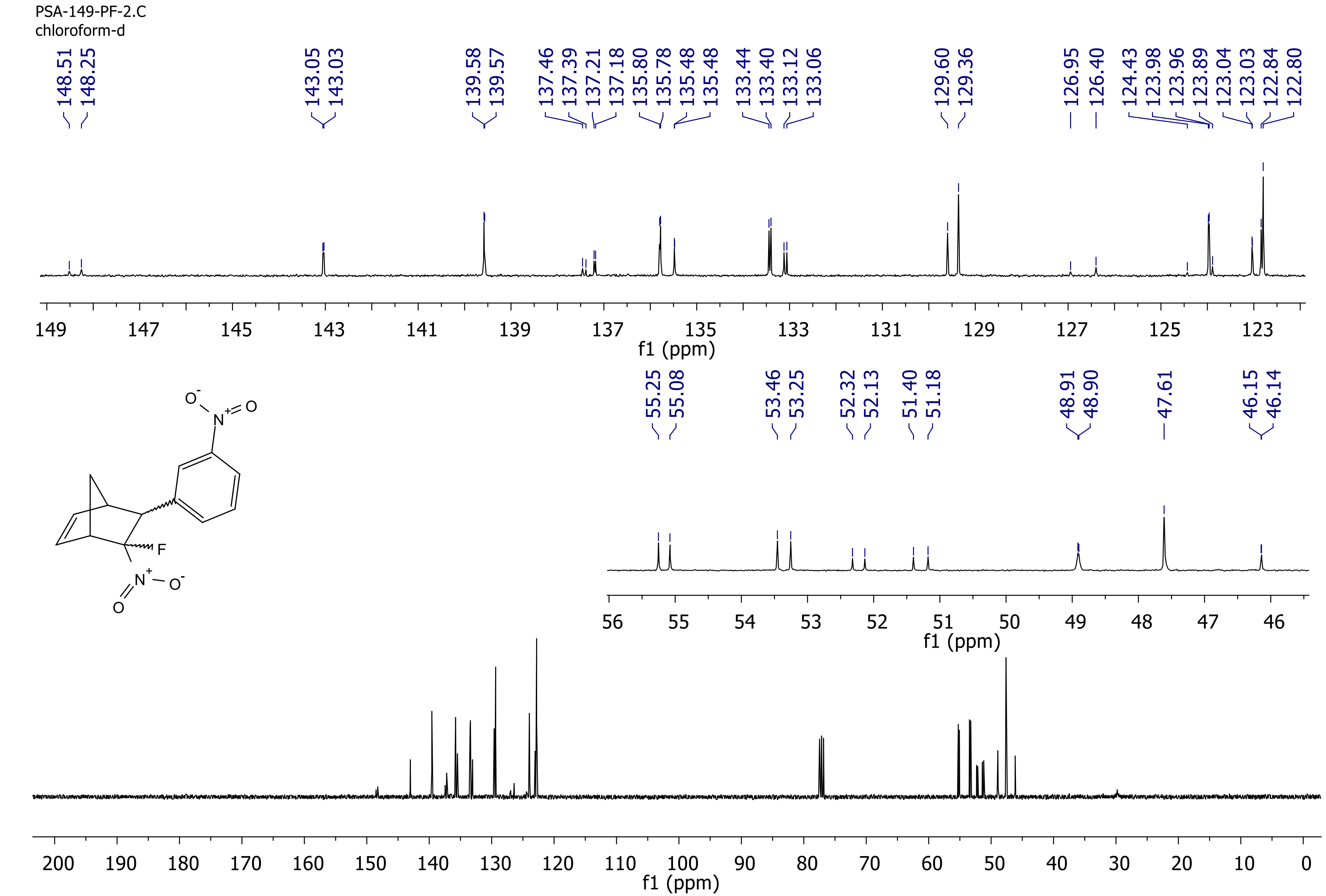

^{13}C NMR spectrum of 5-fluoro-5-nitro-6-(3-nitrophenyl)bicyclo[2.2.1]hept-2-ene (**2k**)

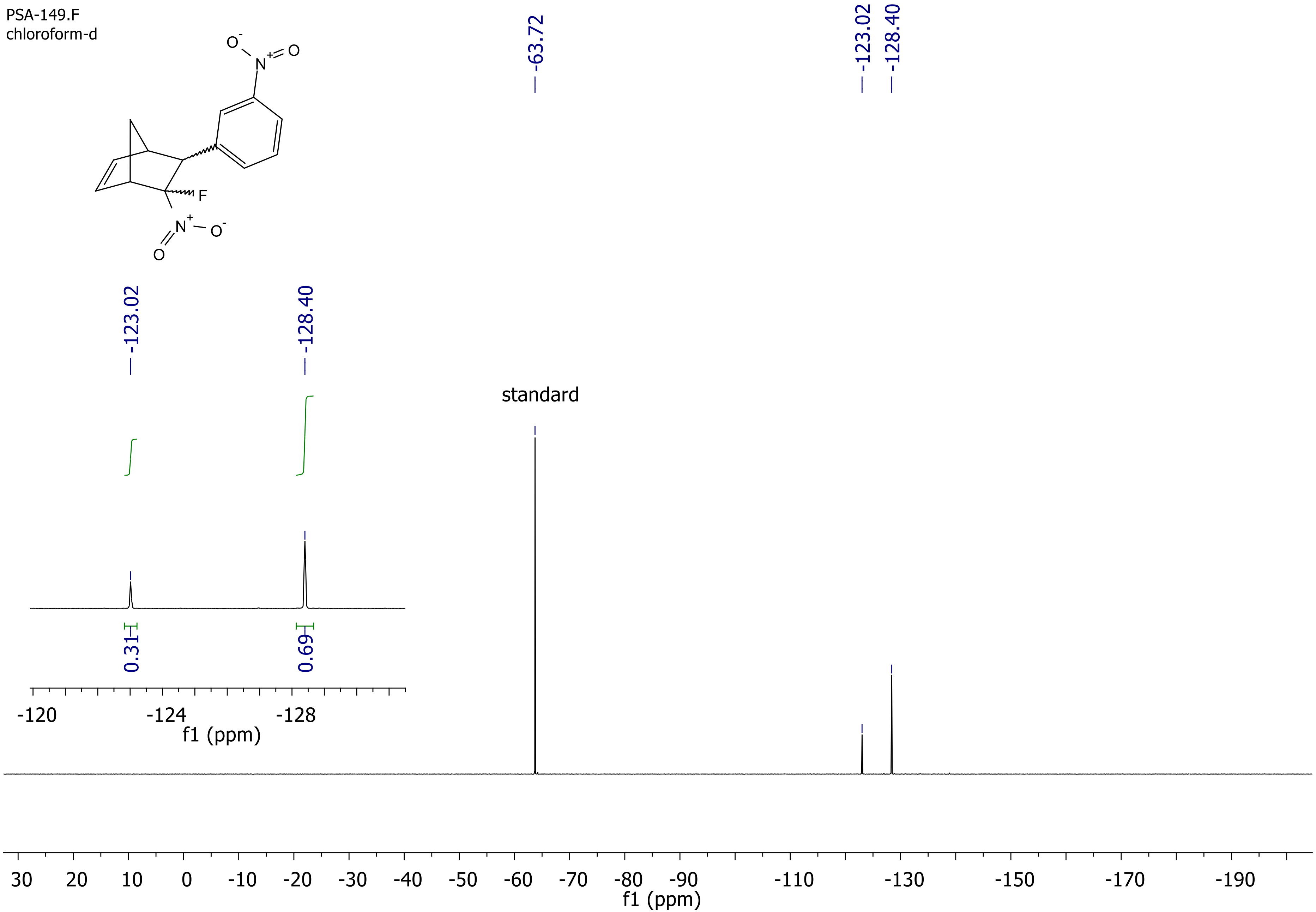

^{19}F NMR spectrum of 5-fluoro-5-nitro-6-(3-nitrophenyl)bicyclo[2.2.1]hept-2-ene (**2k**)

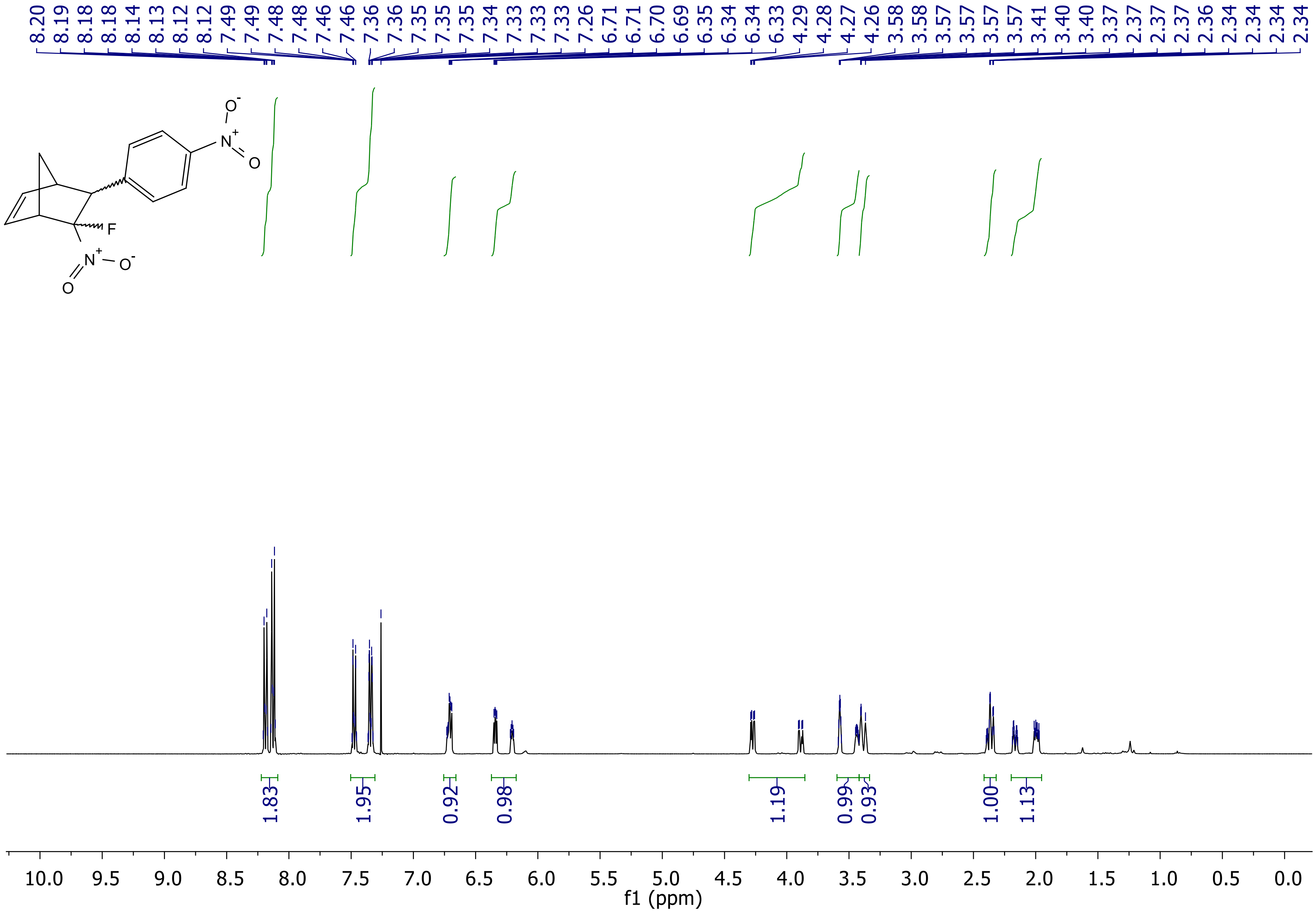

^{1}H NMR spectrum of 5-fluoro-5-nitro-6-(4-nitrophenyl)bicyclo[2.2.1]hept-2-ene (**2l**)

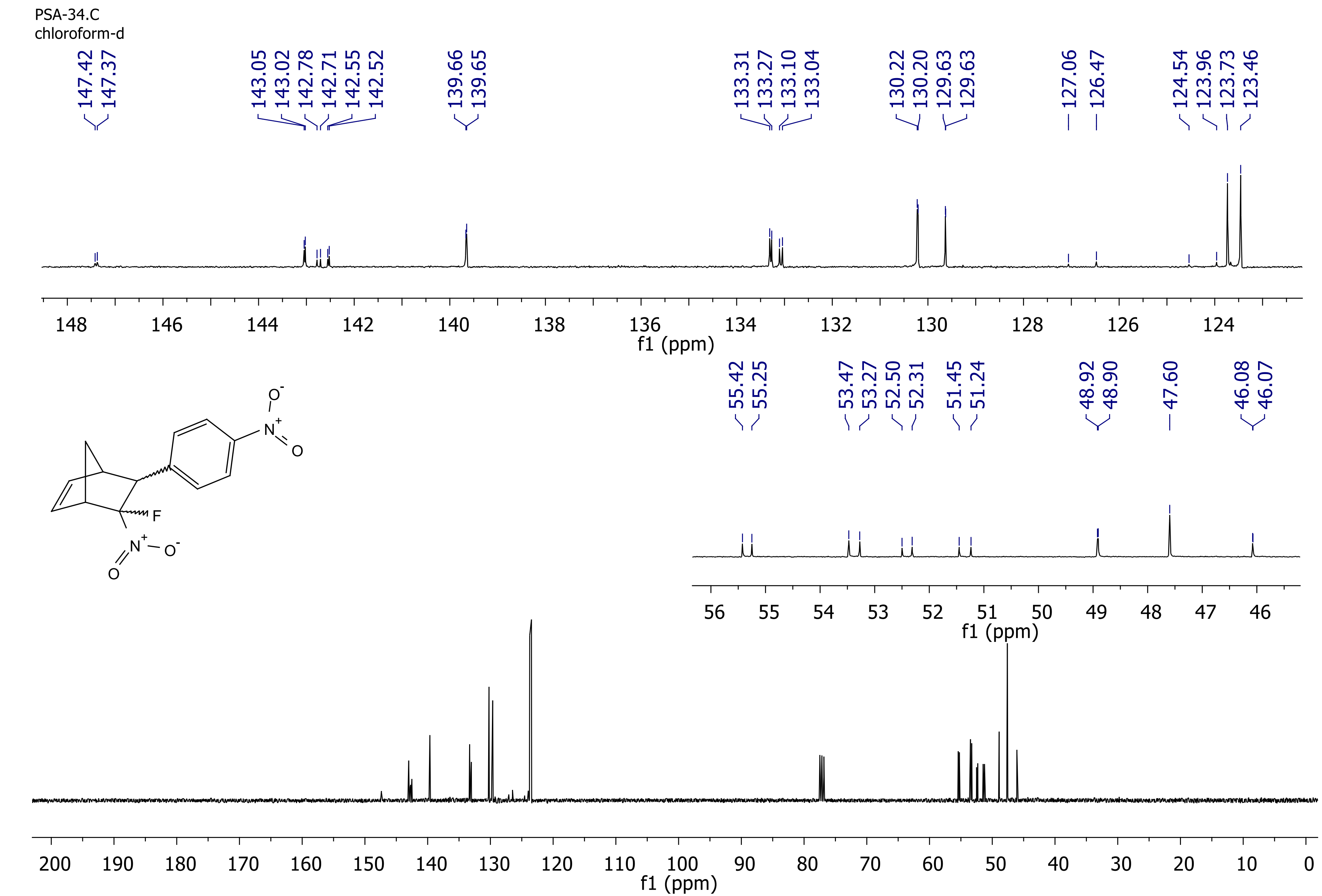

^{13}C NMR spectrum of 5-fluoro-5-nitro-6-(4-nitrophenyl)bicyclo[2.2.1]hept-2-ene (**2l**)

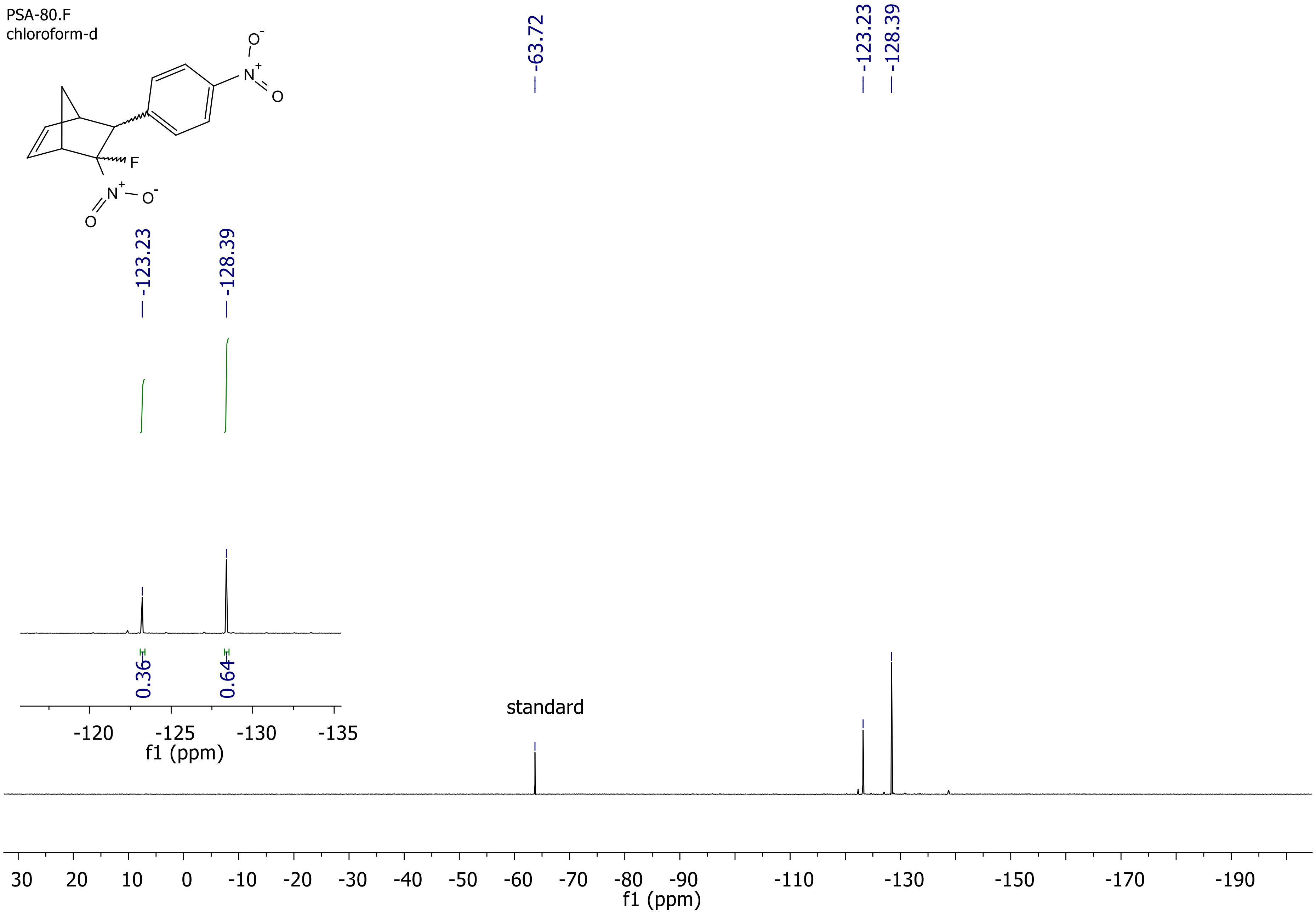

^{19}F NMR spectrum of 5-fluoro-5-nitro-6-(4-nitrophenyl)bicyclo[2.2.1]hept-2-ene (**2l**)

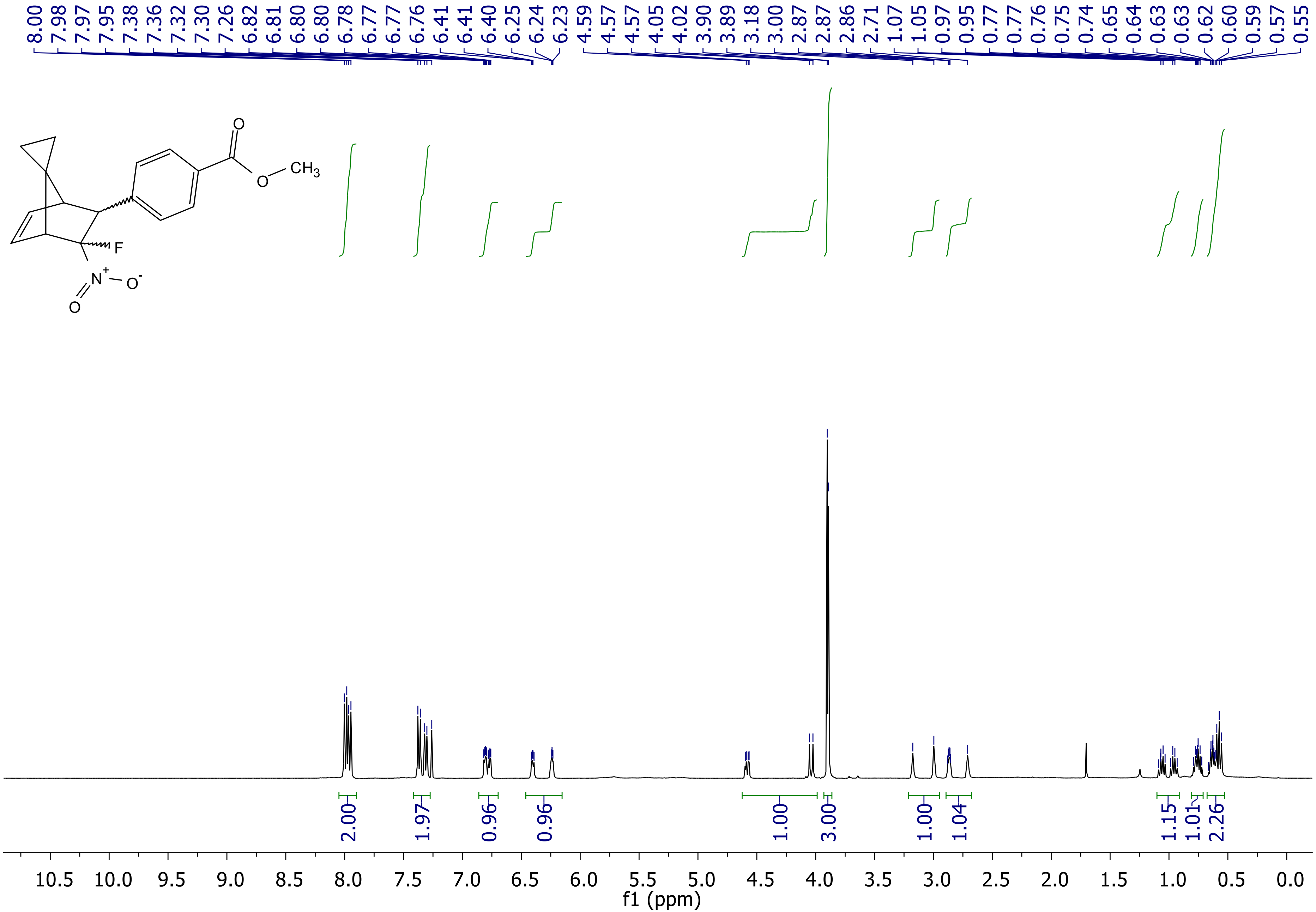

^{1}H NMR spectrum of methyl 4-(5-fluoro-5-nitrospiro[bicyclo[2.2.1]hept[2]ene-7,1'-cyclopropan]-6-yl)benzoate (**2m**)

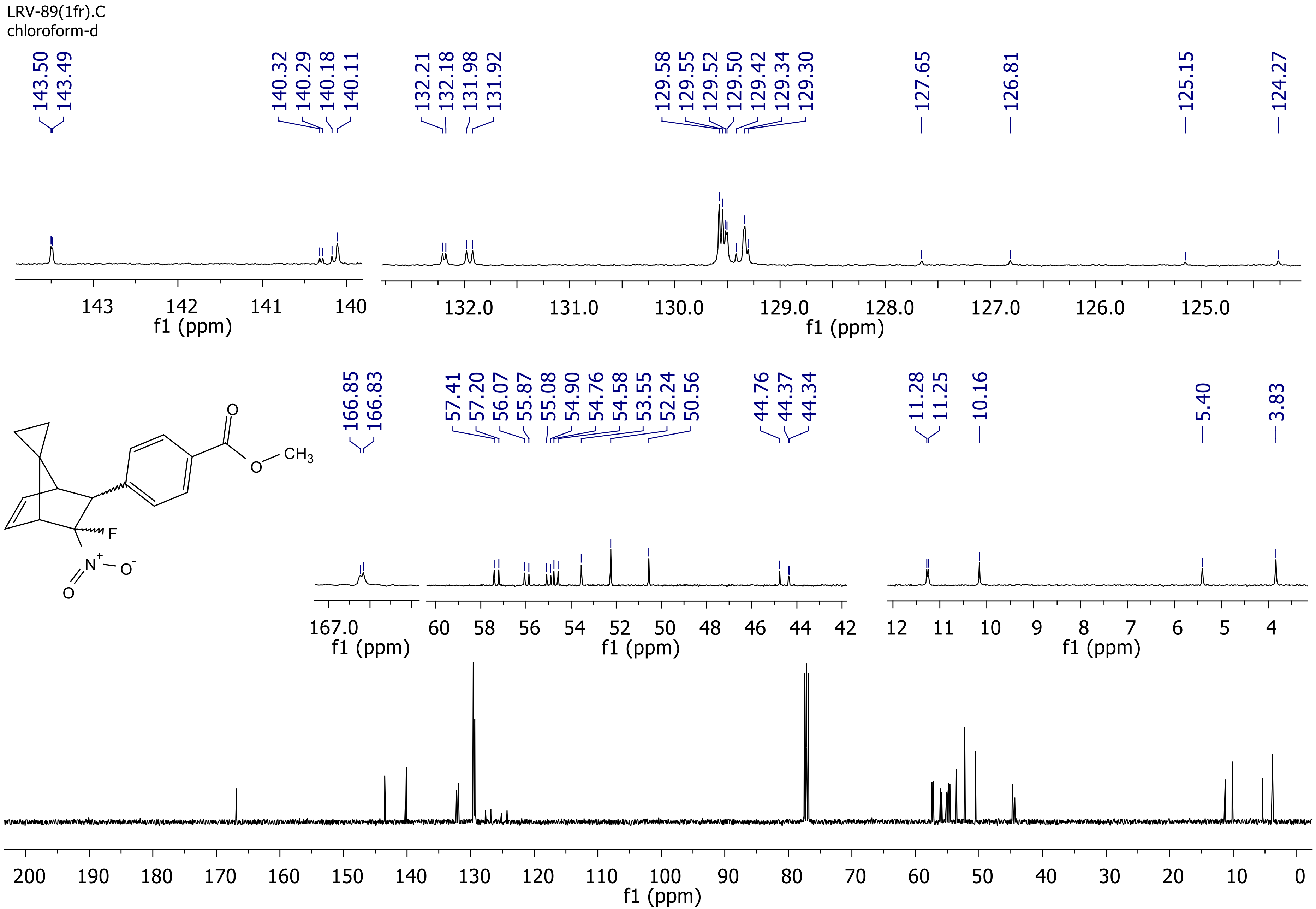

^{13}C NMR spectrum of methyl 4-(5-fluoro-5-nitrospiro[bicyclo[2.2.1]hept[2]ene-7,1'-cyclopropan]-6-yl)benzoate (**2m**)

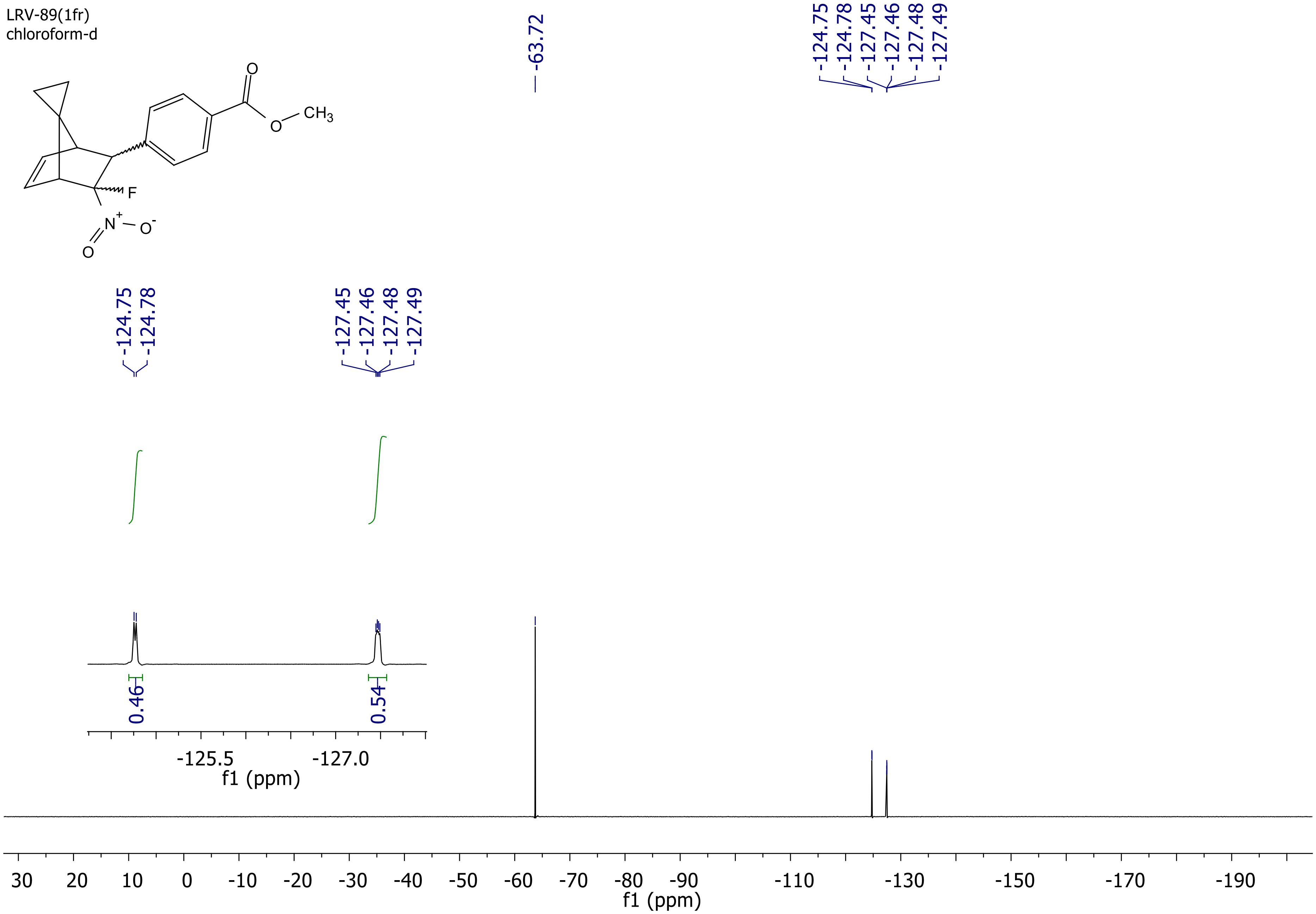

^{19}F NMR spectrum of methyl 4-(5-fluoro-5-nitrospiro[bicyclo[2.2.1]hept[2]ene-7,1'-cyclopropan]-6-yl)benzoate (**2m**)

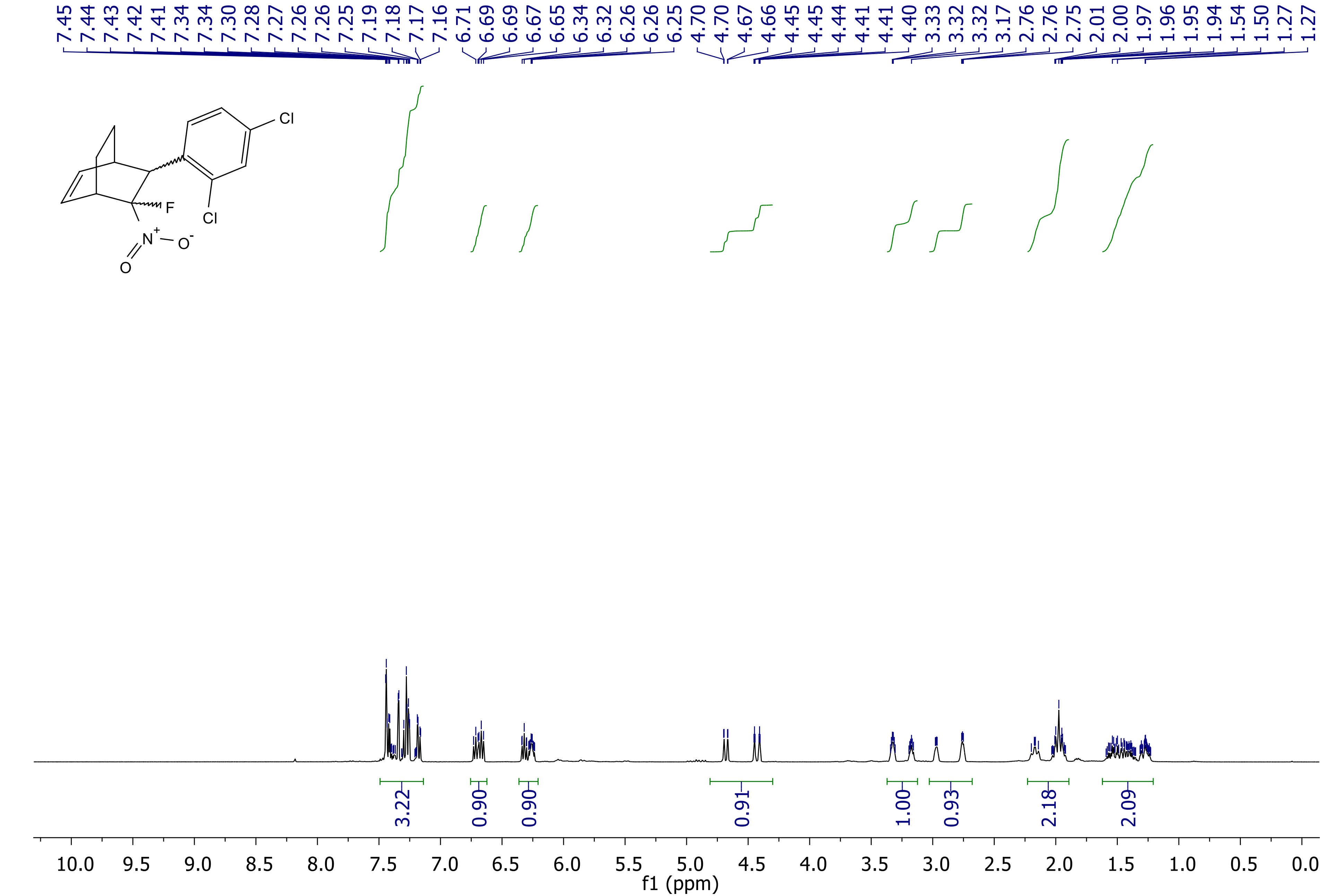

^{1}H NMR spectrum of 6-(2,4-dichlorophenyl)-5-fluoro-5-nitrobicyclo[2.2.2]oct-2-ene (**3a**)

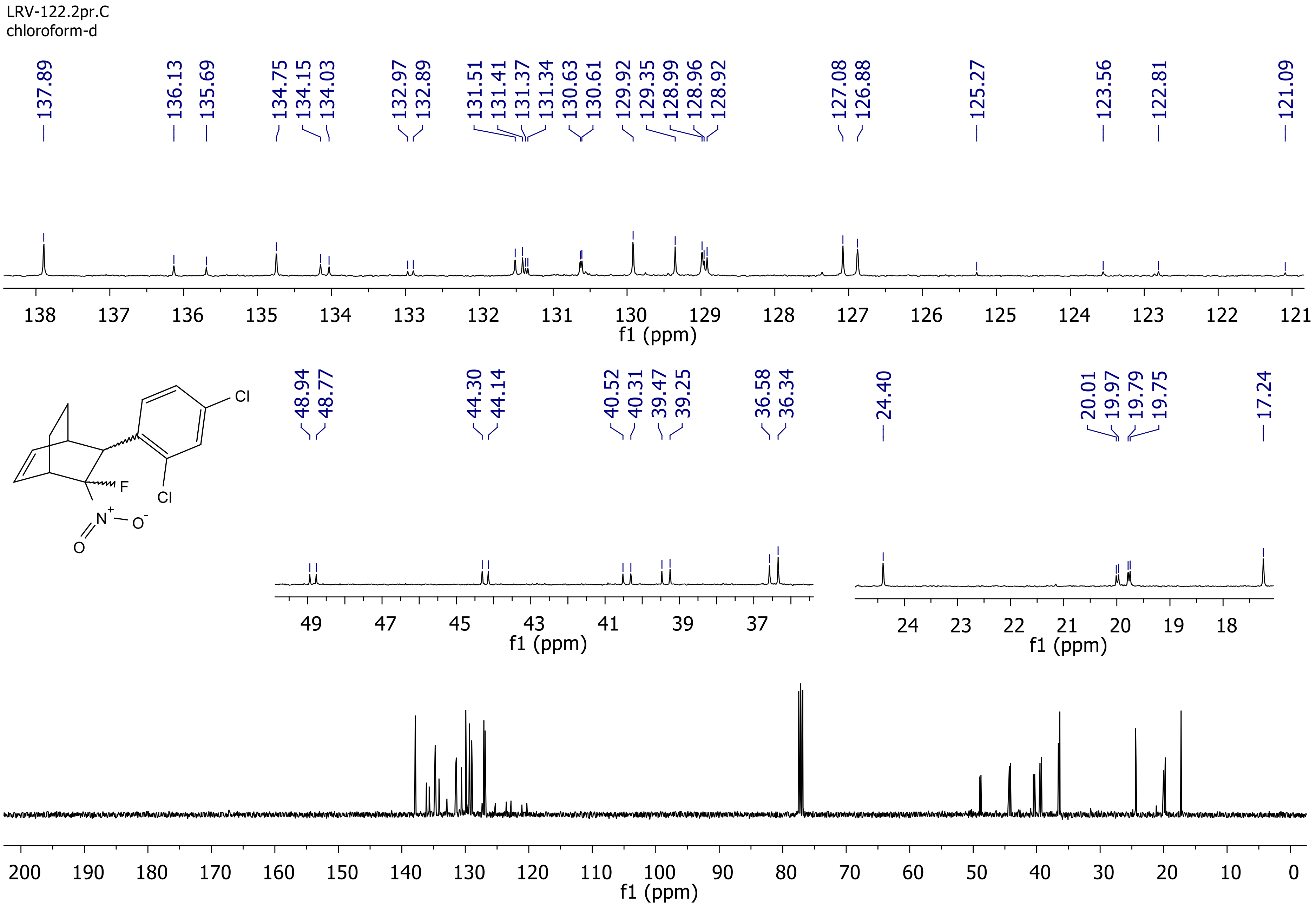

^{13}C NMR spectrum of 6-(2,4-dichlorophenyl)-5-fluoro-5-nitrobicyclo[2.2.2]oct-2-ene (**3a**)

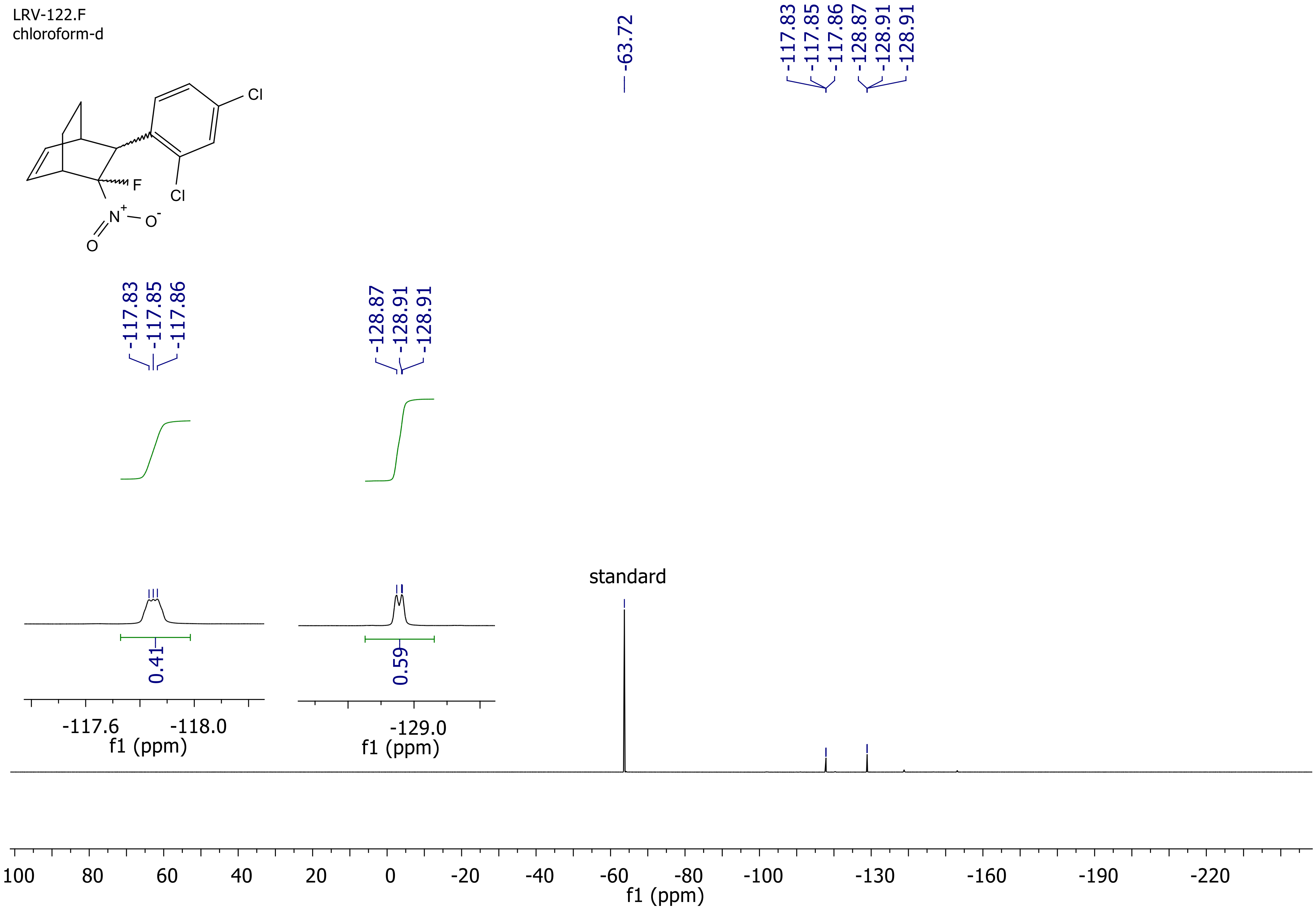

^{19}F NMR spectrum of 6-(2,4-dichlorophenyl)-5-fluoro-5-nitrobicyclo[2.2.2]oct-2-ene (**3a**)

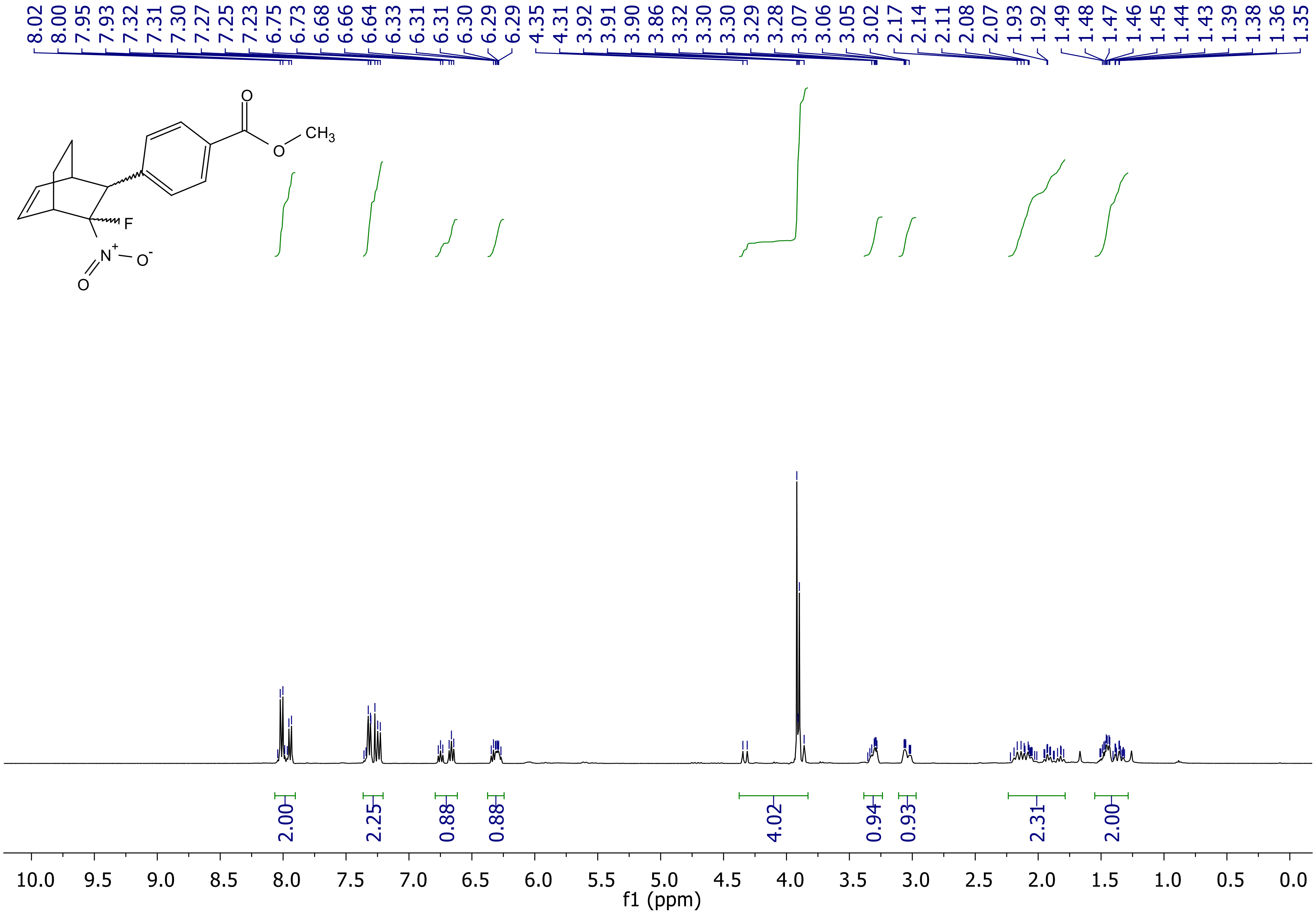

^{1}H NMR spectrum of methyl 4-(3-fluoro-3-nitrobicyclo[2.2.2]oct-5-en-2-yl)benzoate (**3b**)

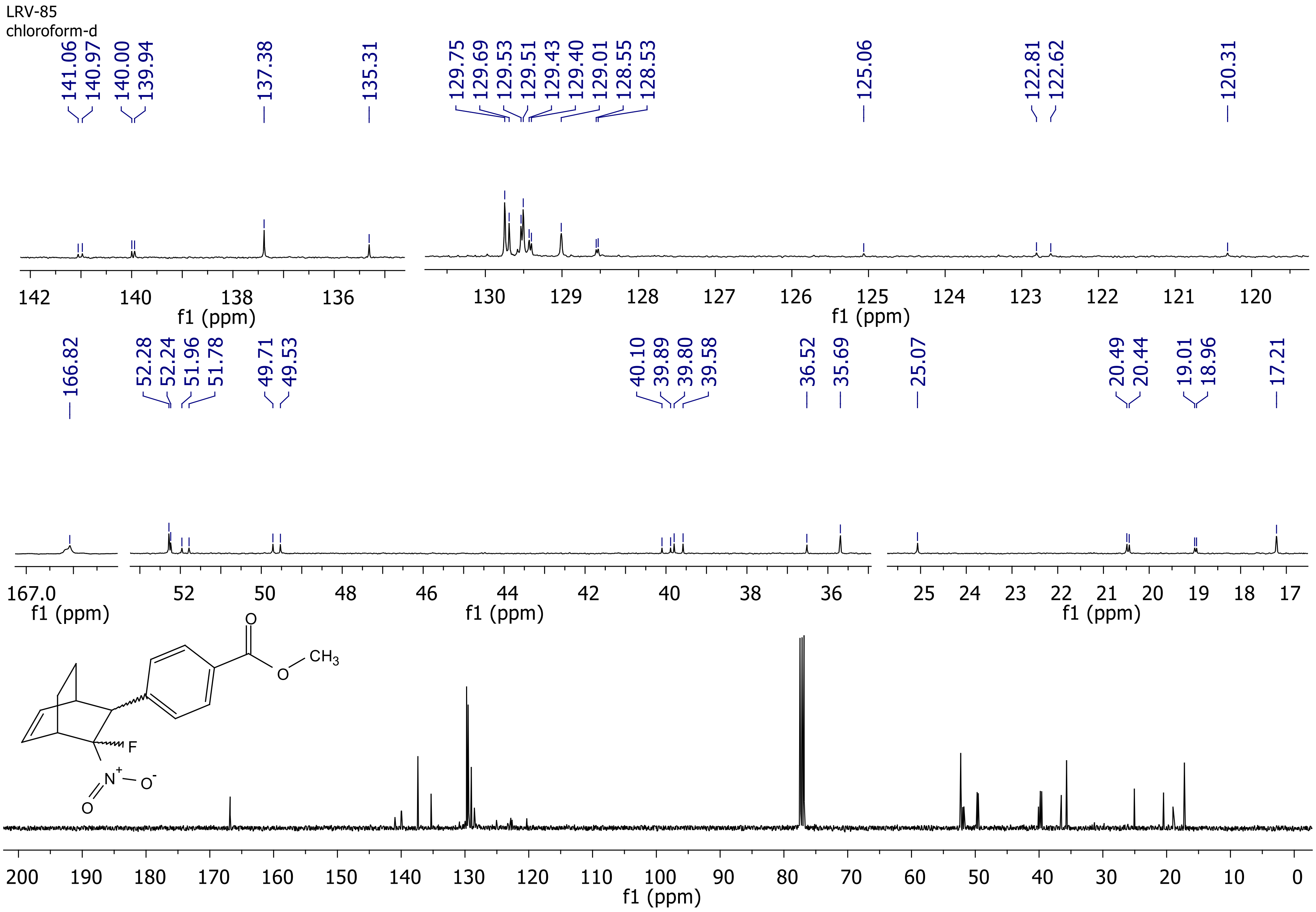

^{13}C NMR spectrum of methyl 4-(3-fluoro-3-nitrobicyclo[2.2.2]oct-5-en-2-yl)benzoate (**3b**)

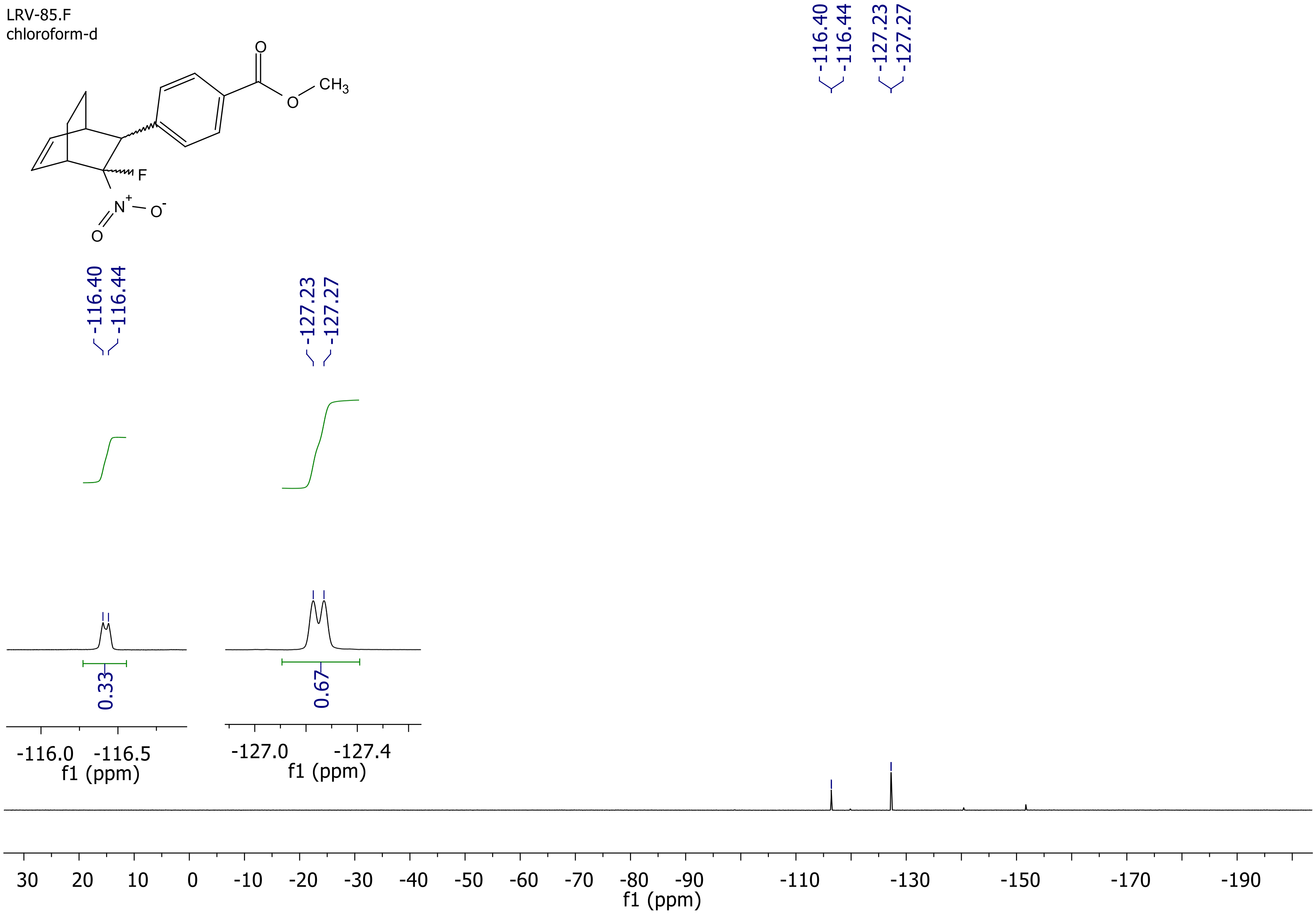

^{19}F NMR spectrum of methyl 4-(3-fluoro-3-nitrobicyclo[2.2.2]oct-5-en-2-yl)benzoate (**3b**)

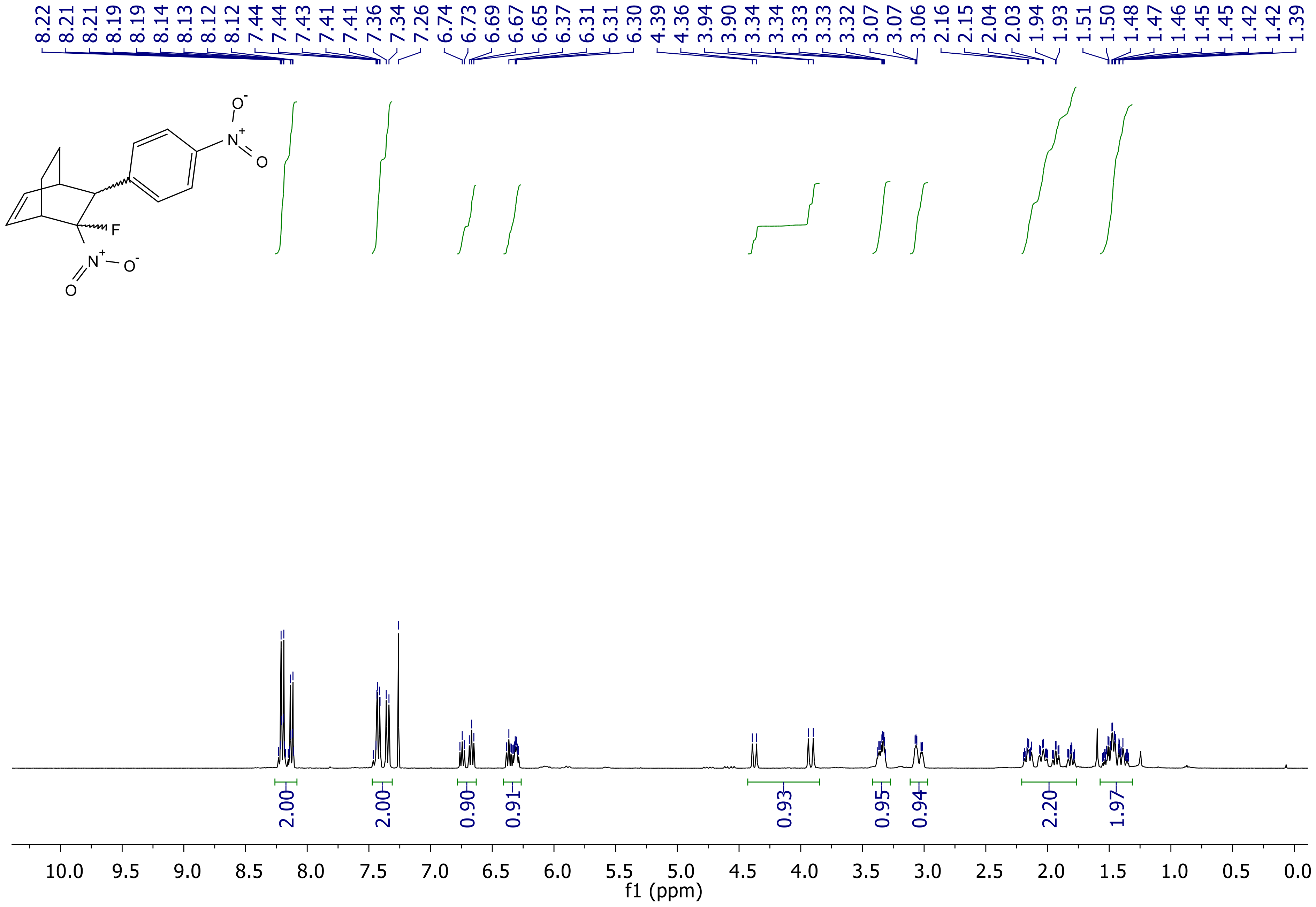

^{1}H NMR spectrum of 5-fluoro-5-nitro-6-(4-nitrophenyl)bicyclo[2.2.2]oct-2-ene (**3c**)

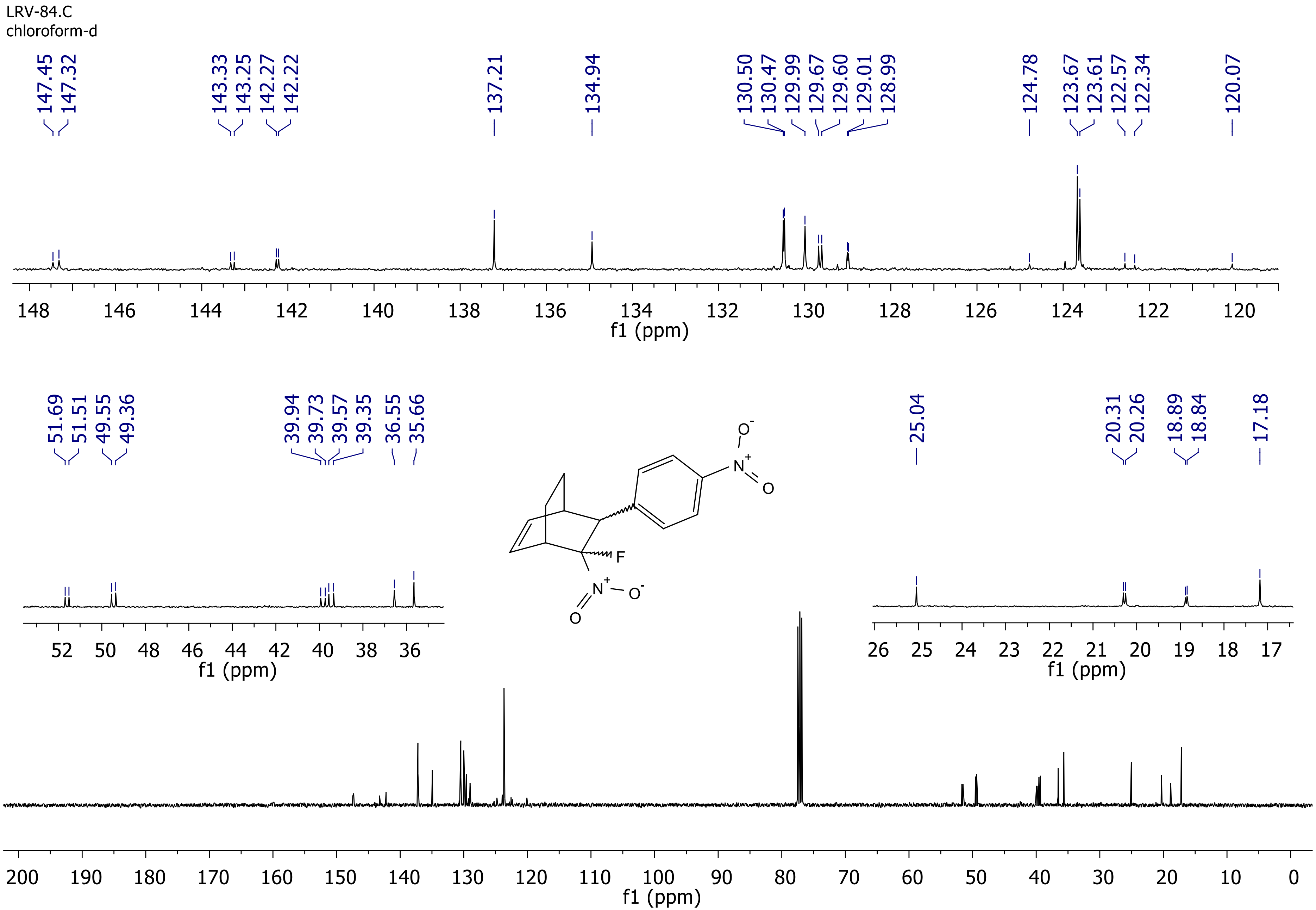

^{13}C NMR spectrum of 5-fluoro-5-nitro-6-(4-nitrophenyl)bicyclo[2.2.2]oct-2-ene (**3c**)

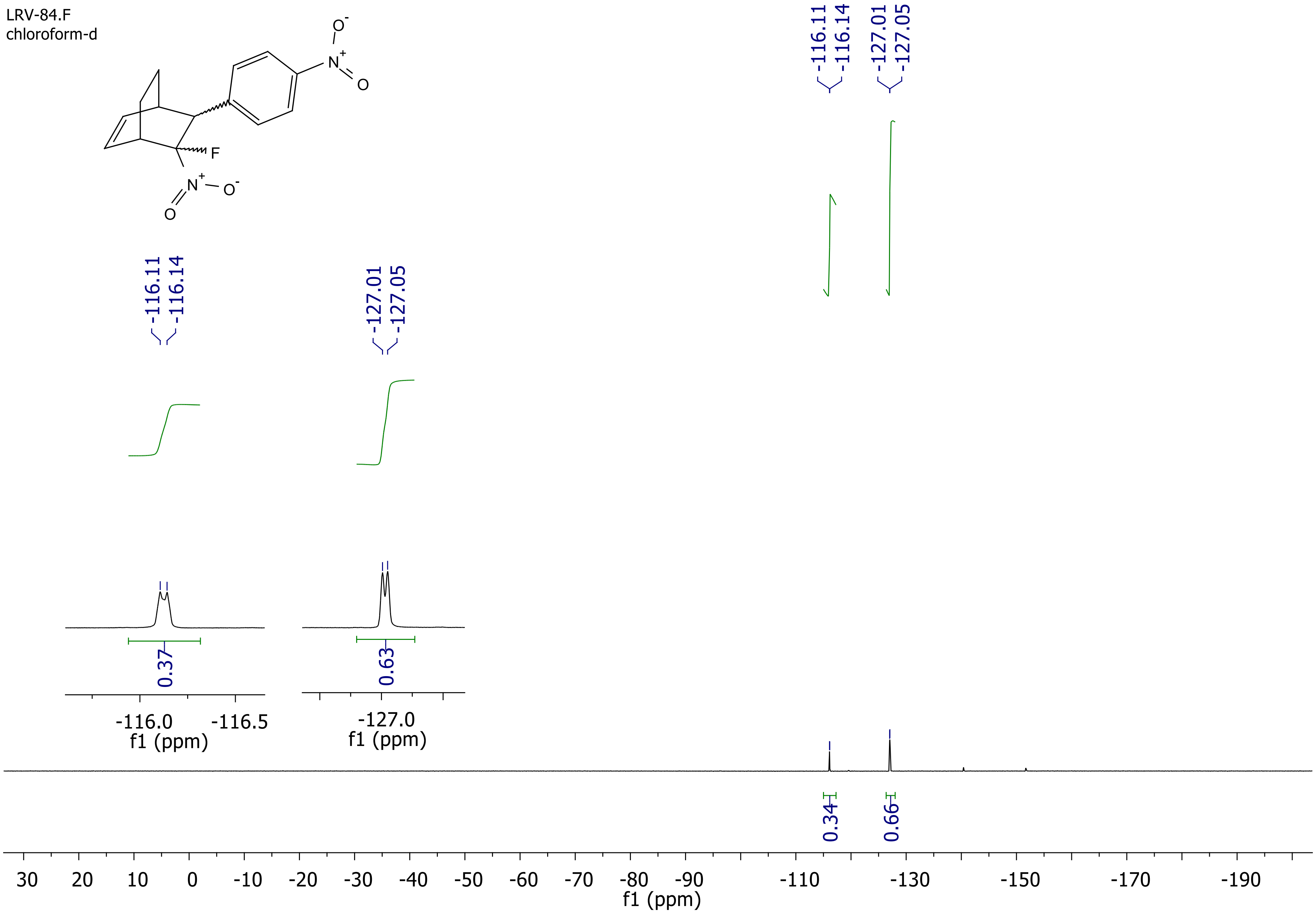

^{19}F NMR spectrum of 5-fluoro-5-nitro-6-(4-nitrophenyl)bicyclo[2.2.2]oct-2-ene (**3c**)

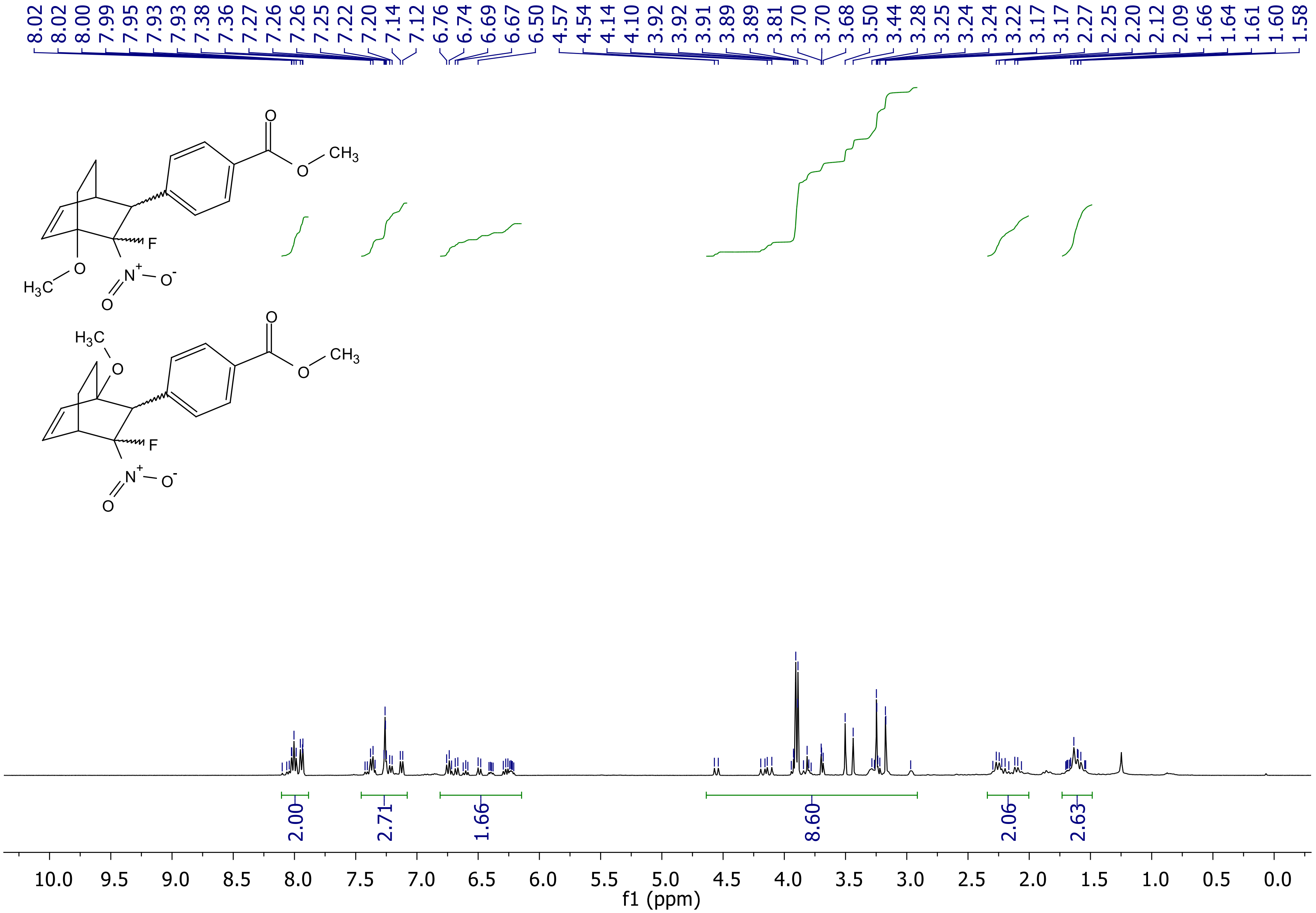

^{1}H NMR spectrum of methyl 4-(3-fluoro-4-methoxy-3-nitrobicyclo[2.2.2]oct-5-en-2-yl)benzoate and methyl 4-(3-fluoro-1-methoxy-3-nitrobicyclo[2.2.2]oct-5-en-2-yl)benzoate (**3d**)

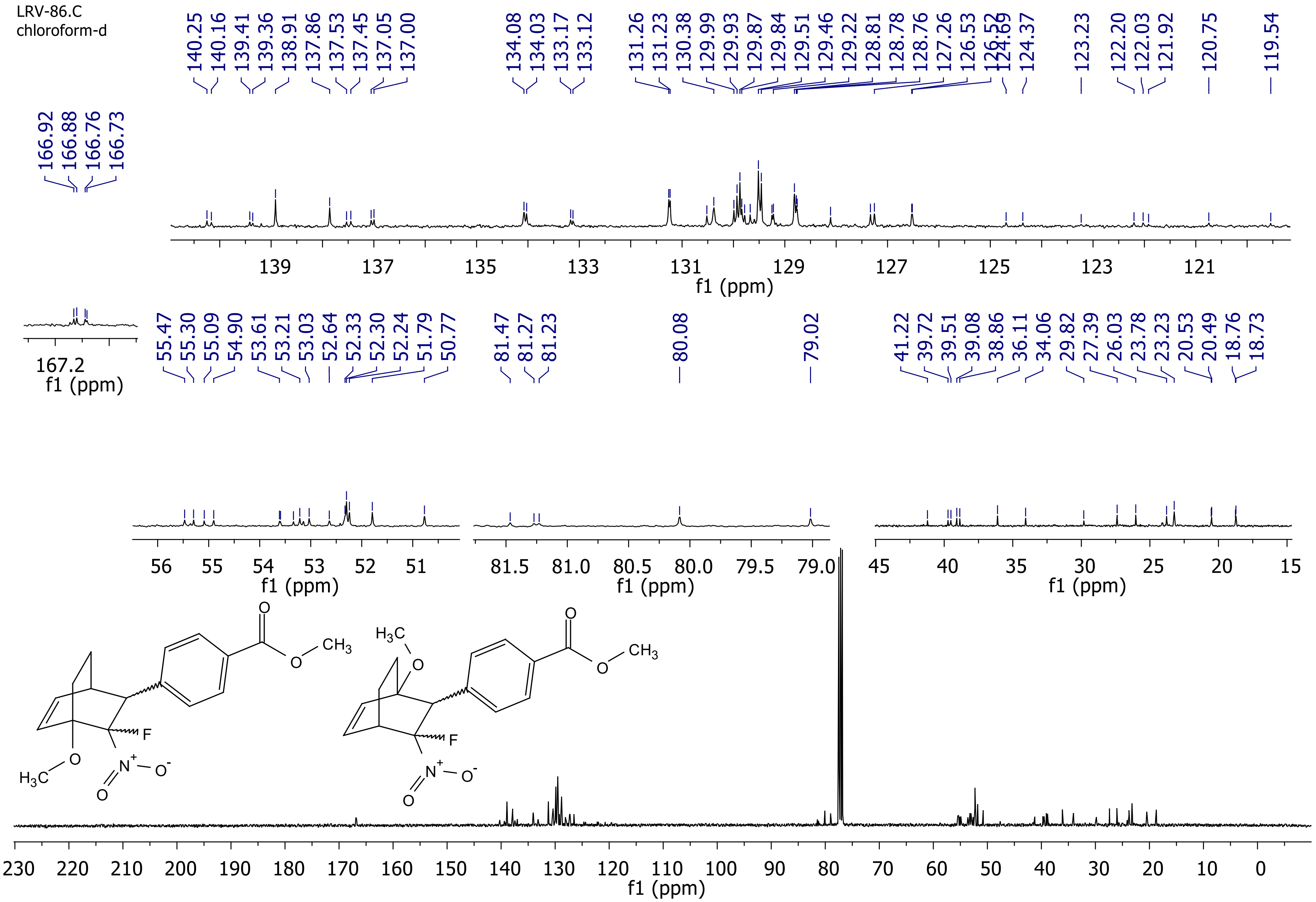

^{13}C NMR spectrum of methyl 4-(3-fluoro-4-methoxy-3-nitrobicyclo[2.2.2]oct-5-en-2-yl)benzoate and methyl 4-(3-fluoro-1-methoxy-3-nitrobicyclo[2.2.2]oct-5-en-2-yl)benzoate (**3d**)

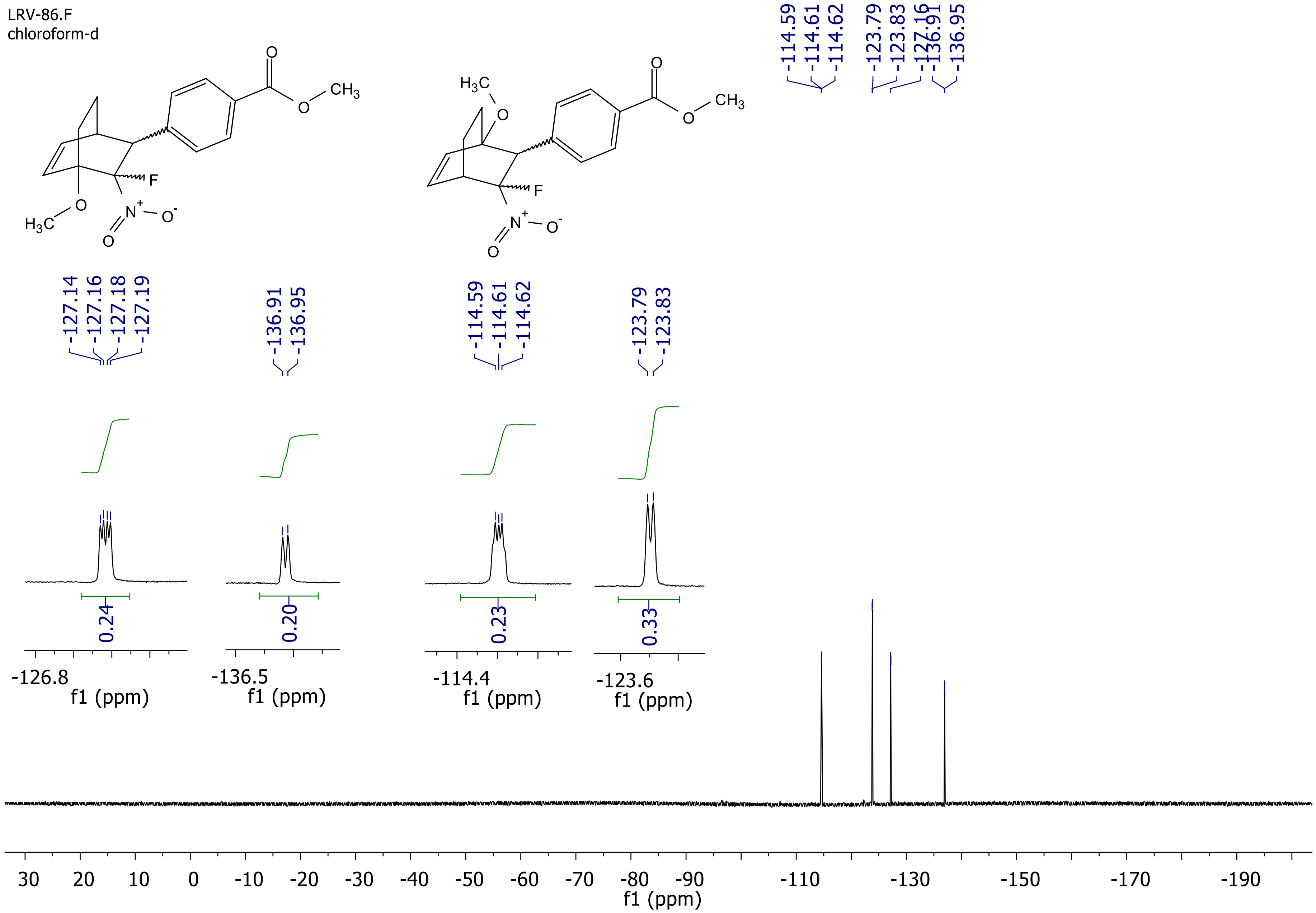

^{19}F NMR spectrum of methyl 4-(3-fluoro-4-methoxy-3-nitrobicyclo[2.2.2]oct-5-en-2-yl)benzoate and methyl 4-(3-fluoro-1-methoxy-3-nitrobicyclo[2.2.2]oct-5-en-2-yl)benzoate (**3d**)

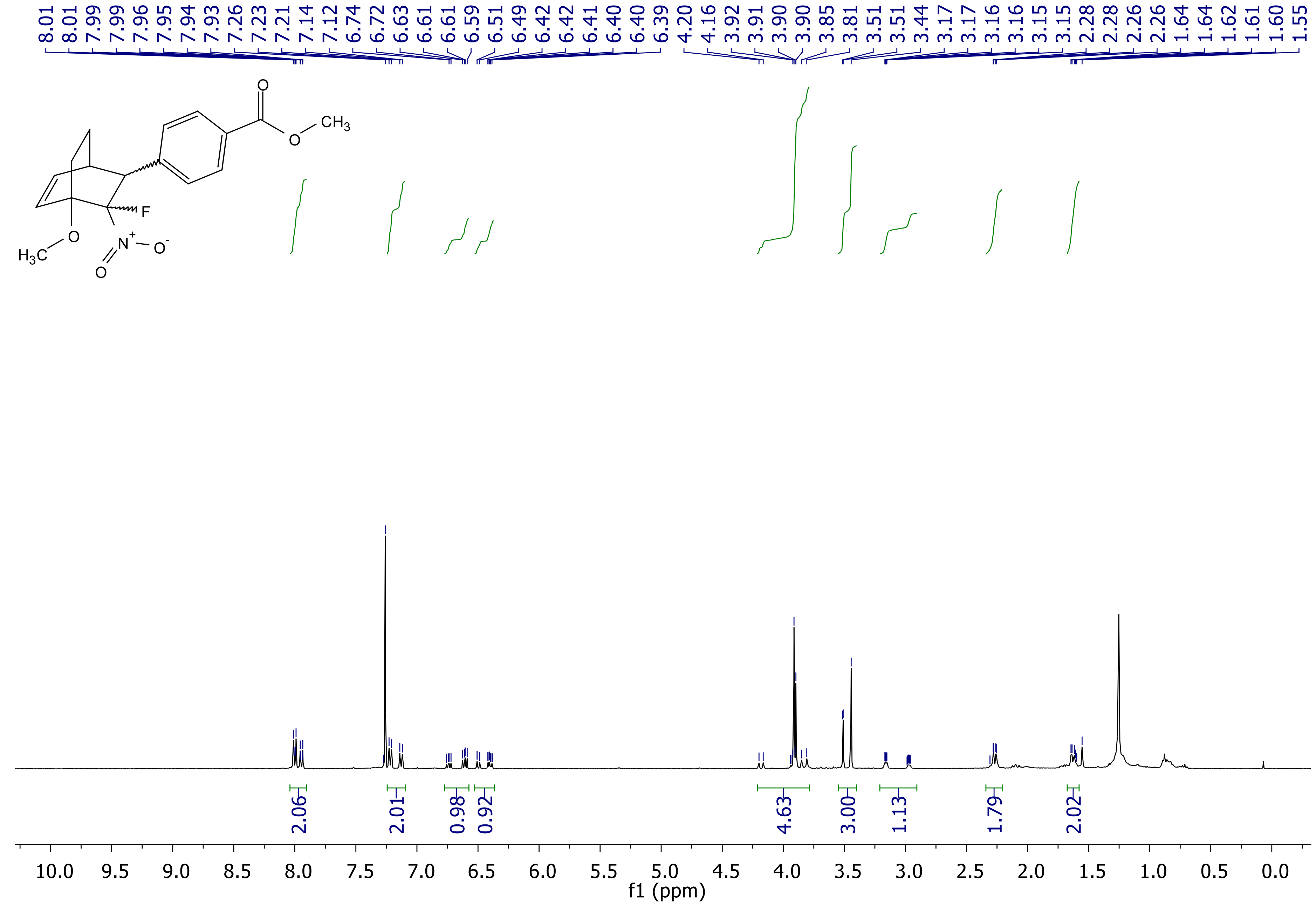

^{1}H NMR spectrum of methyl 4-(3-fluoro-4-methoxy-3-nitrobicyclo[2.2.2]oct-5-en-2-yl)benzoate (**3d**)

PSA-57-3.F
chloroform-d

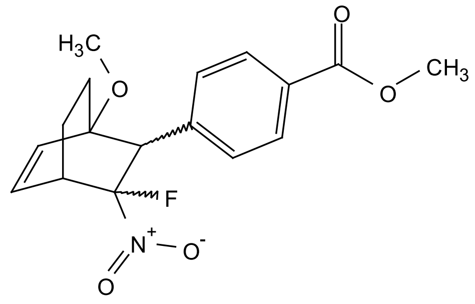

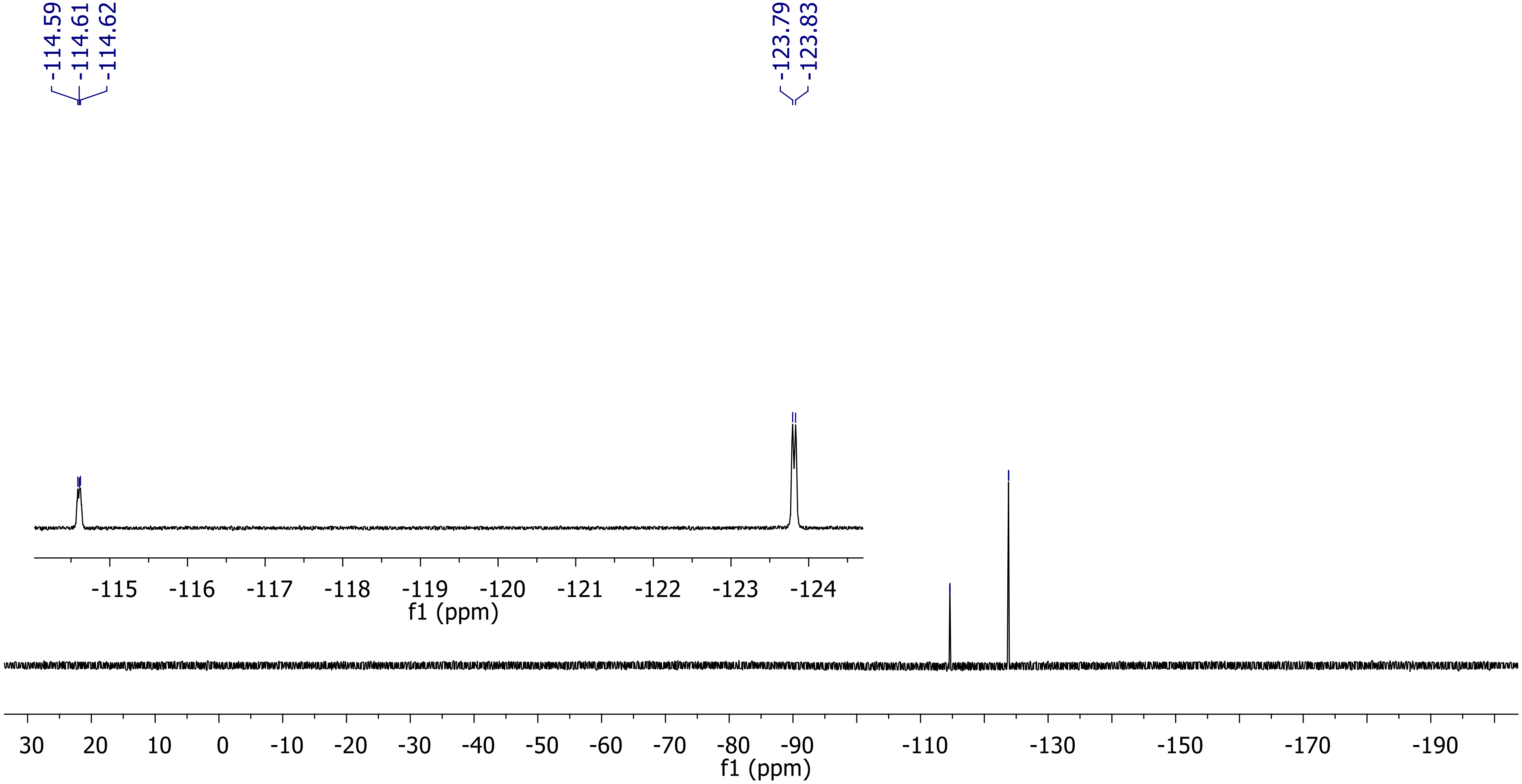

^{19}F NMR spectrum of 4-(3-fluoro-1-methoxy-3-nitrobicyclo[2.2.2]oct-5-en-2-yl)benzoate (**3d**)

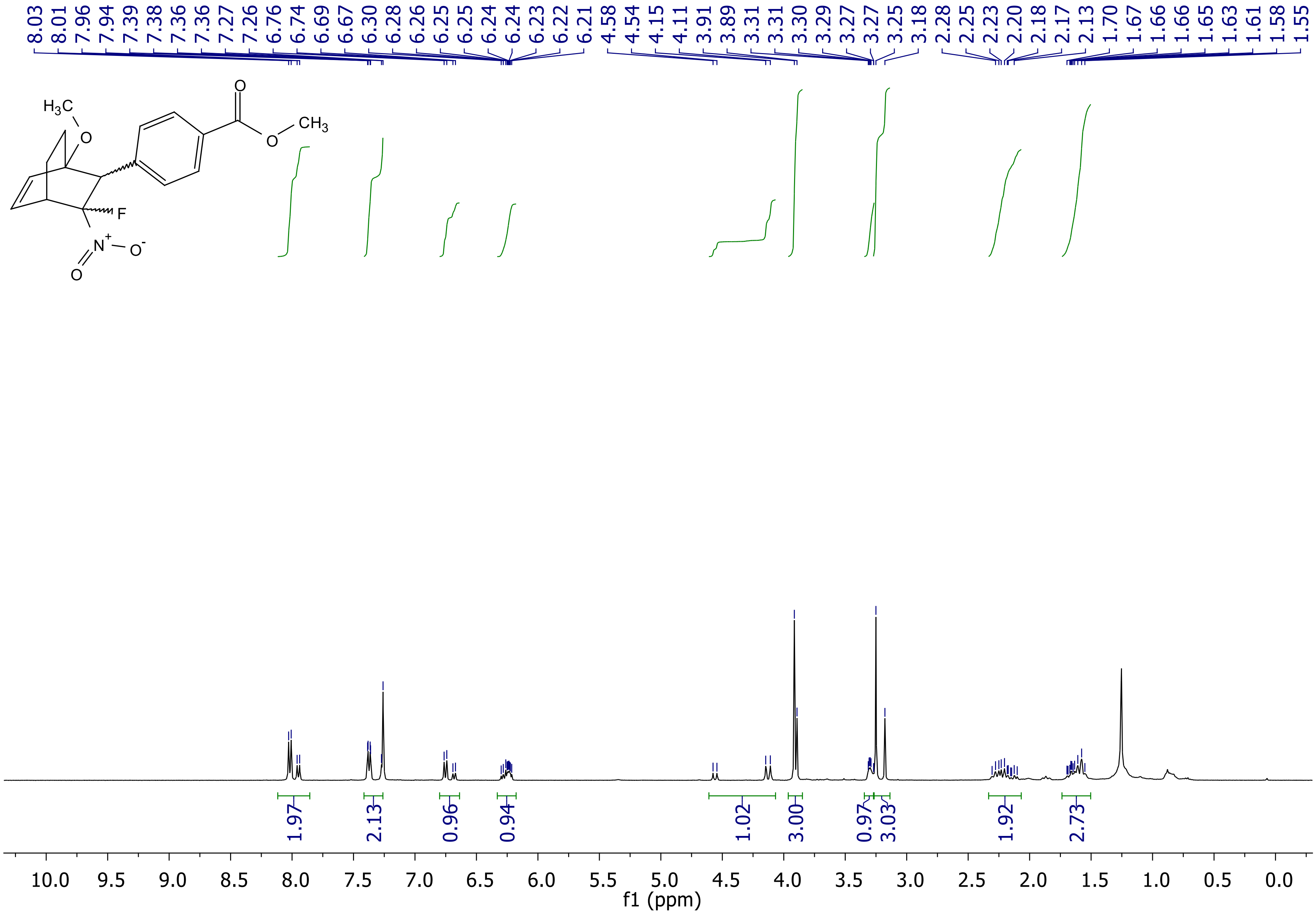

^{1}H NMR spectrum of 4-(3-fluoro-1-methoxy-3-nitrobicyclo[2.2.2]oct-5-en-2-yl)benzoate (**3d**)

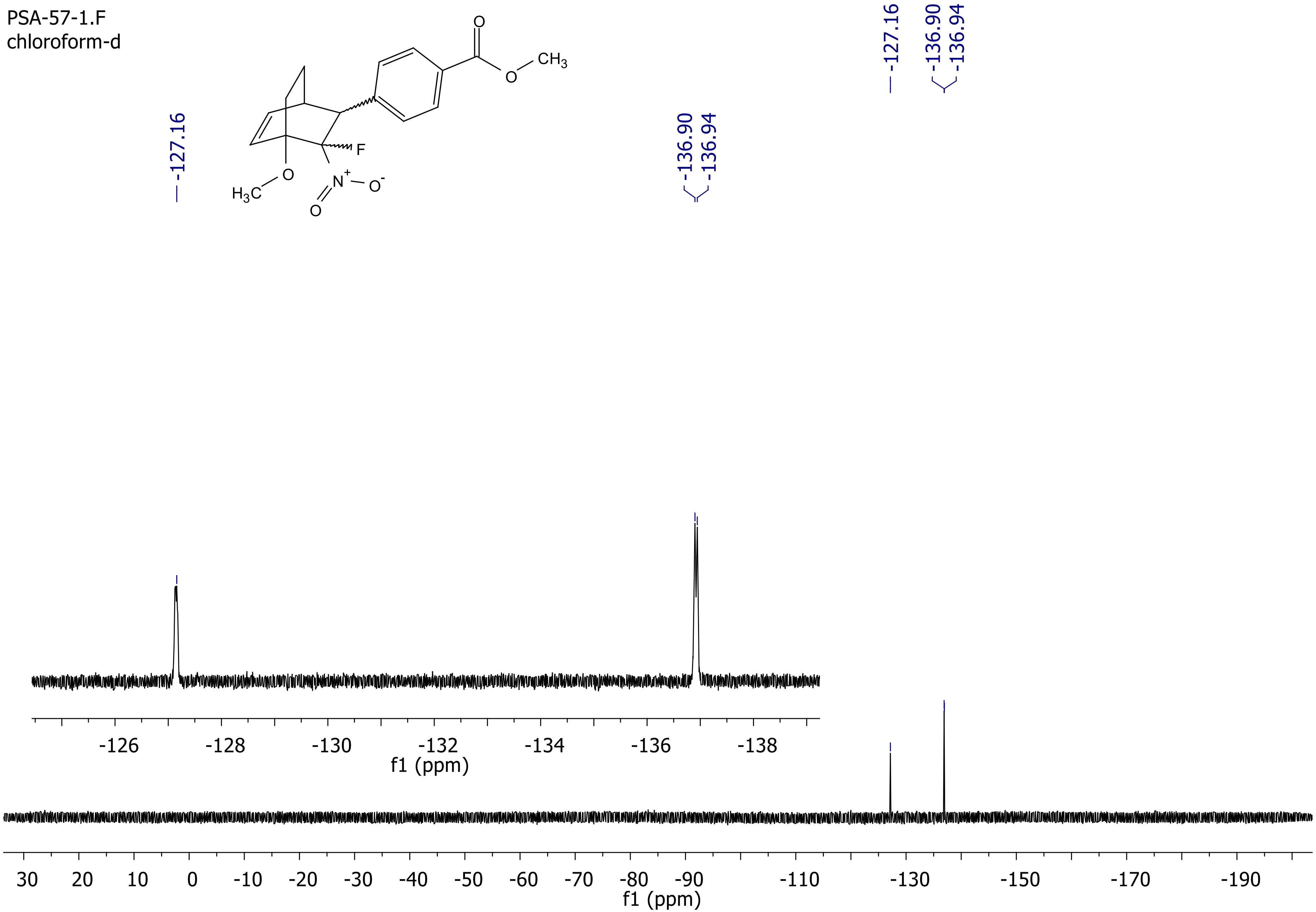

^{19}F NMR spectrum of methyl 4-(3-fluoro-4-methoxy-3-nitrobicyclo[2.2.2]oct-5-en-2-yl)benzoate (**3d**)

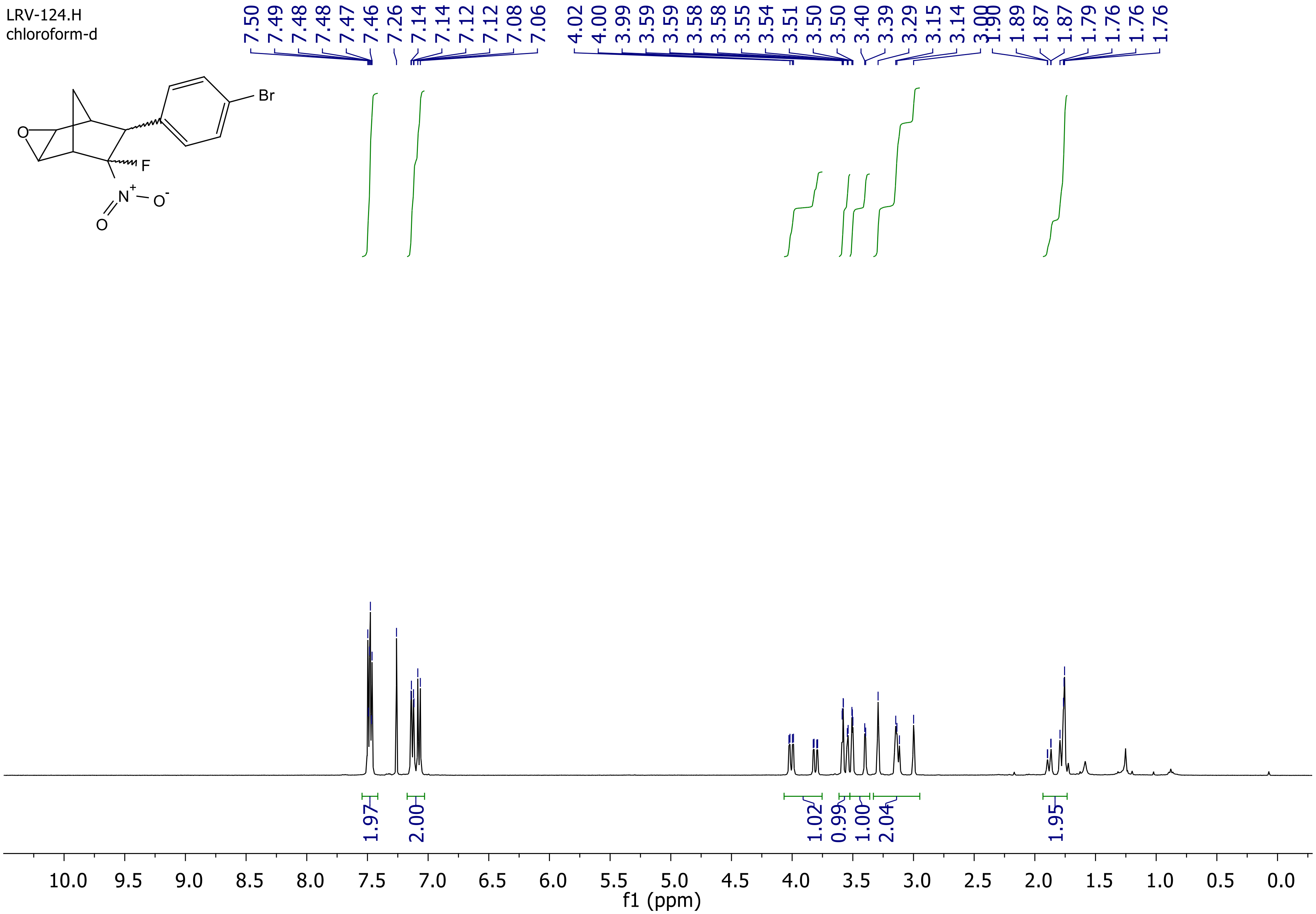

^{1}H NMR spectrum of 7-(4-bromophenyl)-6-fluoro-6-nitro-3-oxatricyclo[3.2.1.0[2,4]]octane (**4a**)

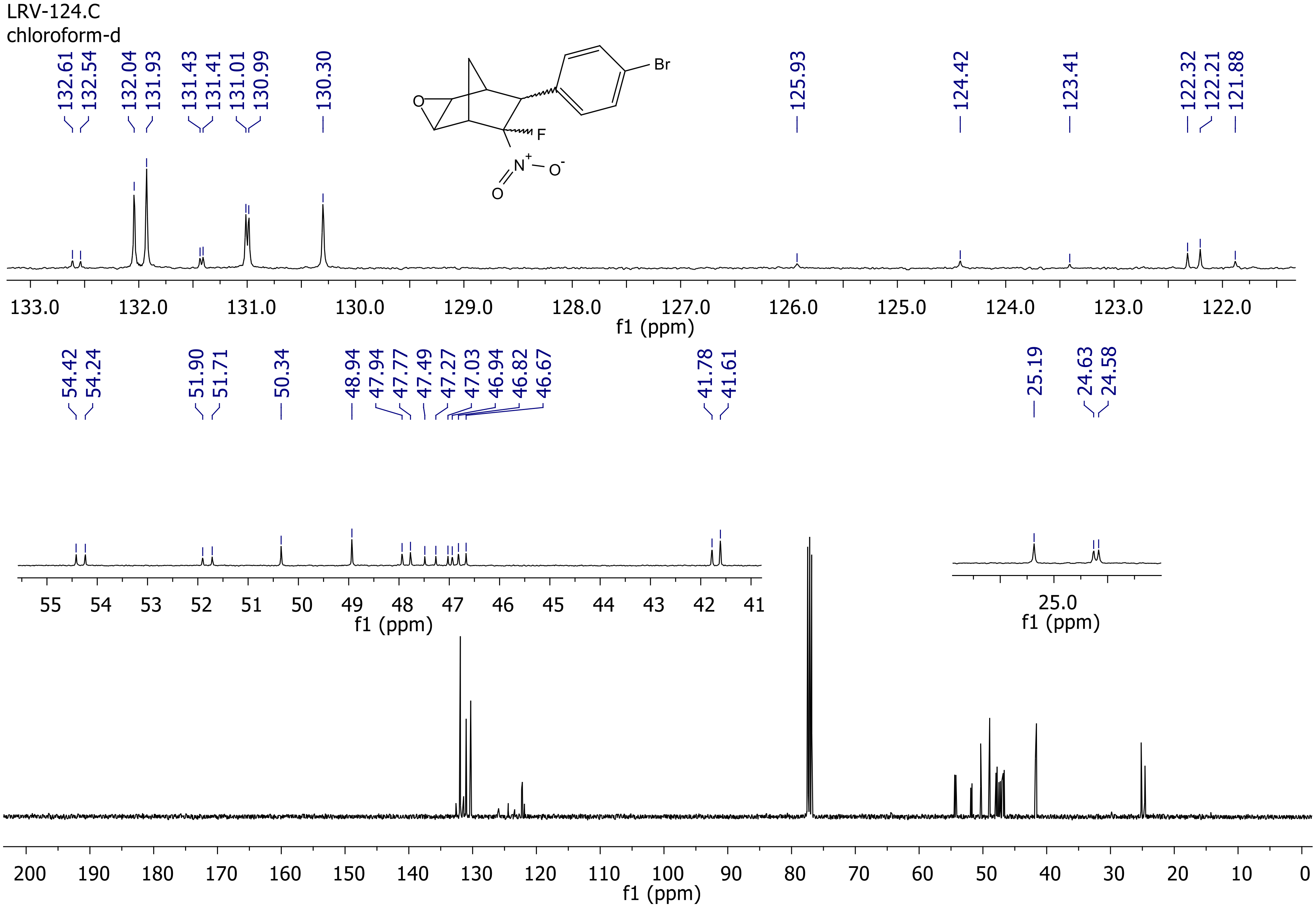

^{13}C NMR spectrum of 7-(4-bromophenyl)-6-fluoro-6-nitro-3-oxatricyclo[3.2.1.0^{2,4}]octane (**4a**)

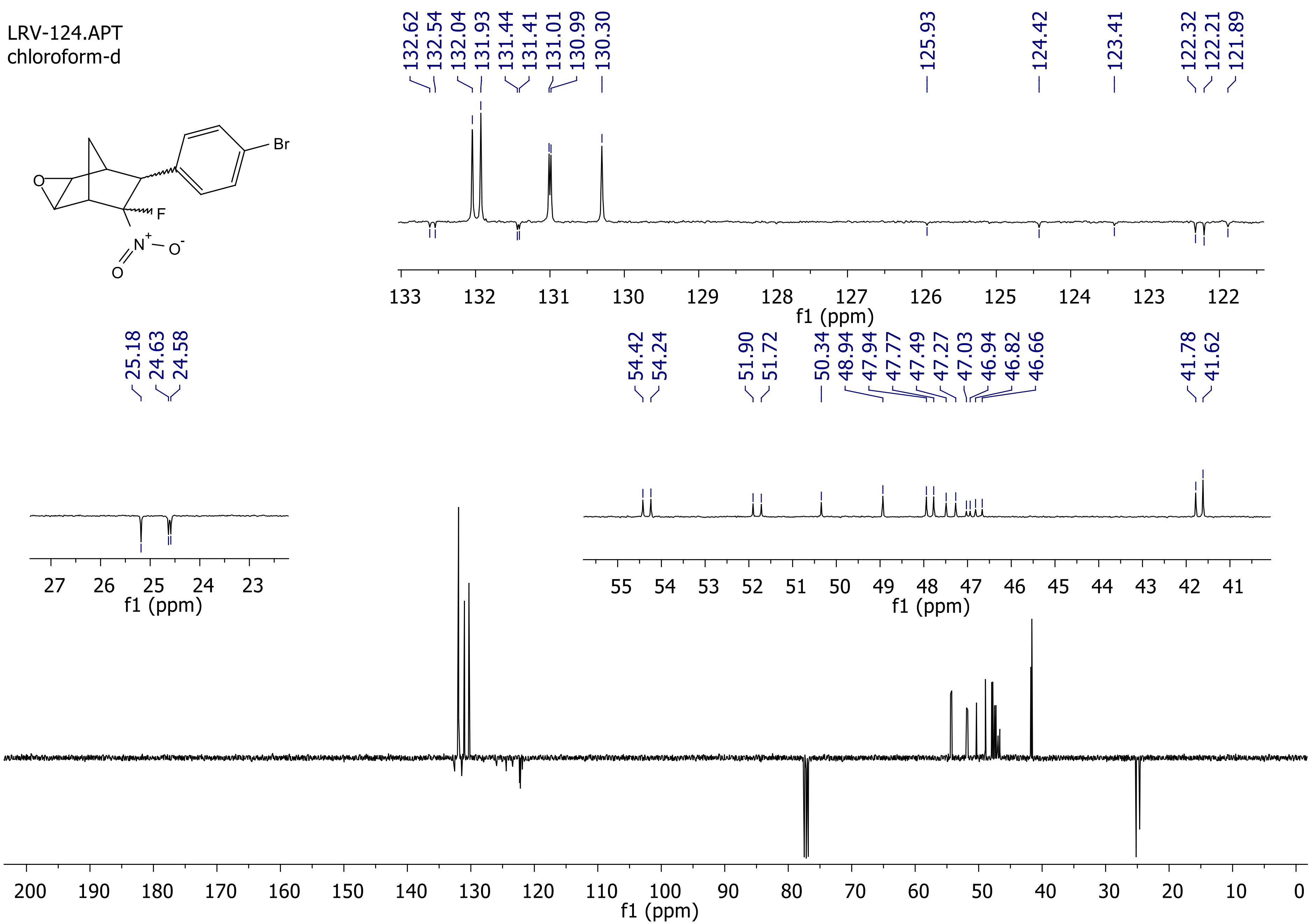

^{13}C APT NMR spectrum of 7-(4-bromophenyl)-6-fluoro-6-nitro-3-oxatricyclo[3.2.1.0^{2,4}]octane (**4a**)

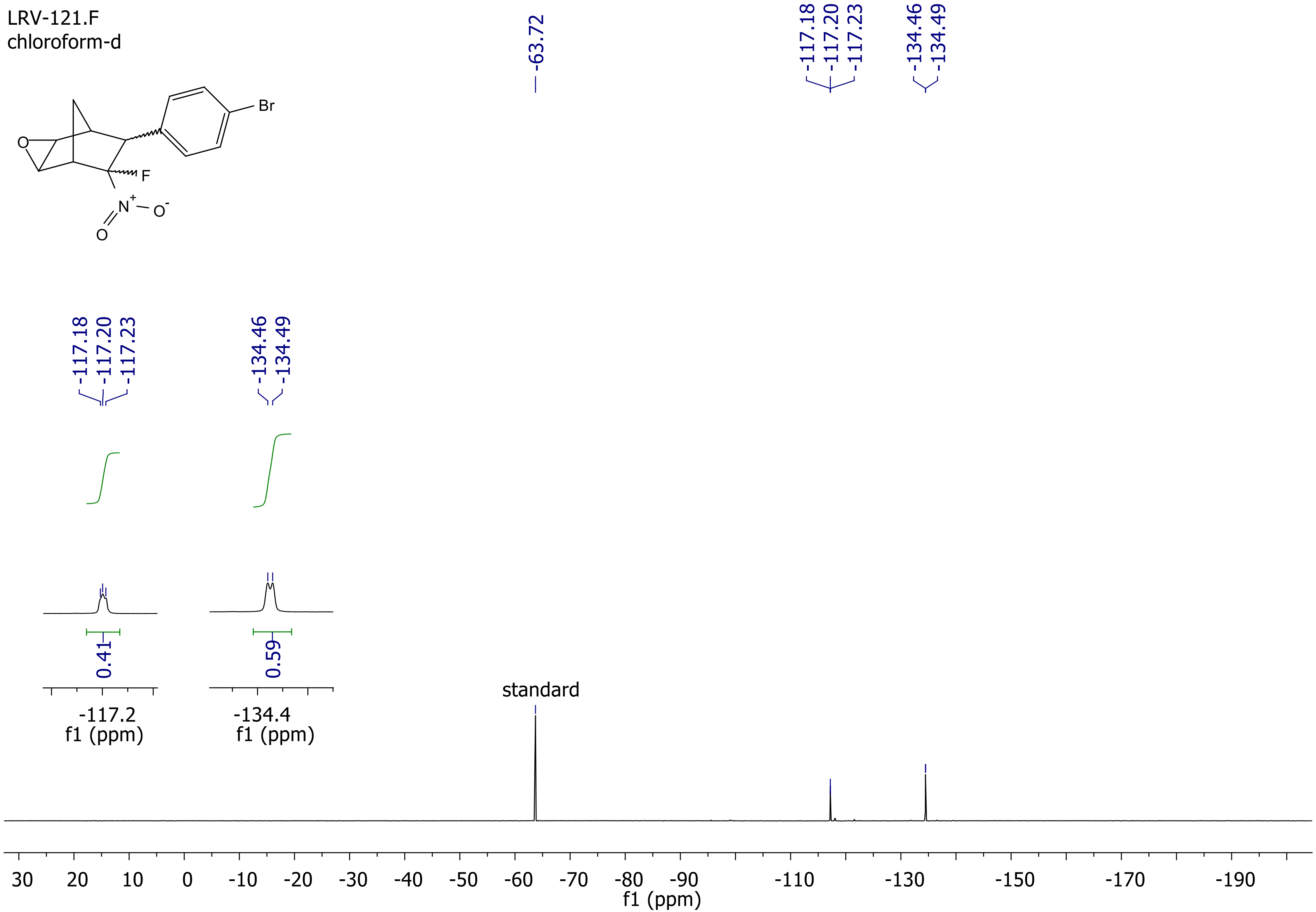

^{19}F NMR spectrum of 7-(4-bromophenyl)-6-fluoro-6-nitro-3-oxatricyclo[3.2.1.0^{2,4}]octane (**4a**)

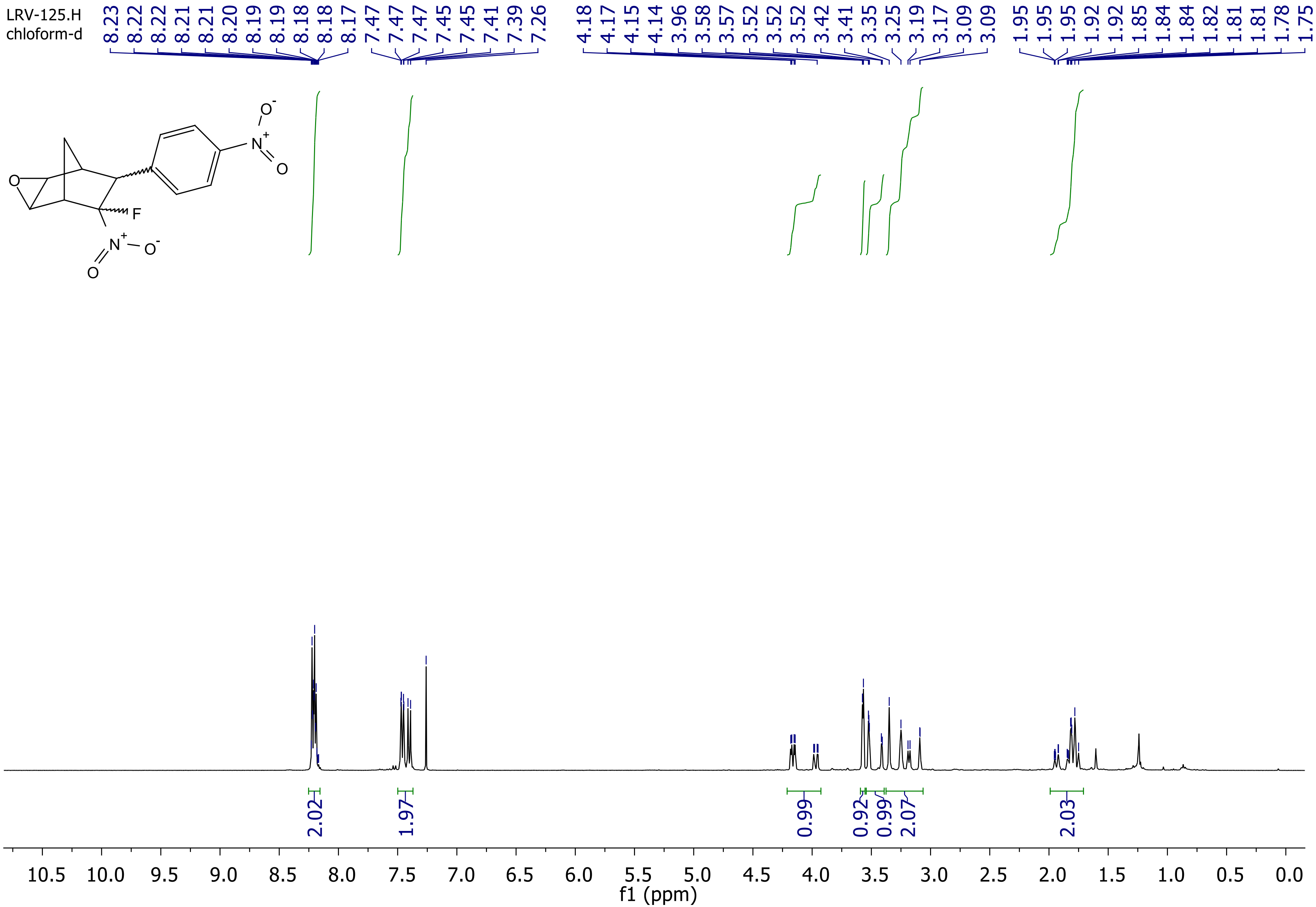

^{1}H NMR spectrum of 6-fluoro-6-nitro-7-(4-nitrophenyl)-3-oxatricyclo[3.2.1.0^{2,4}]octane (**4b**)

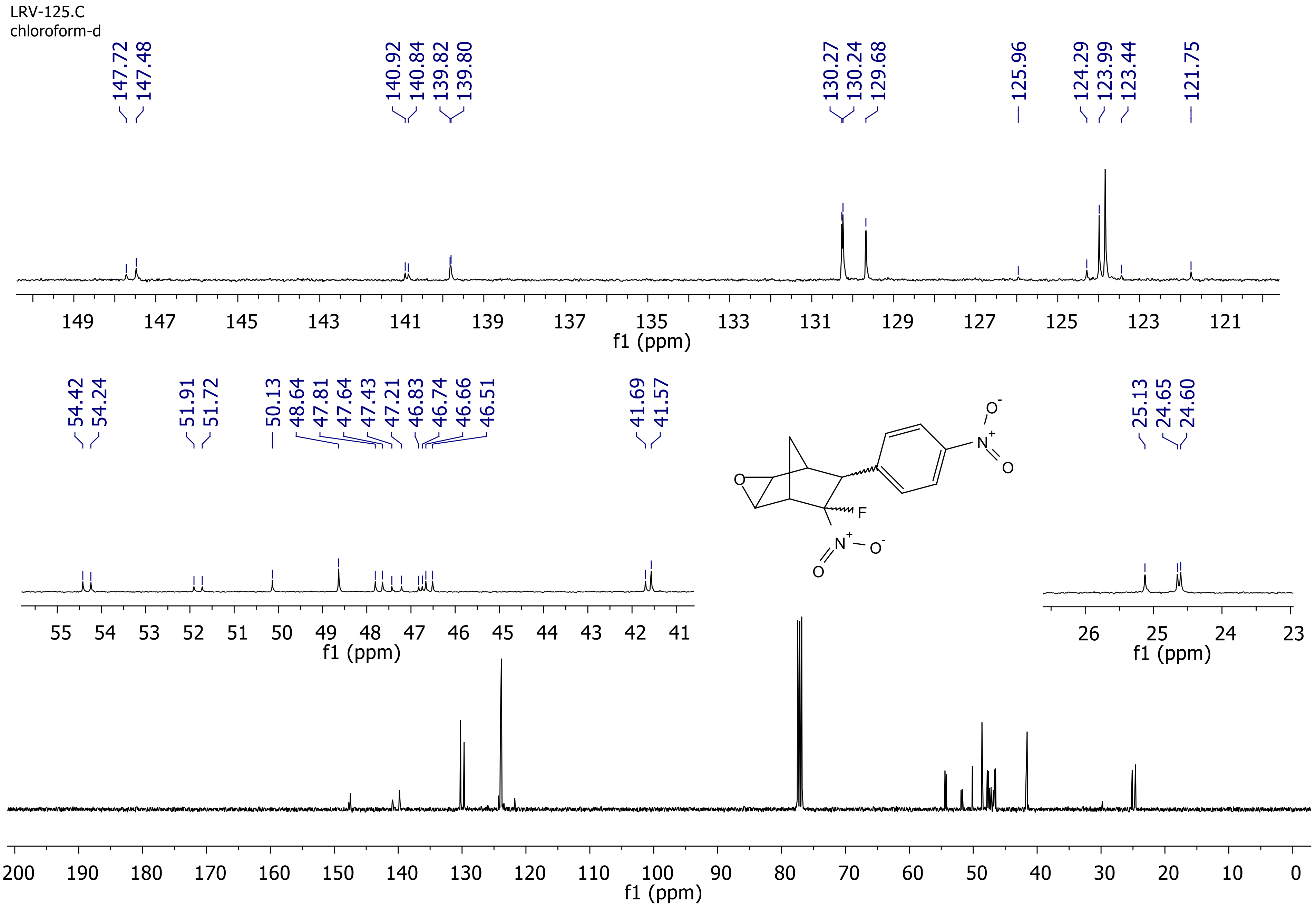

^{13}C NMR spectrum of 6-fluoro-6-nitro-7-(4-nitrophenyl)-3-oxatricyclo[3.2.1.0^{2,4}]octane (**4b**)

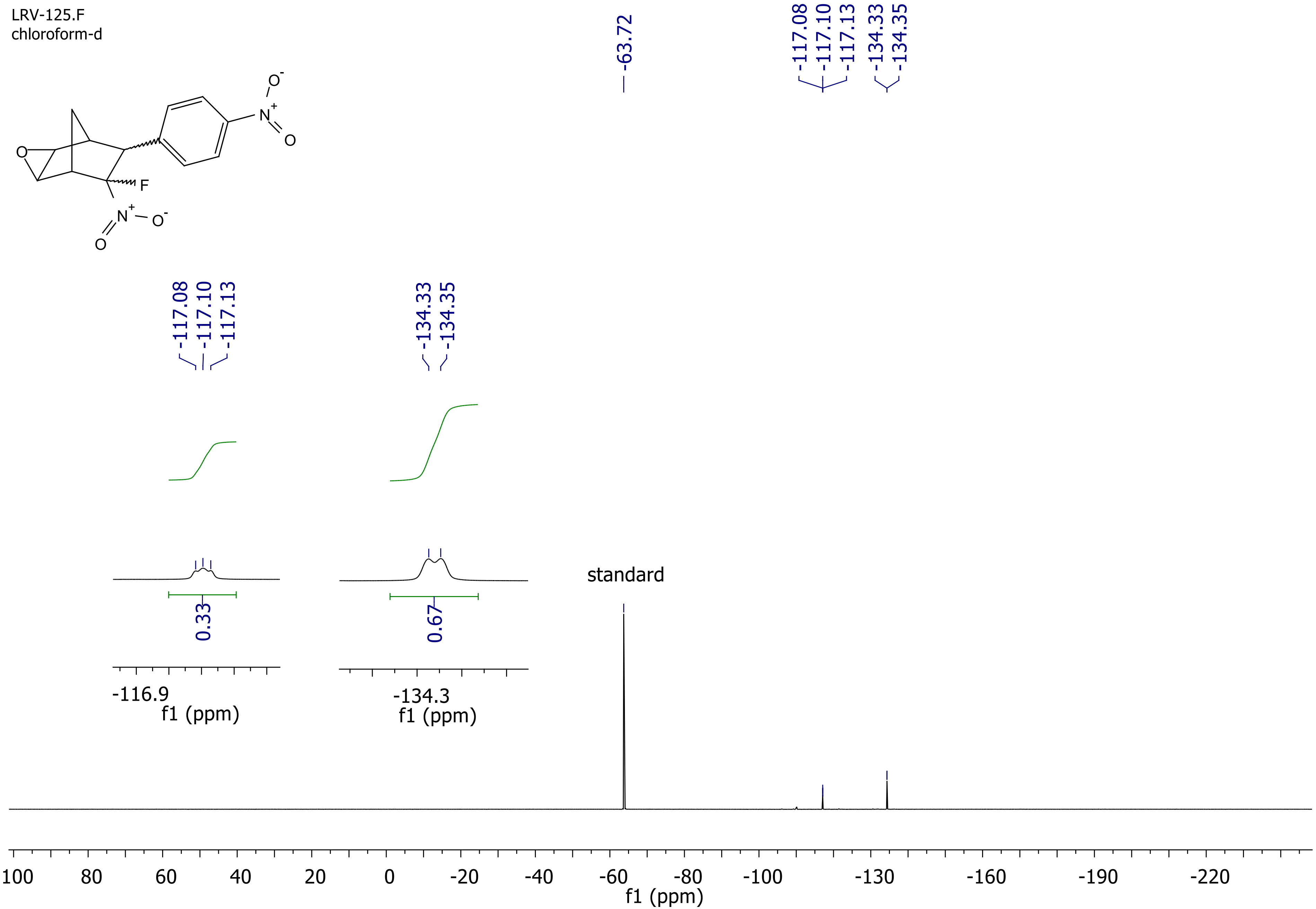

^{19}F NMR spectrum of 6-fluoro-6-nitro-7-(4-nitrophenyl)-3-oxatricyclo[3.2.1.0^{2,4}]octane (**4b**)

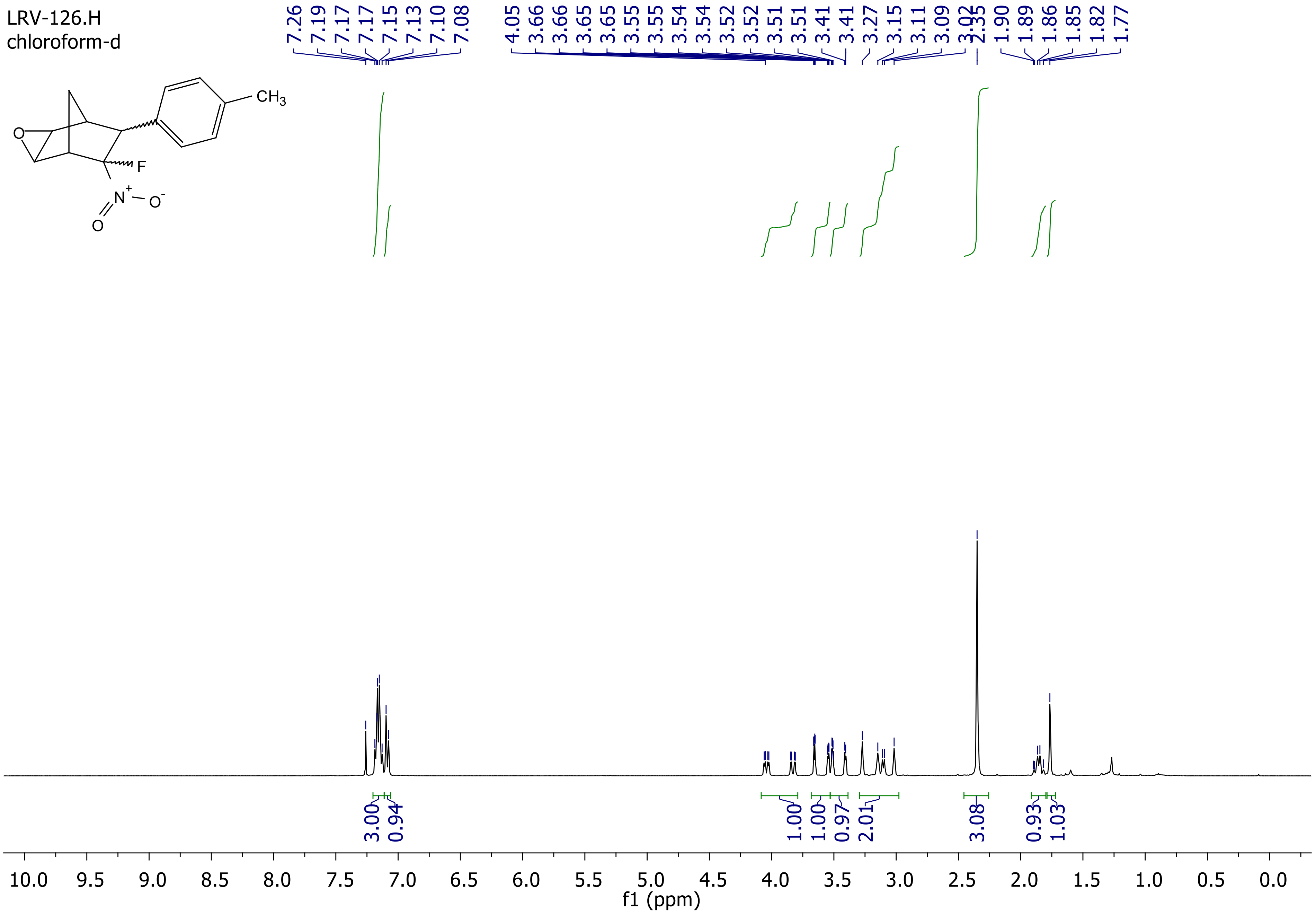

^{1}H NMR spectrum of 6-fluoro-6-nitro-7-(p-tolyl)-3-oxatricyclo[3.2.1.0^{2,4}]octane (**4c**)

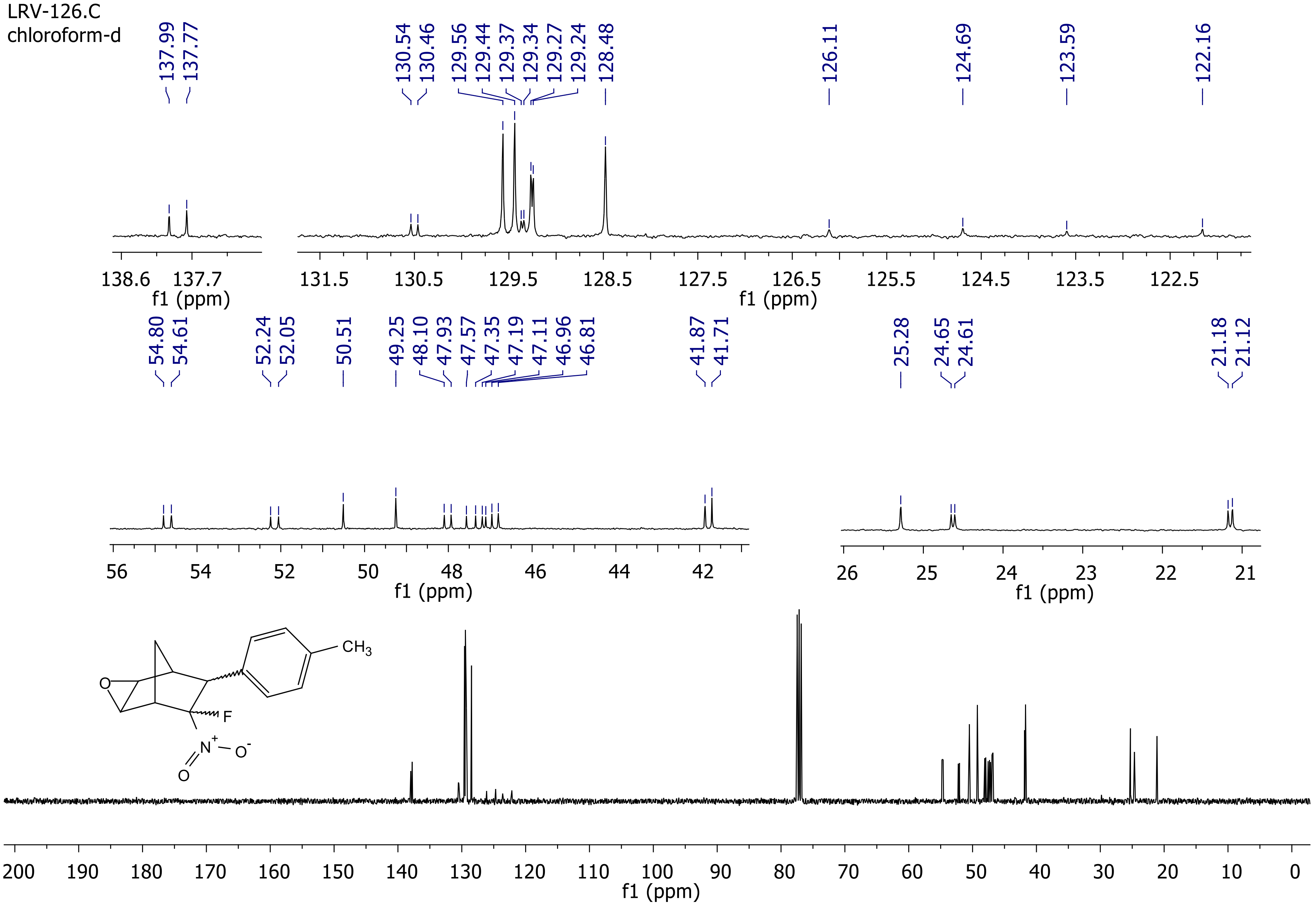

^{13}C NMR spectrum of 6-fluoro-6-nitro-7-(p-tolyl)-3-oxatricyclo[3.2.1.0^{2,4}]octane (**4c**)

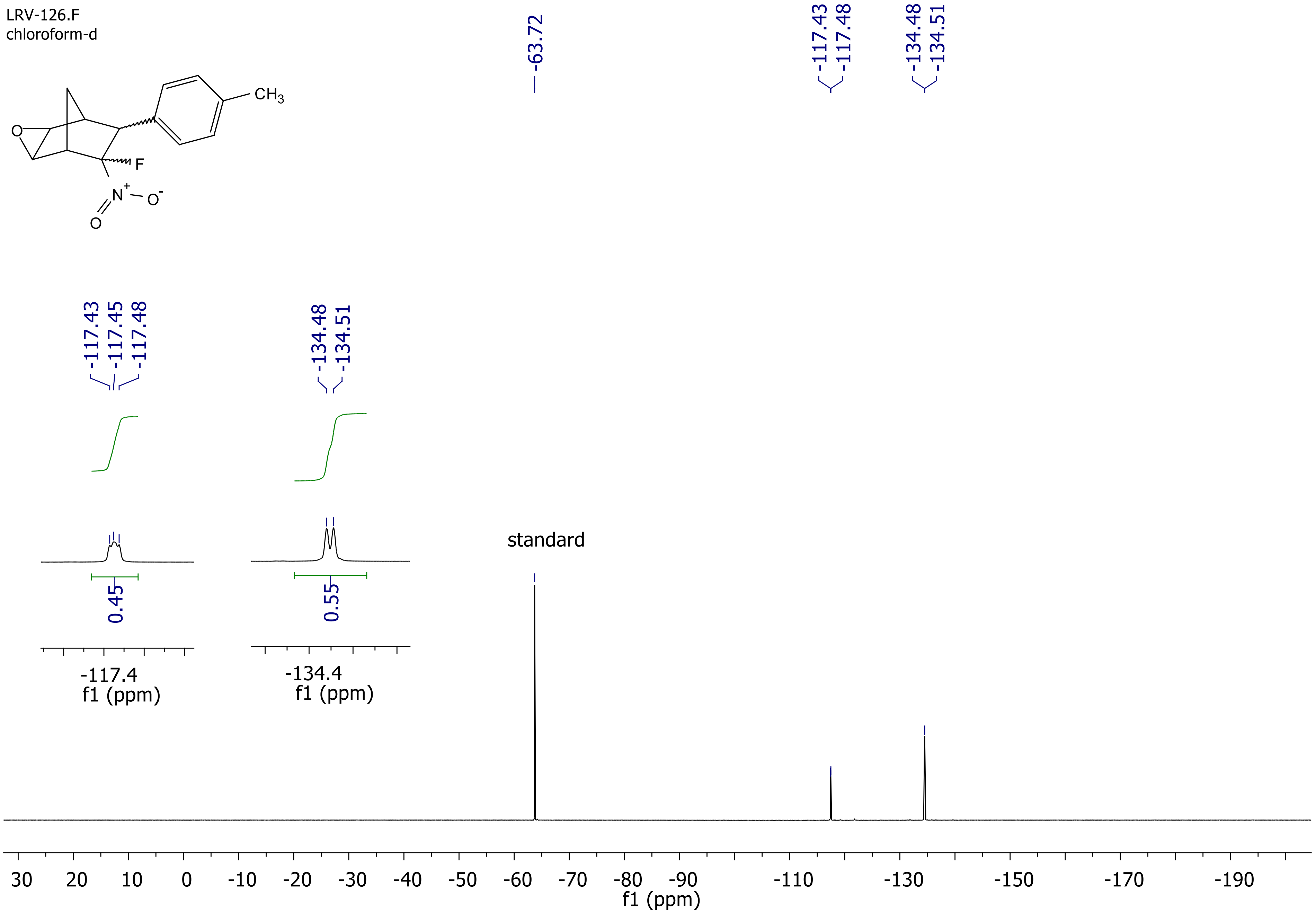

^{19}F NMR spectrum of 6-fluoro-6-nitro-7-(p-tolyl)-3-oxatricyclo[3.2.1.0^{2,4}]octane (**4c**)

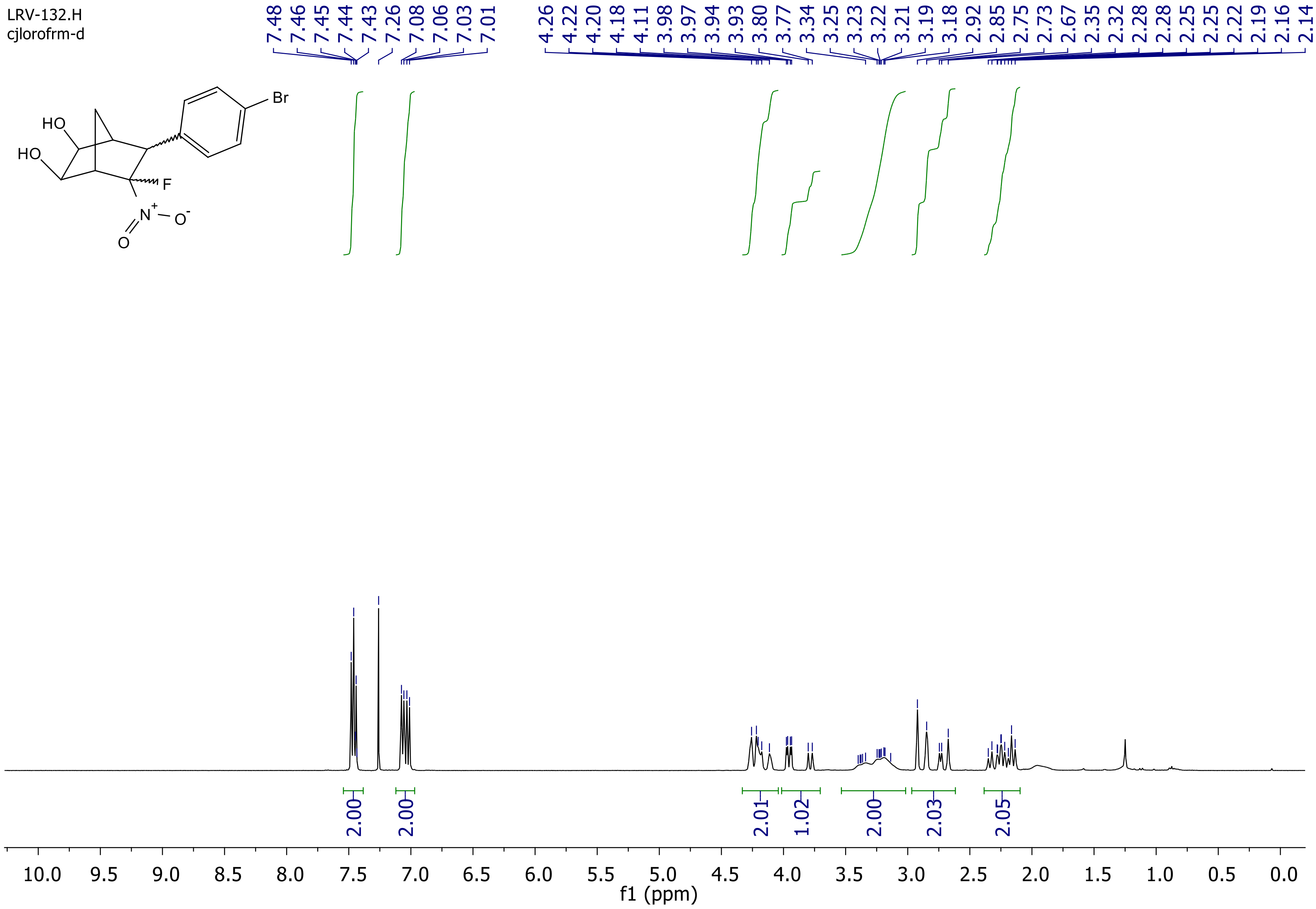

^{1}H NMR spectrum of 6-(4-bromophenyl)-5-fluoro-5-nitrobicyclo[2.2.1]heptane-2,3-diol (**5**)

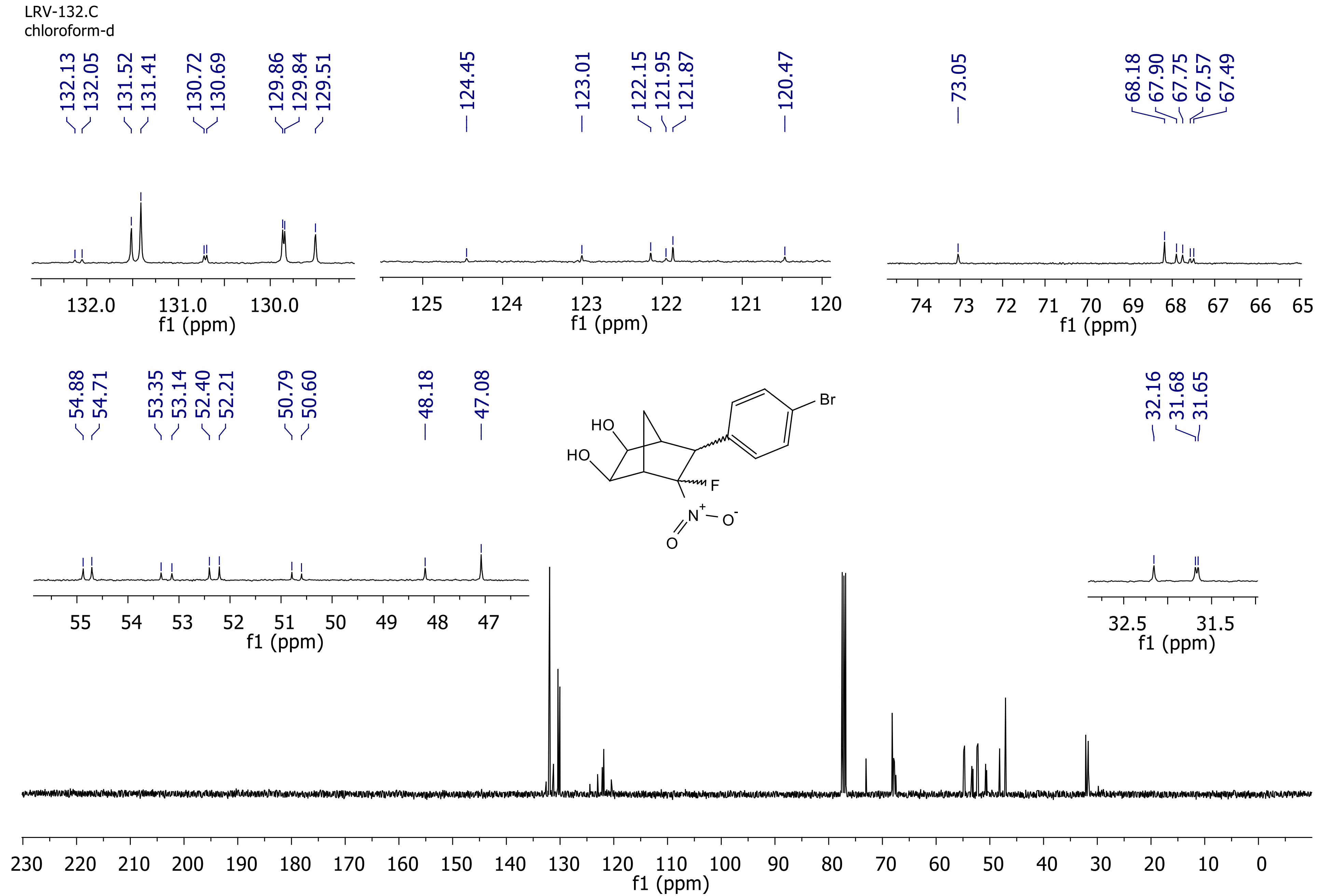

^{13}C NMR spectrum of 6-(4-bromophenyl)-5-fluoro-5-nitrobicyclo[2.2.1]heptane-2,3-diol (**5**)

LRV-132.F
chloroform-d

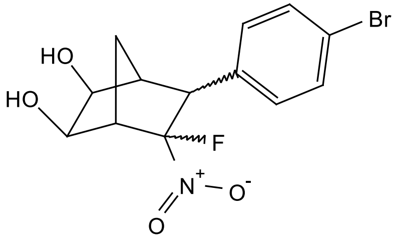

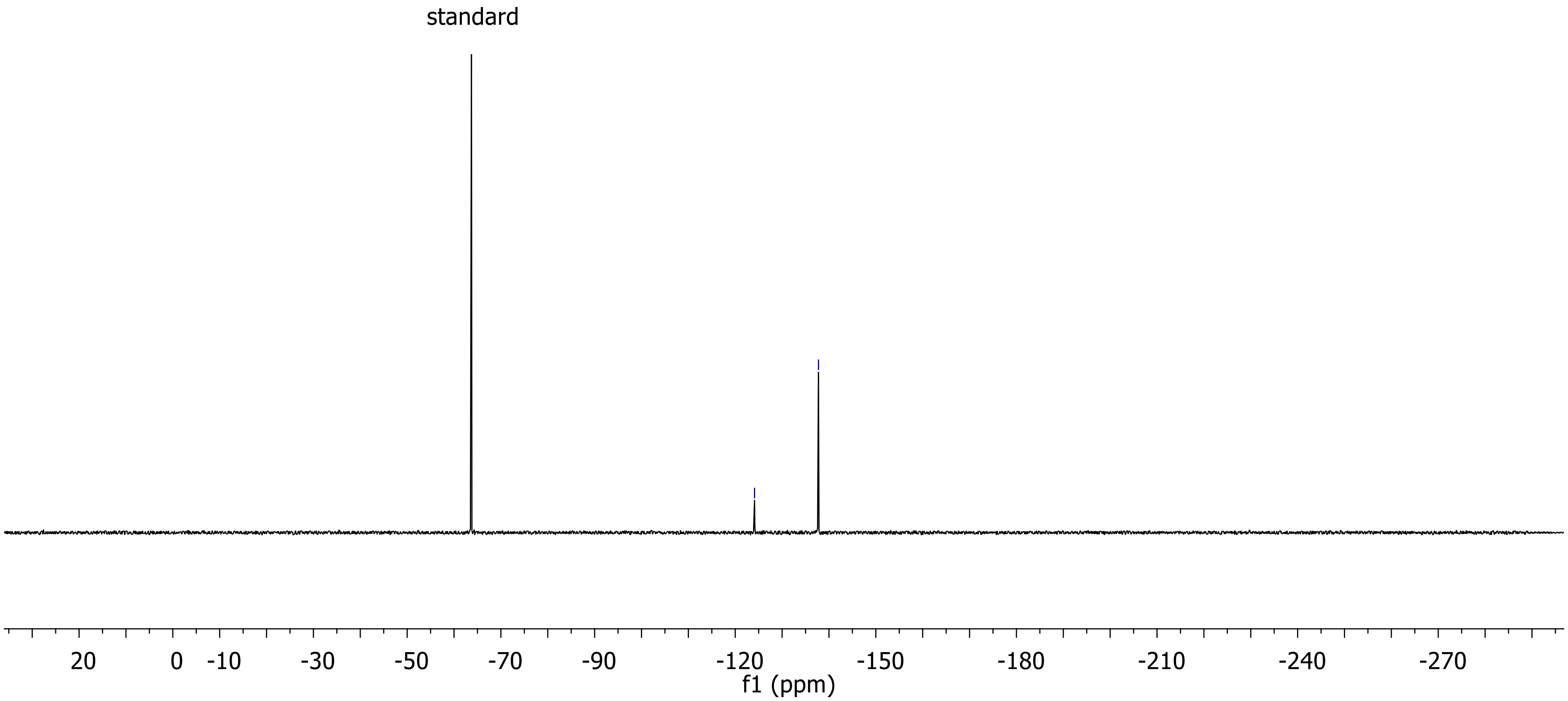

^{19}F NMR spectrum of 6-(4-bromophenyl)-5-fluoro-5-nitrobicyclo[2.2.1]heptane-2,3-diol (**5**)

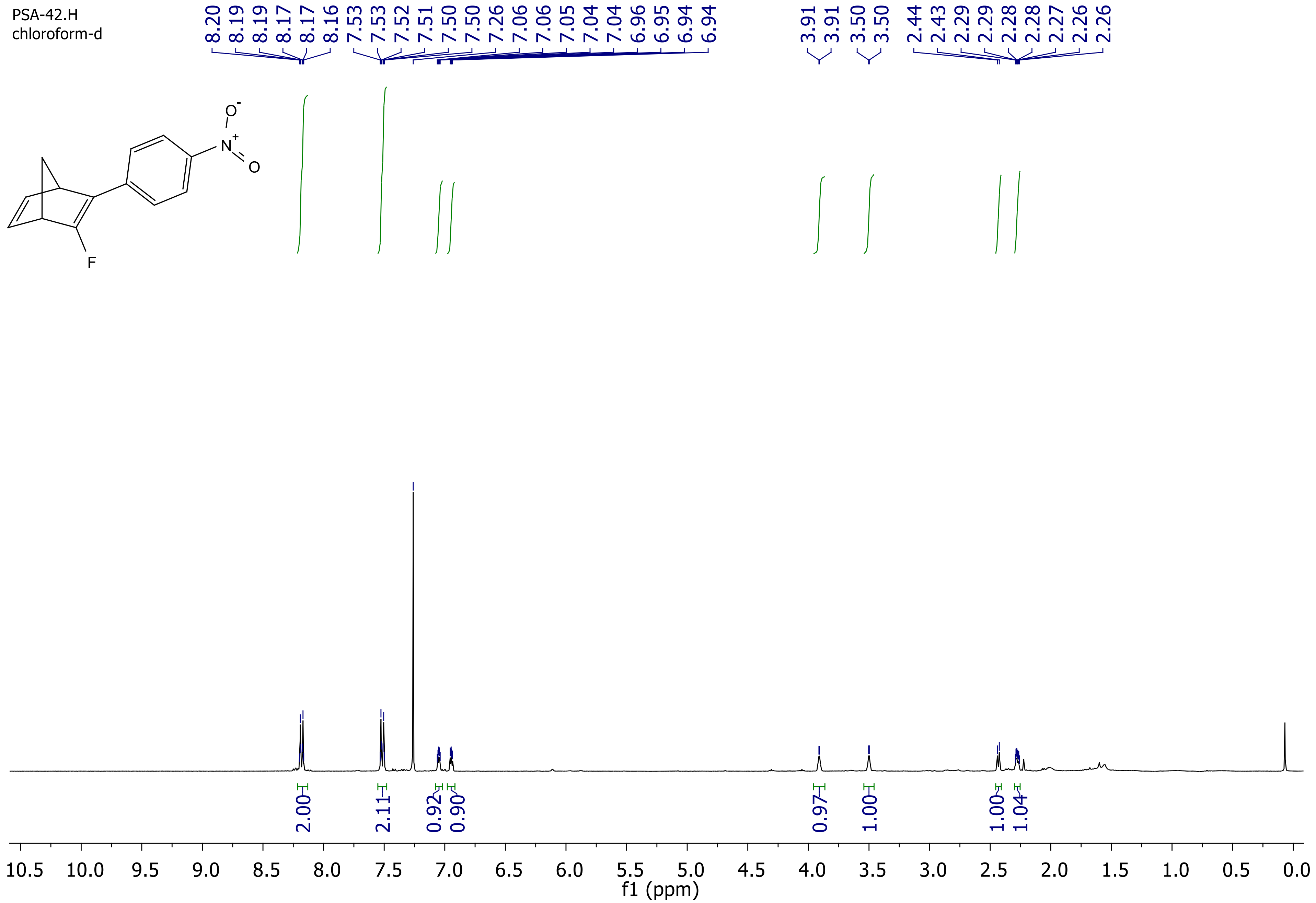

^{1}H NMR spectrum of 2-fluoro-3-(4-nitrophenyl)bicyclo[2.2.1]hepta-2,5-diene (**6**)

PSA-42.F
chloroform-d

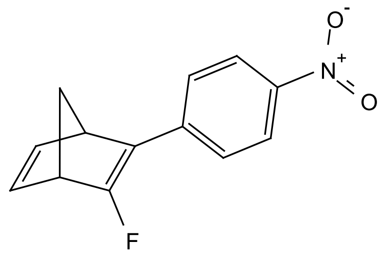

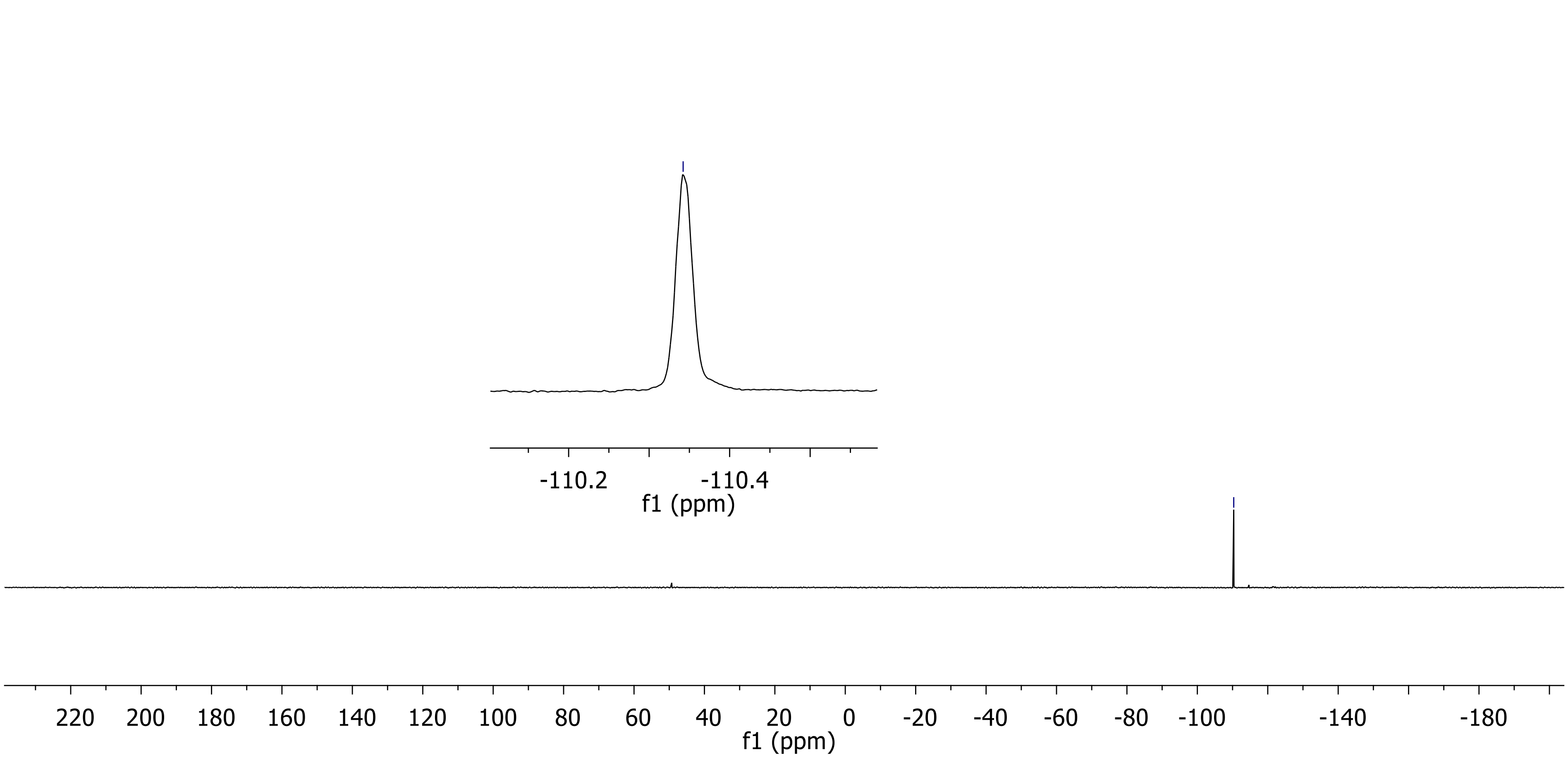

^{19}F NMR spectrum of 2-fluoro-3-(4-nitrophenyl)bicyclo[2.2.1]hepta-2,5-diene (**6**)

2. Experimental section

All reagents were purchased from commercial sources and used without any further purification. CPD was prepared by monomerization of dicyclopentadiene (DCPD). *o*-Xylene was dried before use by passing through a column charged with activated neutral alumina [1]. Melting points (M.p.) were measured with a Büchi B-545 melting point apparatus. NMR (^{1}H, ^{13}C and ^{19}F) spectra were obtained with Bruker AV-400 and Agilent 400-MR spectrometers using deuterated chloroform ($CDCl_3$). Chemical shifts for ^{1}H NMR spectroscopic data were referenced to internal tetramethylsilane (δ = 0.0 ppm) and the residual solvent resonance (δ = 7.26 ppm); chemical shifts for ^{13}C NMR spectroscopic data were referenced to the residual solvent resonance (δ = 77.16 ppm); chemical shifts for ^{19}F NMR spectroscopic data were referenced to $PhCF_3$ (δ = −63.72 ppm). Data are reported as follows: chemical shift, integration multiplicity (s = singlet, d = doublet, t = triplet, q = quadruplet, qui = quintet, sext = sextet, sept = septet, br = board, m = multiplet, dd = *doublet of doublets*, ddd = *doublet of doublet of doublets*) and coupling constants (Hz). The starting β-fluoro-β-nitrostyrenes were prepared according to the described procedure and all are known compounds [2,3].

General procedure for the Diels–Alder reaction of β-fluoro-β-nitrostyrenes and 1,3-dienes

Analogous as described in [3], in a typical experiment, β-fluoro-β-nitrostyrene **1** (0.5 mmol, 1 mol equiv), *o*-xylene (0.2 mL), and diene (2.5–5.0 mmol, 5–10 mol equiv) were successively loaded into a screw-top vial filled with argon. After the cap was screwed tightly, the reaction mixture was heated at 110–130 °C with vigorous stirring for the appropriate time (8–24 h). After completion of the reaction (^{1}H NMR analysis monitoring), the excess of the diene and *o*-xylene were evaporated under vacuum. The pure product was isolated by column chromatography using mixture of Hex/DCM as eluent.

(1*R,4*S**,5*R**,6*R**)-5-Fluoro-5-nitro-6-phenylbicyclo[2.2.1]hept-2-ene** (*endo*-**2a**; major isomer) and **(1*R**,4*S**,5*S**,6*S**)-5-fluoro-5-nitro-6-phenylbicyclo[2.2.1]hept-2-ene** (*exo*-**2a**; minor isomer). Eluent: Hex/DCM 4:1, Hex/DCM 1:1; 0.097 g (74 %); *dr* = 45:55; yellowish oil. Anal. calcd for $C_{13}H_{12}FNO_2$ (%): C, 66.94; H, 5.19; N, 6.01; Found: C, 66.88; H, 5.31; N, 6.21. ^{1}H NMR (400 MHz, $CDCl_3$): (major isomer) δ = 1.88 – 2.00 (m, 1H), 2.38 (dd, *J* = 9.6, 0.7 Hz, 1H), 3.37 (s, 1H), 3.52 (s, 1H), 4.21 (dd, *J* = 9.4, 3.0 Hz, 1H), 6.30 (dd, *J* = 5.3, 3.5 Hz, 1H), 6.68 – 6.80 (m, 1H), 7.18 (d, *J* = 7.5 Hz, 1H), 7.26 – 7.45 (m, 4H) ppm; (minor isomer) δ = 2.12 (dd, *J* = 9.6, 1.3 Hz, 1H), 2.45 (d, *J* = 9.5 Hz, 1H), 3.34 (s, 1H), 3.38 – 3.42 (m, 1H), 3.84 (dd, *J* = 10.9, 2.8 Hz, 1H), 6.17 – 6.22 (m, 1H), 6.68 – 6.80 (m, 1H), 7.18 (d, *J* = 7.5 Hz, 1H), 7.26 – 7.45 (m, 4H) ppm; ^{13}C NMR (100 MHz, $CDCl_3$): (major isomer) δ = 47.6, 48.9 (d, $^{3}J_{CF}$ = 1.1 Hz), 53.6 (d, $^{2}J_{CF}$ = 20.5 Hz), 55.9 (d, $^{2}J_{CF}$ = 17.8 Hz), 125.6 (d, $^{1}J_{CF}$ = 252.3 Hz), 127.7, 128.4, 128.7, 132.5 (d, $^{3}J_{CF}$ = 4.4 Hz), 135.1 (d, $^{3}J_{CF}$ = 3.4 Hz), 140.3 (d, $^{4}J_{CF}$ = 1.2 Hz) ppm; (minor isomer) δ = 46.4, 48.0, 51.6 (d, $^{2}J_{CF}$ = 22.5 Hz), 52.8 (d, $^{2}J_{CF}$ = 19.2 Hz), 125.8 (d, $^{1}J_{CF}$ = 253.2 Hz), 127.7, 128.6, 129.2 (d, $^{4}J_{CF}$ = 1.1 Hz), 132.7 (d, $^{3}J_{CF}$ = 6.3 Hz), 135.2 (d, $^{3}J_{CF}$ = 7.1 Hz), 143.4 (d, $^{4}J_{CF}$ = 2.2 Hz) ppm; ^{19}F NMR (376 MHz, $CDCl_3$): (major isomer) δ = -127.56 (s) ppm; (minor isomer) δ = -122.81 (s) ppm.

(1*R,4*S**,5*R**,6*R**)-5-Fluoro-5-nitro-6-(*p*-tolyl)bicyclo[2.2.1]hept-2-ene** (*endo*-**2b**; major isomer) and **(1*R**,4*S**,5*S**,6*S**)-5-fluoro-5-nitro-6-(*p*-tolyl)bicyclo[2.2.1]hept-2-ene** (*exo*-**2b**; minor isomer). Eluent: Hex/DCM 3:1; Hex/DCM, 2:1; Hex/DCM, 1:1. 0.238 g (70 %); *dr* = 48:52; yellowish oil. Anal. calcd for $C_{14}H_{14}FNO_2$ (%):C, 68.00; H, 5.71; N, 5.66; Found: C, 68.19; H, 5.67; N, 5.58. ^1H NMR (400 MHz, $CDCl_3$): (major isomer) δ = 1.93 – 2.00 (m, 1H), 2.41 (s, 3H), 2.55 – 2.51 (m, 1H), 3.37 (s, 1H), 3.54 (s, 1H), 4.20 (dd, *J* = 9.4, 3.0 Hz, 1H), 6.31 (dd, *J* = 5.4, 3.4 Hz, 1H), 6.78 (dd, *J* = 5.6, 2.9 Hz, 1H), 7.17 (d, *J* = 8.1 Hz, 2H), 7.23 (s, 2H) ppm; (minor isomer) δ = 2.14 (dd, *J* = 9.6, 1.3 Hz, 1H), 2.38 (s, 3H), 2.39 – 2.44 (m, 1H), 3.33 (s, 1H), 3.39 – 3.44 (m, 1H), 3.83 (dd, *J* = 10.8, 2.8 Hz, 1H), 6.18 – 6.25 (m, 1H), 6.72 – 6.76 (m, 1H), 7.11 (d, *J* = 7.6 Hz, 2H), 7.23 (s, 2H) ppm; ^{13}C NMR (100 MHz, $CDCl_3$): (major isomer) δ = 21.0, 47.5, 48.9 (d, $^3J_{CF}$ = 1.0 Hz), 53.5 (d, $^2J_{CF}$ = 20.5 Hz), 55.65 (d, $^2J_{CF}$ = 17.8 Hz), 125.5 (d, $^1J_{CF}$ = 252.0 Hz), 129.1, 129.3, 132.0 (d, $^3J_{CF}$ = 3.5 Hz), 132.3 (d, $^3J_{CF}$ = 4.4 Hz), 137.4, 140.3 (d, $^4J_{CF}$ = 1.1 Hz) ppm; (minor isomer) δ = 21.1, 46.4, 47.0, 51.5 (d, $^2J_{CF}$ = 22.5 Hz), 52.5 (d, $^2J_{CF}$ = 19.2 Hz), 125.7 (d, $^1J_{CF}$ = 252.7 Hz), 128.4, 129.1, 132.1 (d, $^3J_{CF}$ = 7.3 Hz), 132.5 (d, $^3J_{CF}$ = 6.2 Hz), 137.3, 143.3 (d, $^4J_{CF}$ = 2.3 Hz) ppm. ^{19}F NMR (376 MHz, $CDCl_3$): (major isomer) δ = -128.64 (s) ppm; (minor isomer) δ = -123.79 – - 123.79 (m) ppm.

(1*R,4*S**,5*R**,6*R**)-6-(4-(*tert*-Butyl)phenyl)-5-fluoro-5-nitrobicyclo[2.2.1]hept-2-ene** (*endo*-**2c**; major isomer) and **(1*R**,4*S**,5*S**,6*S**)-6-(4-(*tert*-butyl)phenyl)-5-fluoro-5-nitrobicyclo[2.2.1]hept-2-ene** (*exo*-**2c**; minor isomer). Eluent: Hex/DCM 4:1, Hex/DCM 2:1; 0.114 g (88 %); *dr* = 48:52; yellowish oil. Anal. calcd for $C_{17}H_{20}FNO_2$ (%): C, 70.57; H, 6.97; N, 4.84, Found: C, 70.37; H, 7.17; N, 4.85. ^1H NMR (400 MHz, $CDCl_3$): (major isomer) δ = 1.32 (s, 9H), 1.89 – 1.97 (m, 1H), 2.38 (dd, *J* = 9.6, 0.9 Hz, 1H), 3.35 (s, 1H), 3.50 – 3.54 (m, 1H), 4.18 (dd, *J* = 9.5, 3.0 Hz, 1H), 6.30 (dd, *J* = 5.5, 3.4 Hz, 1H), 6.76 (dd, *J* = 5.6, 2.9 Hz, 1H), 7.07 – 7.14 (m, 2H), 7.31 – 7.37 (m, 2H) ppm; (minor isomer) δ = 1.34 (s, 9H), 2.11 (dd, *J* = 9.6, 1.4 Hz, 1H), 2.42 – 2.49 (m, 1H), 3.31 (s, 1H), 3.37 – 3.42 (m, 1H),), 3.79 (dd, *J* = 11.0, 2.8 Hz, 1H), 6.16 – 6.22 (m, 1H), 6.68 – 6.74 (m, 1H), 7.23 (d, *J* = 8.3 Hz, 2H), 7.37 – 7.43 (m, 2H) ppm; ^{13}C NMR (100 MHz, $CDCl_3$): (major isomer) δ = 31.4, 34.5, 47.7, 49.0 (d, $^3J_{CF}$ = 1.0 Hz), 53.7 (d, $^2J_{CF}$ = 20.5 Hz), 55.7 (d, $^2J_{CF}$ = 18.0 Hz), 125.3, 125.6 (d, $^1J_{CF}$ = 252.0 Hz), 128.3, 132.1 (d, $^3J_{CF}$ = 3.4 Hz), 132.4 (d, $^3J_{CF}$ = 4.4 Hz), 140.4 (d, $^4J_{CF}$ = 1.1 Hz), 150.6 ppm; (minor isomer) δ = 31.4, 34.6, 46.6, 48.0, 51.6 (d, $^2J_{CF}$ = 22.4 Hz), 52.6 (d, $^2J_{CF}$ = 19.4 Hz), 125.6, 125.8 (d, $^1J_{CF}$ = 253.0 Hz), 129.0 (d, $^4J_{CF}$ = 1.0 Hz), 132.1 (d, $^3J_{CF}$ = 7.3 Hz), 132.6 (d, $^3J_{CF}$ = 6.1 Hz), 143.4 (d, $^4J_{CF}$ = 2.3 Hz), 150.6 ppm. ^{19}F NMR (376 MHz, $CDCl_3$): (major isomer) δ = -128.55 (s) ppm; (minor isomer) δ = -123.90 – - 123.81 (m) ppm.

(1*R,4*S**,5*R**,6*R**)-6-(4-Chlorophenyl)-5-fluoro-5-nitrobicyclo[2.2.1]hept-2-ene** (*endo*-**2d**; major isomer) and **(1*R**,4*S**,5*S**,6*S**)-6-(4-chlorophenyl)-5-fluoro-5-nitrobicyclo[2.2.1]hept-2-ene** (*exo*-**2d**; minor isomer). Eluent: Hex/DCM 4:1, Hex/DCM 2:1; 0.123 g (90 %) [scale-up: 1.057 g (83 %)], *dr* = 41:59; colorless oil. Anal. calcd for $C_{13}H_{11}ClFNO_2$ (%): C, 58.33; H, 4.14; N, 5.23, Found: C, 58.61; H, 4.09; N, 5.31. ^1H NMR (400 MHz, $CDCl_3$): (major isomer) δ = 1.89 – 1.99 (m, 1H), 2.35 (dd, *J* = 9.8,

0.9 Hz, 1H), 3.32 (s, 1H), 3.53 (s, 1H), 4.14 (dd, J = 9.3, 3.1 Hz, 1H), 6.30 (dd, J = 5.5, 3.4 Hz, 1H), 6.66 – 6.73 (m, 1H), 7.09 (d, J = 7.2 Hz, 2H), 7.24 – 7.30 (m, 2H) ppm; (minor isomer) δ = 2.11 (dd, J = 9.7, 1.4 Hz, 1H), 2.34- 2.40 (m, 1H), 3.28 (s, 1H), 3.36 – 3.43 (m, 1H), 3.76 (dd, J = 10.7, 2.8 Hz, 1H), 6.15 – 6.22 (m, 1H), 6.66 – 6.74 (m, 1H), 7.22 (d, J = 8.5 Hz, 2H), 7.30 – 7.36 (m, 2H) ppm; ^{13}C NMR (100 MHz, $CDCl_3$): (major isomer) δ = 47.6, 48.9 (d, $^3J_{CF}$ = 1.2 Hz), 53.5 (d, $^2J_{CF}$ = 20.4 Hz), 55.3 (d, $^2J_{CF}$ = 17.7 Hz), 125.3 (d, $^1J_{CF}$ = 252.2 Hz), 128.5, 130.6 (d, $^4J_{CF}$ = 1.3 Hz), 132.8 (d, $^3J_{CF}$ = 4.5 Hz), 133.6 (d, $^3J_{CF}$ = 3.1 Hz), 133.7, 140.0 (d, $^4J_{CF}$ = 1.1 Hz); (minor isomer) δ = 46.3, 47.7, 51.4 (d, $^2J_{CF}$ = 22.3 Hz), 52.2 (d, $^2J_{CF}$ = 19.0 Hz), 125.6 (d, $^1J_{CF}$ = 252.8 Hz), 128.79, 130.0, 132.8 (d, $^3J_{CF}$ = 6.0 Hz), 133.6, 133.7 (d, $^3J_{CF}$ = 7.3 Hz), 143.2 (d, $^4J_{CF}$ = 2.2 Hz) ppm; ^{19}F NMR (376 MHz, $CDCl_3$): (major isomer) δ = -128.63 (s) ppm; (minor isomer) δ = -123.69 – -123.53 (m) ppm.

(1*R,4*S**,5*R**,6*R**)-6-(2,4-Dichlorophenyl)-5-fluoro-5-nitrobicyclo[2.2.1]hept-2-ene** (*endo*-**2e**; major isomer) and **(1*R**,4*S**,5*S**,6*S**)-6-(2,4-dichlorophenyl)-5-fluoro-5-nitrobicyclo[2.2.1]hept-2-ene** (*exo*-**2e**; minor isomer). Eluent: Hex/DCM 4:1, Hex/DCM 1:1; 0.125 g (97 %), *dr* = 46:54; yellowish solid; M.p. 93-94 °C. Anal. calcd for $C_{13}H_{10}Cl_2FNO_2$ (%): C, 51.68; H, 3.34; N, 4.64; found: C, 51.97; H, 3.59; N, 4.71. ^{1}H NMR (400 MHz, $CDCl_3$): (major isomer) δ = 1.93 (dd, J = 9.4, 5.1 Hz, 1H), 2.44 (d, J = 9.6 Hz, 1H), 3.31 (s, 1H), 3.45 (s, 1H), 4.78 (dd, J = 10.0, 2.8 Hz, 1H), 6.32 (dd, J = 8.7, 3.5 Hz, 1H), 6.74 (dd, J = 5.4, 2.8 Hz, 1H), 7.09 – 7.22 (m, 1H), 7.28 (s, 1H), 7.32 – 7.42 (m, 1H) ppm; (minor isomer) δ = 2.10 (d, J = 9.4 Hz, 1H), 2.34 (d, J = 9.3 Hz, 1H), 3.25 (s, 1H), 3.36 (s, 1H), 4.10 (dd, J = 10.3, 2.5 Hz, 1H), 6.16 (s, 1H), 6.70 (s, 1H), 7.09 – 7.22 (m, 1H), 7.28 (s, 1H), 7.32 – 7.42 (m, 1H) ppm; ^{13}C NMR (100 MHz, $CDCl_3$): (major isomer) δ = 47.5, 49.2 (d, $^3J_{CF}$ = 1.1 Hz), 51.5 (d, $^2J_{CF}$ = 17.4 Hz), 54.4 (d, $^2J_{CF}$ = 20.2 Hz), 125.0 (d, $^1J_{CF}$ = 254.1 Hz), 127.0, 129.4, 130.6 (d, $^3J_{CF}$ = 3.8 Hz), 131.95 (d, $^3J_{CF}$ = 2.6 Hz), 132.9, 132.9, 136.2, 140.1 ppm; (minor isomer) δ = 46.8, 48.4, 50.4 (d, $^2J_{CF}$ = 18.6 Hz), 53.0 (d, $^2J_{CF}$ = 22.2 Hz), 123.3 (d, $^1J_{CF}$ = 256.7 Hz), 127.4, 129.0, 129.5, 132.9, 132.9 (d, $^3J_{CF}$ = 5.3 Hz), 134.2, 136.4, 141.7 (d, $^4J_{CF}$ = 2.5 Hz) ppm; ^{19}F NMR (376 MHz, $CDCl_3$): (major isomer) δ = -128.84 (s) ppm; (minor isomer) δ = -126.50 (s) ppm.

(1*R,4*S**,5*R**,6*R**)-6-(4-Bromophenyl)-5-fluoro-5-nitrobicyclo[2.2.1]hept-2-ene** (*endo*-**2f**; major isomer) and **(1*R**,4*S**,5*S**,6*S**)-6-(4-bromophenyl)-5-fluoro-5-nitrobicyclo[2.2.1]hept-2-ene** (*exo*-**2f**; minor isomer). Eluent: Hex/DCM 4:1, Hex/DCM 2:1; 0.115 g (90 %) [scale-up: 0.926 g (73 %)]; *dr* = 43:57; yellowish oil. Anal. calcd for $C_{13}H_{11}BrFNO_2$ (%): C, 50.02; H, 3.55; N, 4.49; found: C, 50.02; H, 3.67; N, 4.40. ^{1}H NMR (400 MHz, $CDCl_3$): (major isomer) δ = 1.88 – 1.98 (m, 1H), 2.34 (d, J = 9.8 Hz, 1H), 3.32 (s, 1H), 3.52 (s, 1H), 4.12 (dd, J = 9.3, 3.0 Hz, 1H), 6.30 (dd, J = 5.4, 3.5 Hz, 1H), 6.64 – 6.74 (m, 1H), 7.03 (d, J = 7.6 Hz, 2H), 7.39 – 7.45 (m, 2H) ppm; (minor isomer) δ = 2.10 (dd, J = 9.7, 1.3 Hz, 1H), 2.36 (d, J = 9.8 Hz, 1H), 3.27 (s, 1H), 3.39 (s, 1H), 3.75 (dd, J = 10.7, 2.7 Hz, 1H), 6.15 – 6.21 (m, 1H), 6.64 – 6.74 (m, 1H), 7.16 (d, J = 8.4 Hz, 2H), 7.44 – 7.52 (m, 2H) ppm; ^{13}C NMR (100 MHz, $CDCl_3$):

(major isomer) δ = 47.6, 48.9 (d, $^3J_{CF}$ = 1.0 Hz), 53.5 (d, $^2J_{CF}$ = 20.4 Hz), 55.3 (d, $^2J_{CF}$ = 17.7 Hz), 121.9, 125.3 (d, $^1J_{CF}$ = 252.2 Hz), 131.0 (d, $^4J_{CF}$ = 1.2 Hz), 131.6, 132.86 (d, $^3J_{CF}$ = 4.2 Hz), 134.1 (d, $^3J_{CF}$ = 3.4 Hz), 140.0 ppm; (minor isomer) δ = 46.3, 47.8, 51.5 (d, $^2J_{CF}$ = 22.2 Hz), 52.3 (d, $^2J_{CF}$ = 19.1 Hz), 121.8, 125.6 (d, $^1J_{CF}$ = 253.2 Hz), 130.4, 131.8, 132.9 (d, $^3J_{CF}$ = 6.3 Hz), 134.3 (d, $^3J_{CF}$ = 7.2 Hz), 143.2 (d, $^4J_{CF}$ = 2.3 Hz) ppm; ^{19}F NMR (376 MHz, $CDCl_3$): (major isomer) δ = -128.60 (s) ppm; (minor isomer) -123.57 (s) ppm.

(1*R,4*S**,5*R**,6*R**)-5-Fluoro-6-(4-methoxyphenyl)-5-nitrobicyclo[2.2.1]hept-2-ene** (*endo*-**2g**; major isomer) and **(1*R**,4*S**,5*S**,6*S**)-5-fluoro-6-(4-methoxyphenyl)-5-nitrobicyclo[2.2.1]hept-2-ene** (*exo*-**2g**; minor isomer). Eluent: Hex/DCM 10:1, Hex/DCM 5:1; 0.095 g (68 %); *dr* = 45:55; pale brown oil. Anal. calcd for $C_{14}H_{14}FNO_3$ (%): C, 63.87; H, 5.36; N, 5.32; Found: C, 63.92; H, 5.40; N, 5.27. ^{1}H NMR (400 MHz, $CDCl_3$): (major isomer) δ = 1.88 –1.96 (m, 1H), 2.36 (dd, *J* = 9.6, 0.9 Hz, 1H), 3.30 (s, 1H), 3.51 (s, 1H), 3.78 (s, 3H), 4.12 (dd, *J* = 9.5, 3.0 Hz, 1H), 6.29 (dd, *J* = 5.4, 3.4 Hz, 1H), 6.67 – 6.75 (m, 1H), 6.81 – 6.87 (m, 2H), 7.09 (dd, *J* = 8.8, 0.7 Hz, 2H) ppm; (minor isomer) δ = 2.09 (dd, *J* = 9.6, 1.3 Hz, 1H), 2.42 (d, *J* = 9.5 Hz, 1H), 3.26 (s, 1H), 3.43 – 3.41 (m, 1H), 3.74 (dd, *J* = 10.5, 2.3 Hz, 1H), 3.81 (s, 3H), 6.14 – 6.22 (m, 1H), 6.67 – 6.75 (m, 1H), 6.81 – 6.87 (m, 2H), 7.20 (d, *J* = 8.6 Hz, 2H) ppm; ^{13}C NMR (100 MHz, $CDCl_3$): (major isomer) δ = 47.8, 49.0 (d, $^3J_{CF}$ = 1.2 Hz), 53.6 (d, $^2J_{CF}$ = 20.5 Hz), 55.3, 55.4 (d, $^2J_{CF}$ = 18.1 Hz), 113.8, 125.5 (d, $^1J_{CF}$ = 251.6 Hz), 127.1 (d, $^3J_{CF}$ = 4.0 Hz), 129.7, 132.5 (d, $^3J_{CF}$ = 4.4 Hz), 140.32 (d, $^4J_{CF}$ = 1.2 Hz), 159.1 ppm; (minor isomer) δ = 46.7, 47.9, 51.5 (d, $^2J_{CF}$ = 22.5 Hz), 52.4 (d, $^2J_{CF}$ = 19.3 Hz), , 55.3, 114.1, 125.6 (d, $^1J_{CF}$ = 252.6 Hz), 127.1 (d, $^3J_{CF}$ = 7.0 Hz), , 130.4 (d, $^4J_{CF}$ = 1.0 Hz), 132.6 (d, $^3J_{CF}$ = 6.2 Hz), 143.4 (d, $^4J_{CF}$ = 2.3 Hz), 159.2 ppm; ^{19}F NMR (376 MHz, $CDCl_3$): (major isomer) δ = -128.61 (s) ppm, (minor isomer) δ = -123.8 (s) ppm.

Methyl 4-((1*R,2*R**,3*R**,4*S**)-3-fluoro-3-nitrobicyclo[2.2.1]hept-5-en-2-yl)benzoate** (*endo*-**2h**; major isomer) and **methyl 4-((1*R**,2*S**,3*S**,4*S**)-3-fluoro-3-nitrobicyclo[2.2.1]hept-5-en-2-yl)benzoate** (*exo*-**2h**; minor isomer). Eluent: Hex/DCM 2:1, Hex/DCM 1:1, Hex/DCM 1:2; 0.127 g (95 %), *dr* = 45:55; pale yellow solid; M.p. 72-75 °C. Anal. calcd for $C_{15}H_{14}FNO_4$ (%): C, 61.85; H, 4.84; N, 4.81, Found: C, 62.11; H, 4.90; N, 4.94. ^{1}H NMR (400 MHz, $CDCl_3$): (major isomer) δ = 1.88 – 1.97 (m, 1H), 2.33 (dd, *J* = 9.7, 1.0 Hz, 1H), 3.35 (br s, 1H), 3.48 – 3.53 (m, 1H), 3.87 (s, 3H), 4.21 (dd, *J* = 9.2, 3.1 Hz, 1H), 6.27 (dd, *J* = 5.6, 3.4 Hz, 1H), 6.69 (dd, *J* = 5.5, 2.9 Hz, 1H), 7.14 – 7.24 (m, 2H), 7.90 – 7.97 (m, 2H) ppm; (minor isomer) δ = 2.07 – 2.14 (m, 1H), 2.35 – 2.41 (m, 1H), 3.32 (br s, 1H), 3.35 – 3.40 (m, 1H), 3.83 (dd, *J* = 10.5, 3.2 Hz, 1H), 3.89 (s, 3H), 6.11 – 6.20 (m, 1H), 6.69 (dd, *J* = 5.5, 2.9 Hz, 1H), 7.31 – 7.36 (m, 2H), 7.97 –8.01 (m, 2H) ppm; ^{13}C NMR (100 MHz, $CDCl_3$): (major isomer) δ = 47.4, 48.8 (d, $^3J_{CF}$ = 1.5 Hz), 52.2, 53.5 (d, $^2J_{CF}$ = 20.4 Hz), 55.6 (d, $^2J_{CF}$ = 17.6 Hz), 125.4 (d, $^1J_{CF}$ = 252.7 Hz), 129.2 (d, $^4J_{CF}$ = 1.8 Hz), 129.4, 129.5, 132.8 (d, $^3J_{CF}$ = 4.5 Hz), 140.0 (d, $^4J_{CF}$ = 1.5 Hz), 140.2 (d, $^3J_{CF}$ = 3.4 Hz), 166.7 ppm; (minor

isomer) δ = 46.1 (d, $^3J_{CF}$ = 0.9 Hz), 47.8 (d, $^3J_{CF}$ = 1.1 Hz), 51.5 (d, $^2J_{CF}$ = 22.2 Hz), 52.2, 52.6 (d, $^2J_{CF}$ = 19.0 Hz), 125.8 (d, $^1J_{CF}$ = 253.4 Hz), 128.6 (d, $^4J_{CF}$ = 0.7 Hz), 129.5, 129.8, 132.8 (d, $^3J_{CF}$ = 6.3 Hz), 140.4 (d, $^3J_{CF}$ = 7.2 Hz), 143.1 (d, $^4J_{CF}$ = 2.4 Hz), 166.7 ppm; ^{19}F NMR (376 MHz, $CDCl_3$): (major isomer) δ = -128.55 (s) ppm; (minor isomer) -123.67 (s) ppm.

(1*R,4*S**,5*R**,6*R**)-5-Fluoro-5-nitro-6-(4-(trifluoromethyl)phenyl)bicyclo[2.2.1]hept-2-ene** (*endo*-**2i**; major isomer) and **(1*R**,4*S**,5*S**,6*S**)-5-fluoro-5-nitro-6-(4-(trifluoromethyl)phenyl)bi-cyclo[2.2.1]hept-2-ene** (*exo*-**2i**; minor isomer). Eluent: Hex/DCM, 5:1, Hex/DCM, 2:1; 0.130 g (80 %); *dr* = 38:62; colorless oil. Anal. calcd for $C_{14}H_{11}F_4NO_2$: C, 55.82; H, 3.68; N, 4.65; found: C, 56.06; H, 3.50; N, 4.49. ^{1}H NMR (400 MHz, $CDCl_3$): (major isomer) δ = 1.92 – 2.03 (m, 1H), 2.31 – 2.46 (m, 1H), 3.38 (s, 1H), 3.56 (s, 1H), 4.24 (dd, *J* = 9.0, 2.1 Hz, 1H), 6.29 – 6.37 (m, 1H), 6.65 – 6.77 (m, 1H), 7.29 (d, *J* = 7.9 Hz, 2H), 7.56 (d, *J* = 8.1 Hz, 2H) ppm; (minor isomer) δ = 2.15 (d, *J* = 9.5 Hz, 1H), 2.31 – 2.46 (m, 1H), 3.34 (s, 1H), 3.42 (s, 1H), 3.86 (dd, *J* = 10.6, 1.6 Hz,1H), 6.17– 6.24 (m, 1H), 6.65 – 6.77 (m, 1H), 7.42 (d, *J* = 8.0 Hz, 2H), 7.62 (d, *J* = 8.0 Hz, 2H) ppm; ^{13}C NMR (100 MHz, $CDCl_3$): (major isomer) δ = 47.6, 48.9 (d, $^3J_{CF}$ = 1.0 Hz), 53.5 (d, $^2J_{CF}$ = 20.3 Hz), 55.5 (d, $^2J_{CF}$ = 17.5 Hz), 124.1 (q, $^1J_{CF}$ = 272.1 Hz), 125.3 (q, $^3J_{CF}$ = 3.6 Hz), 125.4 (d, $^1J_{CF}$ = 252.6 Hz), 129.7 (d, $^4J_{CF}$ = 1.2 Hz), 130.0 (q, $^2J_{CF}$ = 32.0 Hz), 133.0 (d, $^3J_{CF}$ = 4.0 Hz), 139.2, 139.9 (d, $^4J_{CF}$ = 0.9 Hz) ppm; (minor isomer) δ = 46.2, 47.8, 51.5 (d, $^2J_{CF}$ = 22.2 Hz), 52.5 (d, $^2J_{CF}$ = 19.0 Hz), 124.1 (q, $^1J_{CF}$ = 272.1 Hz), 125.6 (q, $^3J_{CF}$ = 3.6 Hz), 125.8 (d, $^1J_{CF}$ = 253.4 Hz), 129.1, 130.0 (q, $^2J_{CF}$ = 32.0 Hz), 133.0 (d, $^3J_{CF}$ = 5.2 Hz), 139.4 (d, $^3J_{CF}$ = 7.9 Hz), 143.2 (d, $^4J_{CF}$ = 2.2 Hz) ppm; ^{19}F NMR (376 MHz, $CDCl_3$): (major isomer) δ = -128.41 (s, 1F), -63.70 (s, 3F) ppm; (minor isomer) δ = 123.38 (s, 1F), -63.65 (s, 3F) ppm.

4-((1*R,2*R**,3*R**,4*S**)-3-Fluoro-3-nitrobicyclo[2.2.1]hept-5-en-2-yl)benzonitrile** (*endo*-**2j**; major isomer) and **4-((1*R**,2*S**,3*S**,4*S**)-3-fluoro-3-nitrobicyclo[2.2.1]hept-5-en-2-yl)benzonitrile** (*exo*-**2j**; minor isomer). Eluent: Hex/DCM, 1:1; Hex/DCM, 1:2; 0.101 g (75 %); *dr* = 34:66; colorless oil. Anal. calcd for $C_{14}H_{11}FN_2O_2$ (%): C, 65.11; H, 4.29; N, 10.85; Found: C, 65.38; H, 4.32; N, 10.51. ^{1}H NMR (400 MHz, $CDCl_3$): (major isomer) δ = 1.91 – 2.01 (m, 1H), 2.32 (s, 1H), 3.37 (s, 1H), 3.55 (s, 1H), 4.21 (dd, *J* = 9.1, 3.0 Hz, 1H), 6.31 (dd, *J* = 5.4, 3.4 Hz, 1H), 6.66 – 6.73 (m, 1H), 7.25 – 7.30 (m, 2H), 7.61 – 7.66 (m, 2H) ppm; (minor isomer) δ = 2.13 (dd, *J* = 9.8, 1.4 Hz, 1H), 2.35 (s, 1H), 3.33 (s, 1H), 3.39 – 3.43 (m, 1H), 3.83 (dd, *J* = 10.7, 2.8 Hz, 1H), 6.16 – 6.21 (m, 1H), 6.66 – 6.73 (m, 1H), 7.41 (d, *J* = 8.2 Hz, 2H), 7.60 – 7.67 (m, 2H) ppm. ^{13}C NMR (100 MHz, $CDCl_3$): (major isomer) δ = 47.4, 48.8 (d, $^3J_{CF}$ = 1.1 Hz), 53.3 (d, $^2J_{CF}$ = 20.3 Hz), 55.5 (d, $^2J_{CF}$ = 17.4 Hz), 111.6, 118.6, 125.2 (d, $^1J_{CF}$ = 252.7 Hz), 130.0 (d, $^4J_{CF}$ = 1.5 Hz), 132.1, 133.1 (d, $^3J_{CF}$ = 4.4 Hz), 139.7 (d, $^4J_{CF}$ = 1.0 Hz), 140.5 (d, $^3J_{CF}$ = 3.1 Hz) ppm; (minor isomer) δ = 45.9, 47.6, 51.28 (d, $^2J_{CF}$ = 22.1 Hz), 52.53 (d, $^2J_{CF}$ = 18.9 Hz), 111.6, 118.6, 125.74 (d, $^1J_{CF}$ = 253.2 Hz), 129.45, 132.32, 133.0 (d, $^3J_{CF}$ = 6.2 Hz), 140.67 (d, $^3J_{CF}$ = 7.4 Hz), 143.01 (d, $^4J_{CF}$ = 2.2 Hz) ppm; ^{19}F NMR (376 MHz, $CDCl_3$): (major isomer) δ = -128.48 (s) ppm ; (minor isomer) δ = -123.31 (s) ppm.

(1*R,4*S**,5*R**,6*R**)-5-Fluoro-5-nitro-6-(3-nitrophenyl)bicyclo[2.2.1]hept-2-ene** (*endo*-**2k**; major isomer) and **(1*R**,4*S**,5*S**,6*S**)-5-fluoro-5-nitro-6-(3-nitrophenyl)bicyclo[2.2.1]hept-2-ene** (*exo*-**2k**; minor isomer). Eluent: Hex/DCM, 3:1, Hex/DCM, 2:1; 0.112 g (80%); *dr* = 31:69; greenish oil. Anal. calcd for $C_{13}H_{11}FN_2O_4$ (%): C, 56.12; H, 3.98; N, 10.07; Found: C, 56.33; H, 3.99; N, 9.95; ^{1}H NMR (400 MHz, $CDCl_3$): (major isomer) δ = 1.93 – 2.06 (m, 1H), 2.36 (dd, *J* = 9.8, 1.0 Hz, 1H), 3.41 (s, 1H), 3.58 (s, 1H), 4.27 (dd, *J* = 9.1, 3.1 Hz, 1H), 6.37 (dd, *J* = 5.5, 3.4 Hz, 1H), 6.69 – 6.78 (m, 1H), 7.44 – 7.52 (m, 2H), 8.04 (s, 1H), 8.09 – 8.19 (m, 1H) ppm; (minor isomer) δ = 2.18 (dd, *J* = 9.9, 1.3 Hz, 1H), 2.40 (dd, *J* = 9.9, 1.2 Hz, 1H), 3.38 (s, 1H), 3.45 (dd, *J* = 8.5, 6.7 Hz, 1H), 3.89 (dd, *J* = 10.6, 2.7 Hz, 1H), 6.15 – 6.27 (m, 1H), 6.69 – 6.78 (m, 1H), 7.44 – 7.57 (m, 1H), 7.65 (d, *J* = 7.8 Hz, 1H), 8.09 – 8.19 (m, 2H) ppm; ^{13}C NMR (100 MHz, $CDCl_3$): (major isomer) δ = 47.6, 48.9 (d, $^3J_{CF}$ = 1.5 Hz), 53.4 (d, $^2J_{CF}$ = 20.3 Hz), 55.2 (d, $^2J_{CF}$ = 17.4 Hz), 122.8, 124.0 (d, $^4J_{CF}$ = 1.8 Hz), 125.1 (d, $^1J_{CF}$ = 252.5 Hz), 129.4, 133.4 (d, $^3J_{CF}$ = 4.3 Hz), 135.8 (d, $^3J_{CF}$ = 1.8 Hz), 137.2 (d, $^3J_{CF}$ = 3.4 Hz), 139.6 (d, $^4J_{CF}$ = 1.5 Hz), 148.3 ppm; (minor isomer) δ = 46.1 (d, $^3J_{CF}$ = 0.6 Hz), 47.6, 51.3 (d, $^2J_{CF}$ = 22.1 Hz), 52.2 (d, $^2J_{CF}$ = 18.8 Hz), 122.9, 123.0 (d, $^4J_{CF}$ = 0.6 Hz), 125.7 (d, $^1J_{CF}$ = 253.1 Hz), 129.6, 133.1 (d, $^3J_{CF}$ = 6.2 Hz), 135.5 (d, $^3J_{CF}$ = 0.5 Hz), 137.4 (d, $^3J_{CF}$ = 7.5 Hz), 143.0 (d, $^4J_{CF}$ = 2.3 Hz), 148.5 ppm; ^{19}F NMR (376 MHz, $CDCl_3$): (major isomer) δ = -128.40 (s) ppm; (minor isomer) δ = -123.02 (s) ppm.

(1*R,4*S**,5*R**,6*R**)-5-Fluoro-5-nitro-6-(4-nitrophenyl)bicyclo[2.2.1]hept-2-ene** (*endo*-**2l**; major isomer) and **(1*R**,4*S**,5*S**,6*S**)-5-fluoro-5-nitro-6-(4-nitrophenyl)bicyclo[2.2.1]hept-2-ene** (*exo*-**2l**; minor isomer). Eluent: Hex/DCM, 3:1; Hex/DCM, 2:1; 0.089 g (67 %) [scale-up: 0.705 g (71 %)]; *dr* = 40:60; brown oil. Anal. calcd for $C_{13}H_{11}FN_2O_4$ (%): C, 56.12; H, 3.98; N, 10.07, Found: C, 56.36; H, 4.18; N, 9.95. ^{1}H NMR (400 MHz, $CDCl_3$): (major isomer) δ = 1.96 – 2.03 (m, 1H), 2.32 – 2.41 (m, 1H), 3.38 – 3.42 (m, 1H), 3.56 – 3.59 (m, 1H), 4.27 (dd, *J* = 9.1, 3.1 Hz, 1H), 6.34 (dd, *J* = 5.6, 3.4 Hz, 1H), 6.70 (dd, *J* = 5.7, 2.8 Hz, 1H), 7.32 – 7.37 (m, 2H), 8.10 – 8.16 (m, 2H) ppm; (minor isomer) δ = 2.17 (dtd, *J* = 9.8, 2.8, 1.5 Hz, 1H), 2.32 – 2.41 (m, 1H), 3.34 – 3.39 (m, 1H), 3.41 – 3.46 (m, 1H), 3.89 (dd, *J* = 10.7, 2.9 Hz, 1H), 6.18 – 6.23 (m, 1H), 6.71 – 6.75 (m, 1H), 7.44 – 7.51 (m, 2H), 8.16 – 8.22 (m, 2H) ppm; ^{13}C NMR (100 MHz, $CDCl_3$): (major isomer) δ = 47.6, 48.9 (d, $^3J_{CF}$ = 1.5 Hz), 53.4 (d, $^2J_{CF}$ = 20.2 Hz), 55.3 (d, $^2J_{CF}$ = 17.3 Hz), 123.5, 125.2 (d, $^1J_{CF}$ = 252.8 Hz), 130.2 (d, $^4J_{CF}$ = 1.9 Hz), 133.3 (d, $^3J_{CF}$ = 4.3 Hz), 139.7 (d, $^4J_{CF}$ = 1.6 Hz), 142.5 (d, $^3J_{CF}$ = 3.2 Hz), 147.3 ppm; (minor isomer) δ = 46.1 (d, $^3J_{CF}$ = 0.6 Hz), 47.6, 51.3 (d, $^2J_{CF}$ = 21.9 Hz), 52.4 (d, $^2J_{CF}$ = 18.7 Hz), 123.7, 125.8 (d, $^1J_{CF}$ = 253.6 Hz), 129.6 (d, $^4J_{CF}$ = 0.6 Hz), 133.1 (d, $^3J_{CF}$ = 6.2 Hz), 142.7 (d, $^3J_{CF}$ = 7.3 Hz), 143.0 (d, $^4J_{CF}$ = 2.4 Hz), 147.4 ppm; ^{19}F NMR (376 MHz, $CDCl_3$): (major isomer) δ = -128.39 (s) ppm; (minor isomer) δ = -123.23 (s) ppm.

Methyl 4-((1*R,4*S**,5*S**,6*S**)-5-Fluoro-5-nitrospiro[bicyclo[2.2.1]heptane-7,1'-cyclopropan]-2-en-6-yl)benzoate** (*exo*-**2m**; major isomer) and **methyl 4-((1*R**,4*S**,5*R**,6*R**)-5-fluoro-5-nitrospiro[bicyclo[2.2.1]heptane-7,1'-cyclopropan]-2-en-6-yl)benzoate** (*endo*-**2m**; minor isomer).

Eluent: Hex/DCM, 2:1; Hex/DCM, 1:1; Hex/DCM, 1:3; 0.070 g (44 %); *dr* = 44:56; yellowish oil. Anal. calcd for $C_{17}H_{16}FNO_4$ (%): C, 64.35; H, 5.08; N, 4.41; found: C, 64.18; H, 5.18; N, 4.24. ^{1}H NMR (400 MHz, $CDCl_3$): (major isomer) δ = 0.53 – 0.68 (m, 2H), 0.71 – 0.81 (m, 1H), 0.96 (dd, *J* = 14.8, 7.6 Hz, 1H), 2.84 – 2.89 (m, 1H), 3.00 (br s, 1H), 3.90 (s, 3H), 4.04 (d, *J* = 11.8 Hz, 1H), 6.21 – 6.27 (m, 1H), 6.81 (dd, *J* = 5.8, 3.2 Hz, 1H), 7.37 (d, *J* = 8.3 Hz, 2H), δ 7.99 (d, *J* = 8.4 Hz, 2H) ppm; (minor isomer) δ = 0.53 – 0.68 (m, 2H), 0.71 – 0.81 (m, 1H), 1.01 – 1.11 (m, 1H), 2.68 – 2.74 (m, 1H), 3.18 (br s, 1H), 3.89 (s, 3H), 4.58 (dd, *J* = 9.6, 3.2 Hz, 1H), 6.40 (dd, *J* = 5.5, 3.5 Hz, 1H), 6.77 (dd, *J* = 5.7, 2.8 Hz, 1H), 7.31 (d, *J* = 7.5 Hz, 2H), 7.96 (d, *J* = 8.4 Hz, 2H) ppm; ^{13}C NMR (100 MHz, $CDCl_3$): (major isomer) δ = 10.2, 11.3 (d, $^4J_{CF}$ = 2.9 Hz), 44.8, 50.6, 52.2, 54.7 (d, $^2J_{CF}$ = 18.3 Hz), 57.3 (d, $^2J_{CF}$ = 21.0 Hz), 125.5 (d, $^1J_{CF}$ = 256.3 Hz), 129.3, 129.5 (d, $^4J_{CF}$ = 1.1 Hz), 129.6, 132.0 (d, $^3J_{CF}$ = 5.8 Hz), 140.1, 140.1 (d, $^3J_{CF}$ = 6.4 Hz), 166.8 ppm; (minor isomer) δ = 3.8, 5.4, 44.4 (d, $^3J_{CF}$ = 3.5 Hz), 52.2, 53.6, 55.0 (d, $^2J_{CF}$ = 17.7 Hz), 56.0 (d, $^2J_{CF}$ = 20.4 Hz), 126.4 (d, $^1J_{CF}$ = 252.0 Hz), 129.3, 129.4, 129.6, 132.2 (d, $^3J_{CF}$ = 3.1 Hz), 140.3 (d, $^3J_{CF}$ = 3.5 Hz), 143.5 (d, $^4J_{CF}$ = 1.5 Hz), 166.9 ppm; ^{19}F NMR (376 MHz, $CDCl_3$): (major isomer) δ = -127.47 (dd, *J* = 10.8, 5.7 Hz) ppm; (minor isomer) δ = -124.77 (d, *J* = 9.3 Hz) ppm;

(1*R,4*S**,5*S**,6*S**)-6-(2,4-Dichlorophenyl)-5-fluoro-5-nitrobicyclo[2.2.2]oct-2-ene** (*exo*-**3a**; major isomer) and **(1*R**,4*S**,5*R**,6*R**)-6-(2,4-dichlorophenyl)-5-fluoro-5-nitrobicyclo[2.2.2]oct-2-ene** (*endo*-**3a**; minor isomer). Eluent: Hex/DCM, 3:1; 0.051 g (32 %); [scale-up under microwave activation: 0.119 g (35 %)]; *dr* = 41:59; viscous pale yellow oil. Anal. calcd for $C_{14}H_{12}Cl_2FNO_2$: C, 53.19; H, 3.83; N, 4.43; found: C, 53.45; H, 3.87: N, 4.25; ^{1}H NMR (400 MHz, $CDCl_3$): (major isomer) δ = 1.32 – 1.62 (m, 2H), 1.88 –2.06 (m, 2H), 2.71 – 2.80 (m, 1H), 3.27 – 3.38 (m, 1H), 4.43 (dt, *J* = 14.4, 1.8 Hz, 1H), 6.22 – 6.29 (m, 1H), 6.67 (t, *J* = 7.4 Hz, 1H), 7.22 – 7.51 (m, 3H) ppm; (minor isomer) δ = 1.20 – 1.33 (m, 2H), 2.11 – 2.24 (m, 2H), 2.93 – 3.01 (m, 1H), 3.14 – 3.21 (m, 1H), 4.68 (dd, *J* = 12.4, 1.3 Hz, 1H), 6.29 – 6.35 (m, 1H), 6.71 (t, *J* = 7.3 Hz, 1H), 7.15 – 7.21 (m, 1H), 7.22 – 7.51 (m, 2H) ppm; ^{13}C NMR (100 MHz, $CDCl_3$): (major isomer) δ = 17.2, 19.8 (d, $^3J_{CF}$ = 3.9 Hz), 36.3, 39.4 (d, $^2J_{CF}$ = 21.9 Hz), 44.2 (d, $^2J_{CF}$ = 16.5 Hz), 124.0 (d, $^1J_{CF}$ = 247.6 Hz), 127.1, 128.99, 129.92, 130.62 (d, $^4J_{CF}$ = 2.2 Hz), 131.41, 134.09 (d, $^3J_{CF}$ = 11.6 Hz), 136.13, 137.89 ppm; ^{13}C NMR (100 MHz, $CDCl_3$): (minor isomer) δ = 19.99 (d, $^3J_{CF}$ = 4.3 Hz), 24.40, 36.58, 40.41 (d, $^2J_{CF}$ = 21.4 Hz), 48.85 (d, $^2J_{CF}$ = 17.6 Hz), 122.32 (d, $^1J_{CF}$ = 247.8 Hz), 126.88, 128.94 (d, $^3J_{CF}$ = 4.1 Hz), 129.35, 131.36 (d, $^4J_{CF}$ = 3.2 Hz), 131.51, 132.93 (d, $^3J_{CF}$ = 7.6 Hz), 134.75, 135.69 ppm; ^{19}F NMR (376 MHz, $CDCl_3$): (major isomer) δ = -128.89 (dd, *J* = 14.4, 2.1 Hz) ppm; (minor isomer) δ = -117.92 – -117.79 (m) ppm

Methyl 4-((1*R,2*S**,3*S**,4*S**)-3-fluoro-3-nitrobicyclo[2.2.2]oct-5-en-2-yl)benzoate** (*exo*-**3b**; major isomer) and **methyl 4-((1*R**,2*R**,3*R**,4*S**)-3-fluoro-3-nitrobicyclo[2.2.2]oct-5-en-2-yl)benzoate** (*endo*-**3b**; minor isomer). Eluent: Hex/DCM, 1:1; Hex/DCM, 2:3; Hex/DCM, 1:3; Hex/DCM, 1:5; Hex/DCM, 1:10; DCM; 0.042 g (28 %); *dr* = 33:67; yellowish oil. Anal. calcd for $C_{16}H_{16}FNO_4$ (%): C, 62.95; H, 5.28; N, 4.59; Found: C, 63.14; H, 5.27; N, 4.59. ^{1}H NMR (400 MHz, $CDCl_3$): (major isomer) δ = 1.40 – 1.55

(m, 2H), 1.98 – 2.25 (m, 2H), 3.03 – 3.10 (m, 1H), 3.25 – 3.34 (m, 1H), 3.88 (d, $^3J_{HF}$ = 15.8 Hz, 1H), 3.92 (s, 3H), 6.25 – 6.33 (m, 1H), 6.66 (t, J = 7.4 Hz, 1H), 7.27 – 7.38 (m, 2H), 7.97 – 8.07 (m, 2H) ppm; (minor isomer) δ = 1.30 – 1.41 (m, 2H), 1.77 – 1.97 (m, 2H), 3.02 (dd, J = 2.9, 1.6 Hz, 1H), 3.31 – 3.36 (m, 1H), 3.90 (s, 3H), 4.33 (d, $^3J_{HF}$ = 13.6 Hz, 1H), 6.29 – 6.36 (m, 1H), 6.75 (t, J = 7.2 Hz, 1H), 7.24 (d, J = 8.2 Hz, 2H), 7.91 – 7.98 (m, 2H) ppm; ^{13}C NMR (100 MHz, $CDCl_3$): (major isomer) δ = 17.2, 20.5 (d, $^3J_{CF}$ = 5.2 Hz), 35.7, 39.7 (d, $^2J_{CF}$ = 22.2 Hz), 49.6 (d, $^2J_{CF}$ = 18.7 Hz), 52.3, 121.6 (d, $^1J_{CF}$ = 250.9 Hz), 129.0, 129.4 (d, $^3J_{CF}$ = 3.0 Hz), 129.5, 129.8, 137.4, 140.0 (d, $^3J_{CF}$ = 5.4 Hz), 166.8 ppm; (minor isomer) δ = 19.0 (d, $^3J_{CF}$ = 4.2 Hz), 25.1, 36.5, 40.0 (d, $^2J_{CF}$ = 21.5 Hz), 51.9 (d, $^2J_{CF}$ = 17.8 Hz), 52.2, 123.8 (d, $^1J_{CF}$ = 245.6 Hz), 128.5 (d, $^3J_{CF}$ = 2.6 Hz), 129.0, 129.5, 129.7, 135.3, 141.0 (d, $^3J_{CF}$ = 8.2 Hz), 166.8 ppm; ^{19}F NMR (376 MHz, $CDCl_3$): (major isomer) δ = -127.25 (d, $^3J_{HF}$ = 15.8 Hz) ppm; (minor isomer) δ = -116.42 (d, $^3J_{HF}$ = 13.6 Hz) ppm

(1*R,4*S**,5*S**,6*S**)-5-Fluoro-5-nitro-6-(4-nitrophenyl)bicyclo[2.2.2]oct-2-ene** (*exo*-**3c**; major isomer) and **(1*R**,4*S**,5*R**,6*R**)-5-fluoro-5-nitro-6-(4-nitrophenyl)bicyclo[2.2.2]oct-2-ene** (*endo*-**3c**; minor isomer). Eluent: Hex/DCM, 2:1; Hex/DCM, 1:1; 0.038 g, (26 %); *dr* = 37:63; viscous pale brown oil. Anal. calcd for $C_{14}H_{13}FN_2O_4$ (%): C, 57.53; H, 4.48; N, 9.59; Found : C, 57.81; H, 4.49; N, 9.42. ^{1}H NMR (400 MHz, $CDCl_3$): (major isomer) δ = 1.44 – 1.56 (m, 2H), 1.98 – 2.10 (m, 1H), 2.11 – 2.22 (m, 1H), 3.04 – 3.10 (m, 1H), 3.30 – 3.41 (m, 1H), 3.92 (d, $^3J_{HF}$ = 15.7 Hz, 1H), 6.28 – 6.34 (m, 1H), 6.67 (t, J = 7.3 Hz, 1H), 7.38 – 7.50 (m, 2H), 8.16 – 8.27 (m, 2H) ppm; (minor isomer) δ = 1.33 – 1.44 (m, 2H), 1.76 – 1.86 (m, 1H), 1.94 (td, J = 9.4, 2.8 Hz, 1H), 2.99 – 3.05 (m, 1H), 3.30 – 3.41 (m, 1H), 4.38 (d, J = 13.5 Hz, 1H), 6.34 – 6.40 (m, 1H), 6.75 (t, J = 7.2 Hz, 1H), 7.35 (d, J = 8.8 Hz, 2H), 8.10 – 8.17 (m, 2H) ppm; ^{13}C NMR (100 MHz, $CDCl_3$): (major isomer) δ = 17.2, 20.3 (d, $^3J_{CF}$ = 5.1 Hz), 35.7, 39.5 (d, $^2J_{CF}$ = 22.1 Hz), 49.5 (d, $^2J_{CF}$ = 18.6 Hz), 121.3 (d, $^1J_{CF}$ = 250.6 Hz), 123.7, 129.6 (d, $^3J_{CF}$ = 7.1 Hz), 130.5 (d, $^4J_{CF}$ = 3.0 Hz), 137.2, 142.2 (d, $^3J_{CF}$ = 5.4 Hz), 147.3 ppm; (minor isomer) δ = 18.9 (d, $^3J_{CF}$ = 4.2 Hz), 25.04, 36.6, 39.8 (d, $^2J_{CF}$ = 21.3 Hz), 51.6 (d, $^2J_{CF}$ = 17.6 Hz), 123.6 (d, $^1J_{CF}$ = 245.8 Hz), 123.6, 129.0 (d, $^3J_{CF}$ = 2.4 Hz), 130.0, 134.9, 143.3 (d, $^3J_{CF}$ = 8.2 Hz), 147.5 ppm; ^{19}F NMR (376 MHz, $CDCl_3$): (major isomer) δ = -127.03 (d, $^3J_{HF}$ = 15.7 Hz) ppm; (minor isomer) δ = -116.12 (d, $^3J_{HF}$ = 13.5 Hz) ppm.

Methyl 4-((1*R,2*R**,3*S**,4*R**)-3-fluoro-4-methoxy-3-nitrobicyclo[2.2.2]oct-5-en-2-yl)benzoate** (*exo*-**3d**-5-F; 24 %) and **methyl 4-((1*R**,2*S**,3*R**,4*R**)-3-fluoro-4-methoxy-3-nitrobicyclo[2.2.2]oct-5-en-2-yl)benzoate** (*endo*-**3d**-5-F; 20 %) and **(methyl 4-((1*S**,2*R**,3*S**,4*S**)-3-fluoro-1-methoxy-3-nitrobicyclo[2.2.2]oct-5-en-2-yl)benzoate** (*endo*-**3d**-6-F; 33 %) and **methyl 4-((1*S**,2*S**,3*R**,4*S**)-3-fluoro-1-methoxy-3-nitrobicyclo[2.2.2]oct-5-en-2-yl)benzoate** (*exo*-**3d**-6-F; 23 %). Eluent: Hex/DCM, 1:1; Hex/DCM, 2:3; Hex/DCM, 1:3; Hex/DCM, 1:5; Hex/DCM, 1:10; DCM. 0.067 g (40 %); isomers ratio = (24:20) : (23:33); yellowish oil; Anal. calcd for $C_{17}H_{18}FNO_5$ (%): C, 60.89; H, 5.41; N, 4.18; found: C, 60.85; H, 5.66; N, 4.08. ^{1}H NMR (400 MHz, $CDCl_3$): (*exo*-**3d**-5-F) δ = 1.57 – 1.68 (m, 2H), 2.22 – 2.32 (m, 2H), 2.94 – 3.01 (m, 1H), 3.51 (d, J = 1.3 Hz, 3H), 3.90 (s, 3H), 4.18 (d, $^3J_{HF}$ = 13.7 Hz, 1H), 6.50

(d, J = 8.6 Hz, 1H), 6.74 (dd, J = 8.6, 6.4 Hz, 1H), 7.13 (d, J = 8.4 Hz, 2H), 7.92 – 7.97 (m, 2H) ppm; (*endo*-**3d**-5-F) δ = 1.57 – 1.68 (m, 2H), 2.22 – 2.32 (m, 2H), 3.11 – 3.21 (m, 1H), 3.44 (s, 3H), 3.83 (d, $^3J_{HF}$ = 17.0 Hz, 1H), 3.91 (s, 3H), 6.37 – 6.44 (m, 1H), 6.61 (dd, J = 8.6, 6.7 Hz, 1H), 7.22 (d, J = 8.2 Hz, 2H), 7.98 – 8.02 (m, 2H) ppm; (*endo*-**3d**-6-F) δ = 1.51 – 1.74 (m, 2H), 2.07 – 2.34 (m, 2H), 3.25 (s, 3H), 3.26 – 3.34 (m, 1H), 3.91 (s, 3H), 4.13 (d, $^3J_{HF}$ = 15.0 Hz, 1H), 6.19 – 6.32 (m, 1H), 6.75 (d, J = 8.8 Hz, 1H), 7.37 (dd, J = 8.2, 1.4 Hz, 2H), 8.02 (d, J = 8.3 Hz, 2H) ppm; (*exo*-**3d**-6-F) δ = 1.51 – 1.74 (m, 2H), 2.07 – 2.34 (m, 2H), 3.18 (s, 3H), 3.26 – 3.34 (m, 1H), 3.89 (s, 3H), 4.56 (d, $^3J_{HF}$ = 12.7 Hz, 1H), 6.19 – 6.32 (m, 1H), 6.68 (d, J = 8.4 Hz, 1H), 7.27 (d, J = 5.2 Hz, 2H), 7.95 (d, J = 8.3 Hz, 2H) ppm; ^{13}C NMR (100 MHz, $CDCl_3$): δ = 18.7, 18.8, 20.5 (d, $^3J_{CF}$ = 3.4 Hz), 23.2, 23.8, 26.0, 27.4, 29.8, 34.1, 36.1, 39.0 (d, $^2J_{CF}$ = 22.2 Hz), 39.6 (d, $^2J_{CF}$ = 21.7 Hz), 41.2, 50.8, 51.8, 52.2, 52.3, 52.3, 53.1 (d, $^2J_{CF}$ = 18.5 Hz), 53.2 (d, $^2J_{CF}$ = 19.7 Hz), 53.6 (d, $^3J_{CF}$ = 2.1 Hz), 55.0 (d, $^2J_{CF}$ = 18.7 Hz), 55.4 (d, $^2J_{CF}$ = 17.7 Hz), 77.4, 79.0, 80.1, 81.3 (d, $^3J_{CF}$ = 4.3 Hz), 81.5, 120.8 (d, $^1J_{CF}$ = 250.1 Hz), 122.0 (d, $^1J_{CF}$ = 249.9 Hz), 123.1 (d, $^1J_{CF}$ = 246.4 Hz), 123.5 (d, $^1J_{CF}$ = 250.5 Hz), 126.5 (d, $^4J_{CF}$ = 1.2 Hz), 127.3 (d, $^3J_{CF}$ = 7.8 Hz), 128.1, 128.8 (d, $^4J_{CF}$ = 1.7 Hz), 128.8, 129.2 (d, $^4J_{CF}$ = 2.8 Hz), 129.4, 129.5, 129.7 (d, $^3J_{CF}$ = 10.9 Hz), 129.8, 129.9, 129.9, 130.0, 130.4, 130.5, 131.3 (d, J = 2.7 Hz), 133.2 (d, $^3J_{CF}$ = 4.6 Hz), 134.1 (d, $^3J_{CF}$ = 5.3 Hz), 137.0 (d, $^3J_{CF}$ = 5.6 Hz), 137.5 (d, $^3J_{CF}$ = 8.4 Hz), 137.9, 138.9, 139.4 (d, $^3J_{CF}$ = 5.8 Hz), 140.2 (d, $^3J_{CF}$ = 8.6 Hz), 166.7, 166.8, 166.9, 166.9 ppm. ^{19}F NMR (376 MHz, $CDCl_3$): (*exo*-**3d**-5-F) δ = -127.17 (dd, J = 13.7, 6.2 Hz) ppm; (*endo*-**3d**-5-F) δ = -136.93 (d, J = 17.0 Hz) ppm; (*endo*-**3d**-6-F) δ = -123.81 (d, J = 15.0 Hz) ppm; (*exo*-**3d**-6-F) δ = -114.71 – -114.50 (m) ppm.

General procedure for the epoxidation of cycloadducts 2. In a typical experiment, *m*-chloroperbenzoic acid (0.6 mmol) was added to solution of norbornene **2** (0.2 mmol) in DCM (1 mL). The reaction mixture was stirred overnight at room temperature. The reaction was monitored by TLC. After completion of the reaction the reaction mixture was poured into a saturated solution of $Na_2S_2O_3$ (10 mL) The resulting mixture was then extracted with DCM (3 × 15 mL). The combined organic layer was washed with saturated solution of $NaHCO_3$ (3 × 50 mL), dried over Na_2SO_4, filtered, and concentrated under vacuum. The pure product was isolated by column chromatography on neutral alumina using mixture of Hex/DCM in an appropriate ratio as the eluent.

(1*S,2*S**,4*R**,5*S**,6*S**,7*R**)-7-(4-Bromophenyl)-6-fluoro-6-nitro-3-oxatricyclo[3.2.1.02,4]octane** (*endo*-**4a**; major isomer) and **(1*S**,2*S**,4*R**,5*S**,6*R**,7*S**)-7-(4-bromophenyl)-6-fluoro-6-nitro-3-oxatricyclo[3.2.1.0^{2,4}]octane** (*exo*-**4a**; minor isomer). Eluent: Hex/DCM, 3:1; Hex/DCM, 2:1; 0.051 g, (81 %); *dr* = 41:59; yellowish viscous oil; HRMS (ESI-TOF) m/z: calcd for $C_{13}H_{12}{}^{80}BrFNO_3$ $[M+H]^+$ = 327.9985; found 327.9985. ^{1}H NMR (400 MHz, $CDCl_3$): (major isomer) δ = 1.73 – 1.78 (m, 2H), 3.08 – 3.19 (m, 1H), 3.29 (s, 1H), 3.47 – 3.52 (m, 1H), 3.58 (dd, J = 3.4, 0.8 Hz, 1H), 4.01 (dd, J = 11.2, 3.5 Hz, 1H), 7.13 (dd, J = 8.4, 1.4 Hz, 2H), 7.44 – 7.52 (m, 2H) ppm; (minor isomer) δ = 1.79 (br s, 1H), 1.85 – 1.92 (m, 1H), 3.00 (s, 1H), 3.08 –3.19 (m, 1H), 3.40 (d, J = 2.9 Hz, 1H), 3.54 (d, J = 2.9 Hz, 1H), 3.81 (dd,

J = 11.8, 2.7 Hz, 1H), 7.07 (d, J = 8.4 Hz, 2H), 7.44 –7.52 (m, 2H) ppm; ^{13}C NMR (100 MHz, $CDCl_3$): (major isomer) δ = 24.6 (d, $^3J_{CF}$ = 4.7 Hz), 41.6, 46.7 (d, $^3J_{CF}$ = 15.3 Hz), 47.9 (d, $^2J_{CF}$ = 16.8 Hz), 48.9, 54.3 (d, $^2J_{CF}$ = 18.5 Hz), 122.2, 123.2 (d, $^1J_{CF}$ = 255.4 Hz), 131.0 (d, $^4J_{CF}$ = 2.7 Hz), 131.4 (d, $^3J_{CF}$ = 2.8 Hz), 131.9 ppm; (minor isomer) δ = 25.2, 41.8, 47.0 (d, $^3J_{CF}$ = 8.6 Hz), 47.4 (d, $^2J_{CF}$ = 22.0 Hz), 50.3, 51.8 (d, $^2J_{CF}$ = 19.0 Hz), 122.3, 124.7 (d, $^1J_{CF}$ = 253.2 Hz), 130.3, 132.0, 132.6 (d, $^3J_{CF}$ = 7.5 Hz) ppm; ^{19}F NMR (376 MHz, $CDCl_3$): (major isomer) δ = -134.48 (d, $^3J_{HF}$ = 11.2 Hz) ppm, (minor isomer) δ = -117.21 (dd, J = 11.8, 7.3 Hz) ppm.

(1*S,2*S**,4*R**,5*S**,6*S**,7*R**)-6-Fluoro-6-nitro-7-(4-nitrophenyl)-3-oxatricyclo[3.2.1.0^{2,4}]octane** (*endo*-**4b**; major isomer) and **(1*S**,2*S**,4*R**,5*S**,6*R**,7*S**)-6-fluoro-6-nitro-7-(4-nitrophenyl)-3-oxatricyclo[3.2.1.02,4]octane** (*exo*-**4b**; minor isomer). Eluent: Hex/DCM, 2:1; Hex/DCM, 1:1; Hex/DCM, 1:2; 0.048 g, (85 %); *dr* = 33:67; pale brown viscous oil; HRMS (ESI-TOF) m/z: calcd for $C_{13}H_{12}FN_2O_5$ $[M+H]^+$ = 295.0730; found 295.0723; ^{1}H NMR (400 MHz, $CDCl_3$): (major isomer) δ = 1.71 – 1.87 (m, 2H), 3.25 (br s, 1H), 3.35 (br s, 1H), 3.50 – 3.54 (m, 1H), 3.57 (d, J = 3.3 Hz, 1H), 4.16 (dd, J = 11.3, 3.5 Hz, 1H), 7.43 – 7.50 (m, 2H), 8.16 – 8.25 (m, 2H) ppm; (minor isomer) δ = 1.71 – 1.87 (m, 1H), 1.90 – 1.98 (m, 1H), 3.09 (d, J = 1.0 Hz, 1H), 3.18 (d, J = 6.9 Hz, 1H), 3.41 (d, J = 3.2 Hz, 1H), 3.57 (d, J = 3.2 Hz, 1H), 3.97 (dd, J = 11.8, 2.8 Hz, 1H), 7.40 (d, J = 8.7 Hz, 2H), 8.25 – 8.16 (m, 2H) ppm; ^{13}C NMR (100 MHz, $CDCl_3$): (major isomer) δ = 24.6 (d, $^3J_{CF}$ = 4.8 Hz), 41.6, 46.6 (d, $^3J_{CF}$ = 15.4 Hz), 47.7 (d, $^2J_{CF}$ = 16.7 Hz), 54.3 (d, $^2J_{CF}$ = 18.3 Hz), 48.6, 123.0 (d, $^1J_{CF}$ = 255.8 Hz), 123.8, 130.3 (d, $^4J_{CF}$ = 2.9 Hz), 139.8 (d, $^3J_{CF}$ = 2.4 Hz), 147.5 ppm; (minor isomer) δ = 25.1, 41.7, 46.8 (d, $^3J_{CF}$ = 8.6 Hz), 47.3 (d, $^2J_{CF}$ = 21.9 Hz), 50.1, 51.8 (d, $^2J_{CF}$ = 18.7 Hz), 124.0, 124.7 (d, $^1J_{CF}$ = 253.5 Hz), 129.7, 140.9 (d, $^3J_{CF}$ = 7.6 Hz), 147.7 ppm; ^{19}F NMR (376 MHz, $CDCl_3$): (major isomer) δ = -134.34 (d, $^3J_{HF}$ = 11.3 Hz) ppm; (minor isomer) δ = -117.10 (dd, J = 11.8, 7.3) ppm.

(1*S,2*S**,4*R**,5*S**,6*S**,7*R**)-6-Fluoro-6-nitro-7-(*p*-tolyl)-3-oxatricyclo[3.2.1.0^{2,4}]octane** (*endo*-**4c**; major isomer) and **(1*S**,2*S**,4*R**,5*S**,6*R**,7*S**)-6-fluoro-6-nitro-7-(*p*-tolyl)-3-oxatri-cyclo[3.2.1.02,4]octane** (*exo*-**4c**; minor isomer). Eluent: Hex/DCM, 3:1; Hex/DCM, 2:1; 0.051 g, (87 %); *dr* = 45:55; pale yellow viscous oil; HRMS (ESI-TOF) m/z: calcd for $C_{14}H_{15}FNO_3$ $[M+H]^+$ = 264.1036; found 264.1032. ^{1}H NMR (400 MHz, $CDCl_3$): (major isomer) δ = 1.77 (s, 1H), 1.79 –1.92 (m, 1H), 2.35 (s, 3H), 3.15 (br s, 1H), 3.27 (s, 1H), 3.51 (dt, J = 3.4, 1.6 Hz, 1H), 3.66 (dd, J = 3.4, 1.1 Hz, 1H), 4.04 (dd, J = 11.7, 3.4 Hz, 1H), 7.09 (d, J = 8.1 Hz, 1H), 7.11 – 7.21 (m, 3H) ppm; (minor isomer) δ = 1.77 (s, 1H), 1.79 –1.92 (m, 1H), 2.35 (s, 3H), 3.02 (s, 1H), 3.10 (d, J = 6.6 Hz, 1H), 3.41 (d, J = 2.6 Hz, 1H), 3.54 (dd, J = 3.3, 1.1 Hz, 1H), 3.83 (dd, J = 11.9, 2.4 Hz, 1H), 7.09 (d, J = 8.1 Hz, 1H), 7.11 – 7.21 (m, 3H) ppm; ^{13}C NMR (100 MHz, $CDCl_3$): (major isomer) δ = 21.1, 24.6 (d, $^3J_{CF}$ = 4.6 Hz), 41.7, 46.9 (d, $^3J_{CF}$ = 15.5 Hz), 48.0 (d, $^2J_{CF}$ = 16.8 Hz), 49.3, 54.7 (d, $^2J_{CF}$ = 18.7 Hz), 123.4 (d, $^1J_{CF}$ = 255.2 Hz), 129.3 (d, $^4J_{CF}$ = 2.4 Hz), 129.4 (d, $^3J_{CF}$ = 2.9 Hz), 129.4, 137.8 ppm; (minor isomer) δ = 21.2, 25.3, 41.9, 47.2 (d, $^3J_{CF}$ = 8.5 Hz), 47.5 (d, $^2J_{CF}$ = 22.2 Hz), 50.5, 52.2 (d, $^2J_{CF}$ = 19.2 Hz), 124.9 (d, $^1J_{CF}$ = 253.0 Hz), 128.5, 129.6, 130.5

(d, $^3J_{CF}$ = 7.4 Hz), 138.0 ppm; ^{19}F NMR (376 MHz, $CDCl_3$): (major isomer) δ = -134.50 (d, *J* = 11.7 Hz) ppm; (minor isomer): δ = -117.45 (dd, *J* = 11.9, 7.3 Hz) ppm.

Dihydroxylation of the cycloadduct 2f. *N*-Methylmorpholine *N*-oxide (0.031 g, 1.5 mol equiv) was added to a solution of **2f** (0.056 g, 1 mol equiv) in 1.2 mL of acetone/water 3:1. Then, 100 μL of OsO_4 1% solution (0.001 g, 2 mol %) was added. Then reaction mixture was stirred for 5 h at room temperature. After reaction completion (TLC monitoring), the reaction mixture was concentrated under vacuum, poured in 20 mL of water and then extracted with EtOAc (3 × 20 mL). The combined organic extract was dried over Na_2SO_4, filtrated, and concentrated under vacuum. The pure product was isolated by column chromatography on silica using mixture of Hex/EtOAc as the eluent.

(1*S,2*S**,3*R**,4*S**,5*S**,6*R**)-6-(4-Bromophenyl)-5-fluoro-5-nitrobicyclo[2.2.1]heptane-2,3-diol** (*endo*-**5,** major isomer) and **(1*S**,2*S**,3*R**,4*S**,5*R**,6*S**)-6-(4-bromophenyl)-5-fluoro-5-nitrobi-cyclo[2.2.1]heptane-2,3-diol** (*exo*-**5,** minor isomer). Eluent: Hex/EtOAc 1:1, Hex/EtOAc 1:2; 0.040 g (65 %); colorless solid. HRMS (ESI) m/z: calcd for $C_{13}H_{13}{}^{107}Ag^{81}BrFNO_4$ $[M+Ag]^+$ = 453.9042; found 453.9042; ^{1}H NMR (400 MHz, $CDCl_3$): (major isomer) δ = 2.15 (d, *J* = 11.7 Hz, 1H), 2.26 (dd, *J* = 11.8, 1.5 Hz, 1H), 2.85 (s, 1H), 2.92 (s, 1H), 3.05 – 3.29 (m, 2H), 3.96 (dd, *J* = 12.7, 4.1 Hz, 1H), 4.22 (s, 1H), 4.26 (s, 1H), 7.07 (d, *J* = 7.7 Hz, 2H), 7.47 (d, *J* = 7.7 Hz, 2H) ppm; (minor isomer) δ = 2.20 (d, *J* = 11.7 Hz, 1H), 2.34 (d, *J* = 11.7 Hz, 1H), 2.67 (s, 1H), 2.74 (d, *J* = 7.4 Hz, 1H), 3.29 – 3.47 (m, 2H), 3.78 (d, *J* = 13.1 Hz, 1H), 4.11 (s, 1H), 4.18 (s, 1H), 7.02 (d, *J* = 8.4 Hz, 2H), 7.45 (d, *J* = 8.4 Hz, 2H) ppm. ^{13}C NMR (100 MHz, $CDCl_3$): (major isomer) δ = 31.7 (d, $^3J_{CF}$ = 3.1 Hz), 47.1, 52.4 (d, $^2J_{CF}$ = 19.6 Hz), 54.7 (d, $^2J_{CF}$ = 17.2 Hz), 67.8 (d, $^3J_{CF}$ = 14.6 Hz), 68.2, 121.7 (d, $^1J_{CF}$ = 255.6 Hz), 121.8, 130.5 (d, $^4J_{CF}$ = 2.3 Hz), 131.2 (d, $^3J_{CF}$ =2.9 Hz), 131.9 ppm; (minor isomer) δ = 32.2, 48.2, 50.7 (d, $^2J_{CF}$ = 19.0 Hz), 53.2 (d, $^2J_{CF}$ = 21.3 Hz), 67.5 (d, $^3J_{CF}$ = 8.3 Hz), 73.0, 122.1, 123.2 (d, $^1J_{CF}$ = 251.2 Hz), 130.0, 132.0, 132.6 (d, $^3J_{CF}$ = 7.8 Hz) ppm. ^{19}F NMR (376 MHz, $CDCl_3$): (major isomer) δ = - 137.76 (s) ppm; (minor isomer) δ =-124.12 (s) ppm.

Base-induced nitrous acid elimination from cycloadduct 3l. Cycloadduct **2l** (0.052 g, 1 mol equiv) was dissolved in THF (1 mL) and the solution was loaded into a vial covered with aluminum foil. Then, potassium *tert*-butoxide (2 mol equiv) was added in the darkness in several portions over 20 min to the vigorously stirred reaction mixture. The reaction mixture was stirred overnight at room temperature and the reaction was monitored by TLC. After completion of the reaction the reaction mixture was passed through a column charged with alumina and covered with an aluminum foil using DCM as eluent. The solution of the pure product **6** was collected in a flask covered with aluminum foil and then concentrated under vacuum.

2-Fluoro-3-(4-nitrophenyl)bicyclo[2.2.1]hepta-2,5-diene (**6**). 0.034 g (77 %); yellow oil; ^{1}H NMR (400 MHz, $CDCl_3$) δ = 2.28 (ddd, *J* = 6.5, 4.7, 1.9 Hz, 1H), 2.41 – 2.46 (m, 1H), 3.47 – 3.53 (m, 1H), 3.88 – 3.94 (m, 1H), 6.95 (dd, *J* = 4.5, 3.1 Hz, 1H), 7.05 (dt, *J* = 4.6, 2.2 Hz, 1H), 7.47 – 7.56 (m, 2H), 8.16 – 8.20

(m, 2H) ppm; ^{19}F NMR (376 MHz, $CDCl_3$): δ = -110.34 (s) ppm. The analysis of the sample was consistent with the previously reported data [4].

Kinetic studies

Experimental procedure. Analogous as described in [3], in a typical kinetic experiment, nitrostyrene **1h** (0.025 mmol; 1 mol equiv), *o*-xylene (0.1 mL), and 1,3-cyclopentadiene (0.1 mL; 49 mol equiv) or 1,3-cyclohexadiene (0.1 mL; 43 mol equiv) were successively loaded into a series of screw-top vials filled with argon. After the caps were screwed tightly, the reaction mixtures were heated in a thermostat at preset temperature (50–130 °C) with vigorous stirring for the set time. After the heating completion, the reaction mixtures were cooled, diluted with DCM, passed through a silica pad, and concentrated under vacuum. The resulting mixtures were analyzed by ^{1}H NMR spectroscopy. The ratio of isomers and conversions (*F*) were calculated from the integral values of characteristic peaks of the starting nitrostyrene **1h** and the products.

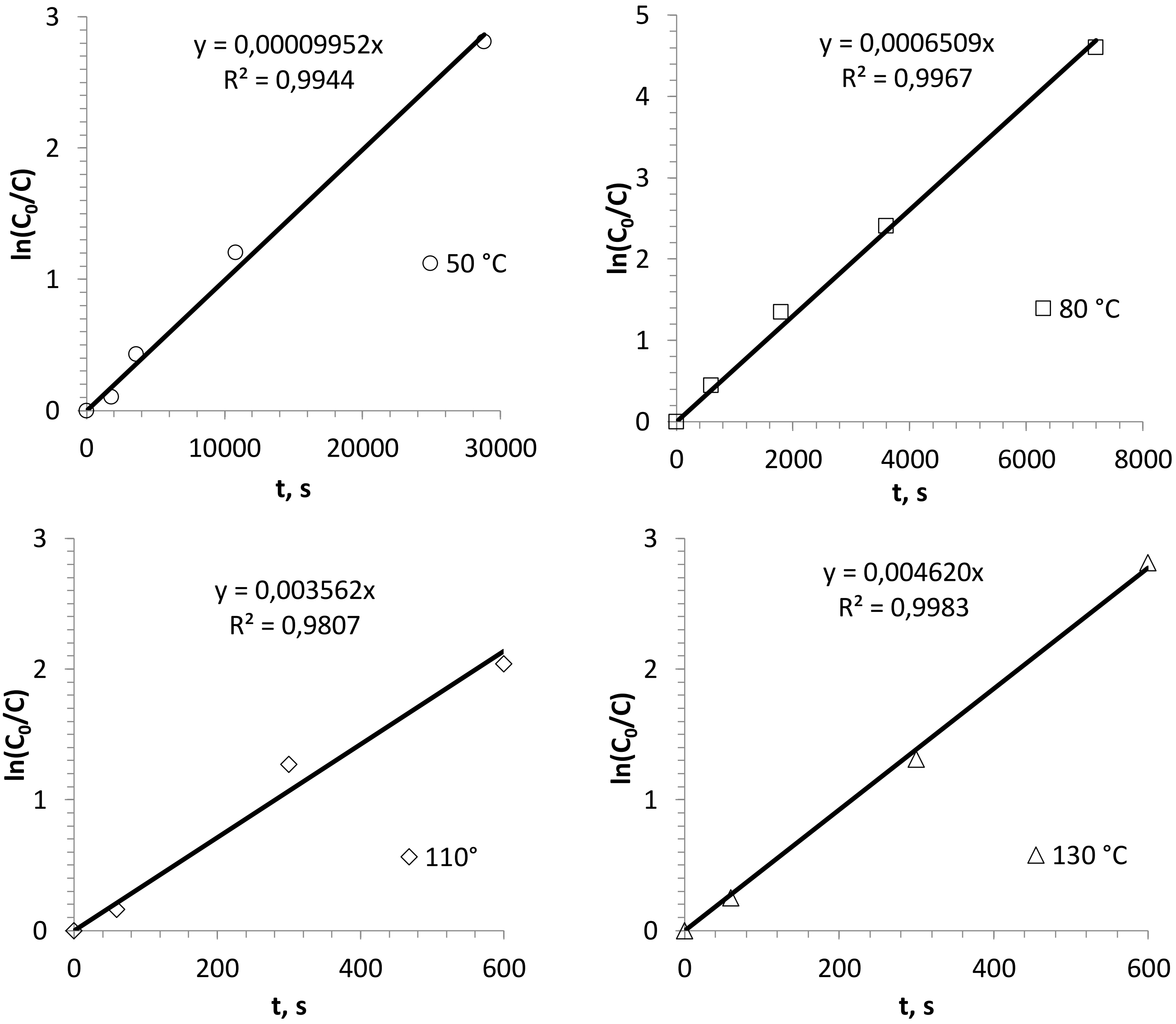

Figure S1: Plot of ln(C_0/C) *versus t* for the reactions of nitrostyrene **1h** with 1,3-cyclopentadiene at 50–130 °C.

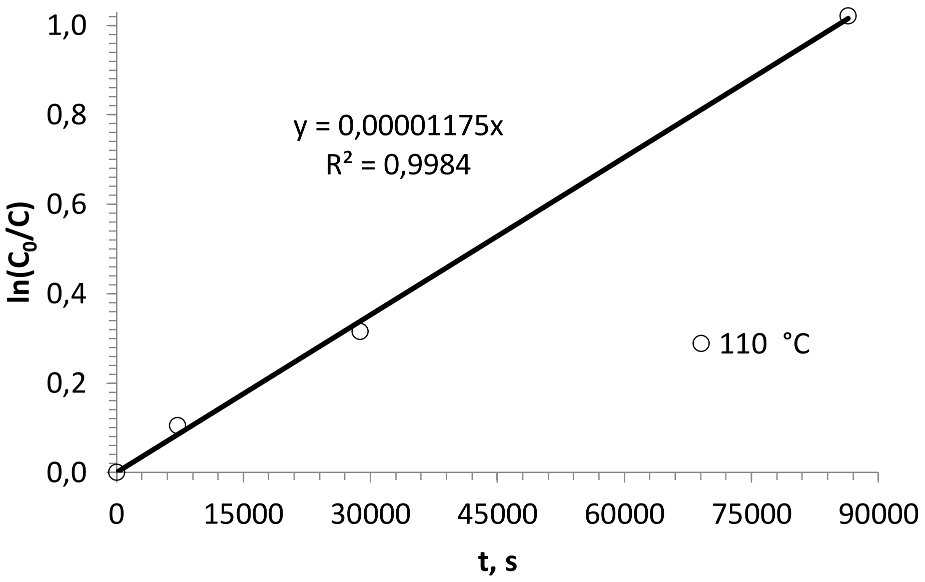

Figure S2: Plot of ln(C_0/C) *versus* t for the reaction of nitrostyrene **1h** with 1,3-cyclohexadiene at 110 °C.

3. Computational details

DFT calculations were performed using Gaussian 16, Rev. A.03[5]. The B3LYP [6,7,8] and M062X [9] functionals were used in conjunction with the 6-311+G(d,p) Pople basis set [10,11] and IEFPCM [12] solvation for *o*-xylene. The calculated ground states (GS) and transition states (TS) were confirmed by vibrational frequency analysis (GS: $N_{\text{imag}} = 0$, TS: $N_{\text{imag}} = 1$). Initial guesses for transition states were based on relaxed bond-length scan calculations (M062X/6-31G [13,14]) of the newly formed σ-bonds in the Diels–Alder reaction. Furthermore, IRC calculations confirmed that the transition states lead to the corresponding products and starting materials. Visual assessment of the vibration belonging to the imaginary frequency of the transition state using GaussView 6 [15] provided further confirmation of the transition state. In contrast to the geometry calculations, the frequency calculations for the thermochemical relevant energies were calculated at 383.15K with the corresponding optimized geometry.

Input Lines

Geometry optimization of a ground state:

#p opt=tight freq=noraman b3lyp/6-311+g(d,p) scrf=(iefpcm,solvent=o-xylene)

#p opt=tight freq=noraman m062x/6-311+g(d,p) scrf=(iefpcm,solvent=o-xylene)

Scan calculation (7 steps á 0.2 Å):

#p opt=modredundant m062x/6-31g scrf=(iefpcm,solvent=o-xylene)

Geometry optimization of a transition state:

#p opt=(calcfc,tight,ts,noeigen) freq=noraman scf=qc b3lyp/6-311+g(d,p) scrf=(iefpcm,solvent=o-xylene)

#p opt=(calcfc,tight,ts,noeigen) freq=noraman scf=qc m062x/6-311+g(d,p) scrf=(iefpcm,solvent=o-xylene)

IRC calculation of a transition state:

#p irc=(calcall,maxcyc=100) b3lyp/6-311+g(d,p) scrf=(iefpcm,solvent=o-xylene)

#p irc=(calcall,maxcyc=100) m062x/6-311+g(d,p) scrf=(iefpcm,solvent=o-xylene)

Frequency calucations for the thermochemical relevant energies:

#p freq=noraman b3lyp/6-311+g(d,p) scrf=(iefpcm,solvent=o-xylene) temperature=383.15

#p freq=noraman m062x/6-311+g(d,p) scrf=(iefpcm,solvent=o-xylene) temperature=383.15

Number of imaginary frequencies, enthalpies, electronic- and Gibbs energies calculated with B3LYP/6-311G+(d,p) and M062X/6-311G+(d,p) in *o*-xylene at 383.15K

(Colorcode: light grey: hydrogen, grey: carbon, red: oxygen, light blue: fluorine, blue: nitrogen)

Cyclopentadiene

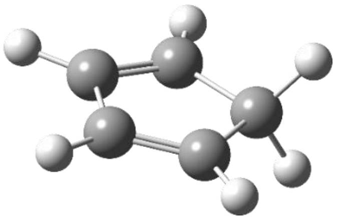

B3LYP

SCF Done: E(RB3LYP) = -194.157367733

Sum of electronic and thermal Enthalpies = -194.060247

Sum of electronic and thermal Enthalpies (383.15 K)= -194.057478

Sum of electronic and thermal Free Energies: = -194.091953

Sum of electronic and thermal Free Energies (383.15 K)= -194.101341

Number of imaginary frequencies = 0

M062X

SCF Done: E(RM062X) = -194.060330837

Sum of electronic and thermal Enthalpies = -193.962200

Sum of electronic and thermal Enthalpies (383.15 K)= -193.959451

Sum of electronic and thermal Free Energies = -193.993889

Sum of electronic and thermal Free Energies (383.15 K)= -194.003265

Number of imaginary frequencies = 0

β-Fluoro-β-nitrostyrene **1h**

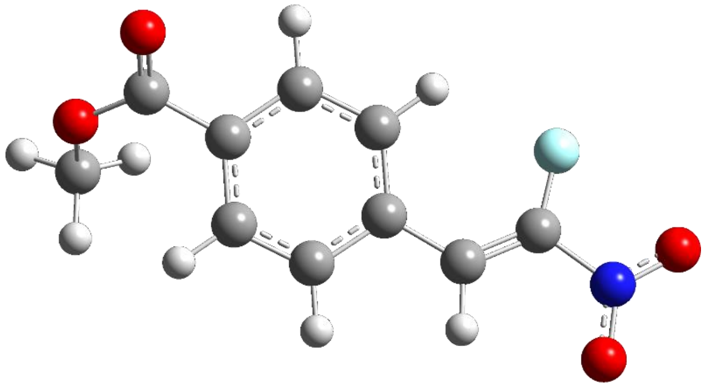

B3LYP

SCF Done: E(RB3LYP) = -841.490693409

Sum of electronic and thermal Enthalpies = -841.305171

Sum of electronic and thermal Enthalpies (383.15 K)= -841.297053

Sum of electronic and thermal Free Energies: = -841.365128

Sum of electronic and thermal Free Energies (383.15 K)= -841.383244

Number of imaginary frequencies = 0

M062X

SCF Done: E(RM062X) = -841.157231614

Sum of electronic and thermal Enthalpies = -840.968876

Sum of electronic and thermal Enthalpies (383.15 K)= -840.960875

Sum of electronic and thermal Free Energies = -841.028147

Sum of electronic and thermal Free Energies (383.15 K)= -841.046052

Number of imaginary frequencies = 0

endo-**TS**

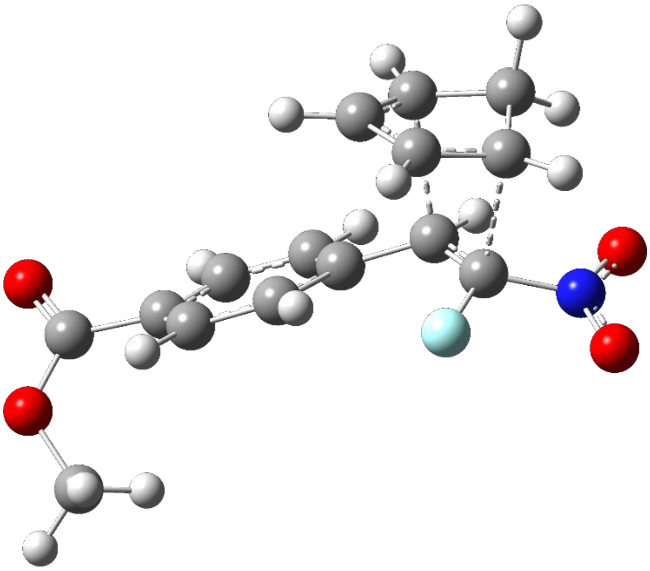

B3LYP

SCF Done: E(RB3LYP) = -1035.61479970

Sum of electronic and thermal Enthalpies = -1035.330728

Sum of electronic and thermal Enthalpies (383.15 K)= -1035.319748

Sum of electronic and thermal Free Energies: = -1035.399147

Sum of electronic and thermal Free Energies (383.15 K)= -1035.420031

Number of imaginary frequencies = 1

M062X

SCF Done: E(RM062X) = -1035.20424511

Sum of electronic and thermal Enthalpies = -1034.916372

Sum of electronic and thermal Enthalpies (383.15 K)= -1034.905558

Sum of electronic and thermal Free Energies = -1034.983311

Sum of electronic and thermal Free Energies (383.15 K)= -1035.003750

Number of imaginary frequencies = 1

exo-**TS**

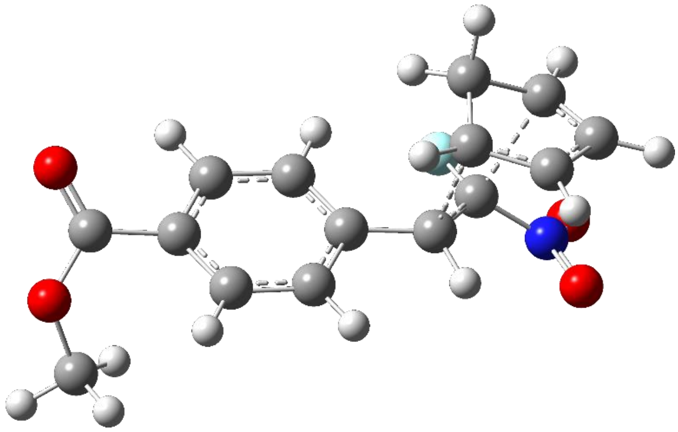

B3LYP

SCF Done: E(RB3LYP) = -1035.61454155

Sum of electronic and thermal Enthalpies = -1035.330383

Sum of electronic and thermal Enthalpies (383.15 K)= -1035.319400

Sum of electronic and thermal Free Energies: = -1035.398579

Sum of electronic and thermal Free Energies (383.15 K)= -1035.419401

Number of imaginary frequencies = 1

M062X

SCF Done: E(RM062X) = -1035.20415586

Sum of electronic and thermal Enthalpies = -1034.916118

Sum of electronic and thermal Enthalpies (383.15 K)= -1035.003376

Sum of electronic and thermal Free Energies = -1034.982962

Sum of electronic and thermal Free Energies (383.15 K)= -1034.905309

Number of imaginary frequencies = 1

Norbornene *endo*-**2h**

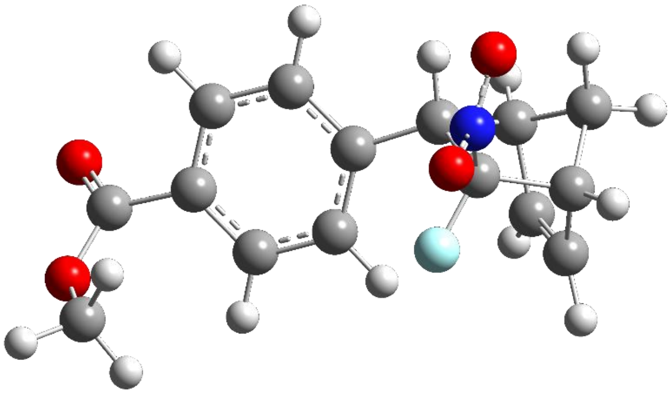

B3LYP

SCF Done: E(RB3LYP) = -1035.669464660

Sum of electronic and thermal Enthalpies = -1035.381415

Sum of electronic and thermal Enthalpies (383.15 K)= -1035.370605

Sum of electronic and thermal Free Energies: = -1035.447906

Sum of electronic and thermal Free Energies (383.15 K)= -1035.468215

Number of imaginary frequencies = 0

M062X

SCF Done: E(RM062X) = -1035.271688850

Sum of electronic and thermal Enthalpies = -1034.979554

Sum of electronic and thermal Enthalpies (383.15 K)= -1034.968939

Sum of electronic and thermal Free Energies = -1035.045276

Sum of electronic and thermal Free Energies (383.15 K)= -1035.065341

Number of imaginary frequencies = 0

Norbornene *exo*-**2h**

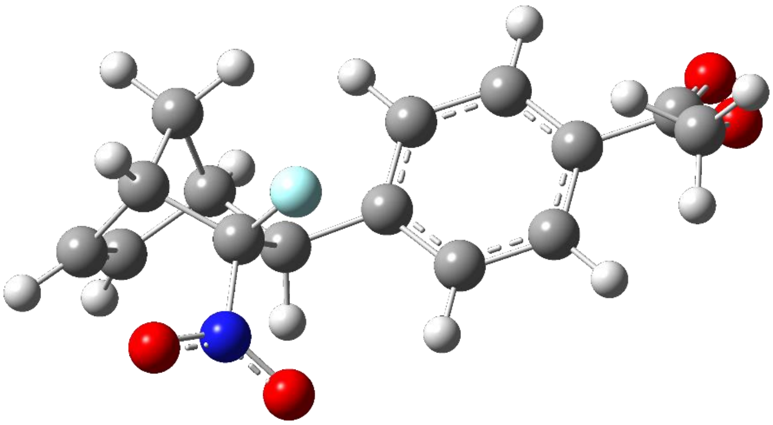

B3LYP

SCF Done: E(RB3LYP) = -1035.668394740

Sum of electronic and thermal Enthalpies = -1035.380504

Sum of electronic and thermal Enthalpies (383.15 K)= -1035.369679

Sum of electronic and thermal Free Energies: = -1035.447762

Sum of electronic and thermal Free Energies (383.15 K)= -1035.468292

Number of imaginary frequencies = 0

M062X

SCF Done: E(RM062X) = -1035.271008860

Sum of electronic and thermal Enthalpies = -1034.978820

Sum of electronic and thermal Enthalpies (383.15 K)= -1034.968197

Sum of electronic and thermal Free Energies = -1035.044396

Sum of electronic and thermal Free Energies (383.15 K)= -1035.064422

Number of imaginary frequencies = 0

References

[1] Williams, D. B. G.; Lawton, M. *J. Org. Chem.* **2010**, *75*, 8351–8354
[2] Motornov, V. A.; Muzalevskiy, V. M.; Tabolin, A. A.; Novikov, R. A.; Nelyubina, Yu. V.; Nenajdenko, V.G.; Ioffe, S. L. *J. Org. Chem.* **2017**, *82*, 5274–5284
[3] Larkovich, R. V.; Ponomarev, S. A.; Aldoshin, A. S.; Tabolin, A. A.; Ioffe, S. L.; Nenajdenko, V.G. *Eur. J. Org. Chem.* **2020**, 2479–2492
[4] Shastin, A. V.; Nenajdenko, V. G.; Muzalevskiy, V. M.; Balenkova, E. S.; Fröhlich, R.; Haufe, G. *Tetrahedron* **2008**, *64*, 9725- 9732
[5] Frisch, M. J.; Trucks, G. W.; Schlegel, H. B.; Scuseria, G. E.; Robb, M. A.; Cheeseman, J. R.; Scalmani, G.; Barone, V.; Petersson, G. A.; Nakatsuji, H.; Li, X.; Caricato, M.; Marenich, A. V.; Bloino, J.; Janesko, B. G.; Gomperts, R.; Mennucci, B.; Hratchian, H. P.; Ortiz, J. V.; Izmaylov, A. F.; Sonnenberg, J. L.; Williams; Ding, F.; Lipparini, F.; Egidi, F; Goings, J.; Peng, B.; Petrone, A.; Henderson, T.; Ranasinghe, D.; Zakrzewski, V. G.; Gao, J.; Rega, N.; Zheng, G.; Liang, W.; Hada, M.; Ehara, M.; Toyota, K.; Fukuda, R.; Hasegawa, J.; Ishida, M.; Nakajima, T.; Honda, Y.; Kitao, O.; Nakai, H.; Vreven, T.; Throssell, K.; Montgomery Jr., J. A.; Peralta, J. E.; Ogliaro, F.; Bearpark, M. J.; Heyd, J. J.; Brothers, E. N.; Kudin, K. N.; Staroverov, V. N.; Keith, T. A.; Kobayashi, R.; Normand, J.; Raghavachari, K.; Rendell, A. P.; Burant, J. C.; Iyengar, S. S.; Tomasi, J.; Cossi, M.; Millam, J. M.; Klene, M.; Adamo, C.; Cammi, R.; Ochterski, J. W.; Martin, R. L.; Morokuma, K.; Farkas, O.; Foresman, J. B.; Fox, D. J.Wallingford, CT, **2016**
[6] Lee, C.; Yang, W.; Parr, R. G. *Phys. Rev. B* **1988**, *37*, 785-789
[7] Becke, A. D. *J. Chem. Phys.* **1993**, *98*, 5648-5652
[8] Vosko, S. H.; Wilk, L.; Nusair, M. *Can. J. Phys.* **1980**, *58*, 1200-1211
[9] Zhao, Y.; Truhlar, D. G. *Theor. Chem. Acc.* **2008**, *120*, 215-241
[10] Krishnan, R.; Binkley, J. S.; Seeger, R.; Pople, J. A. *J. Chem. Phys.* **1980**, *72*, 650-654
[11] Frisch, M. J.; Pople, J. A.; Binkley, J. S. *J. Chem. Phys.* **1984**, *80*, 3265-3269
[12] Tomasi, J.; Mennucci, B.; Cancès, E. *J. Mol. Struct.* **1999**, *464*, 211-226
[13] Hehre, W. J.; Ditchfield, R.; Pople, J. A. *J. Chem. Phys.* **1972**, *56*, 2257-2261
[14] Hariharan, P. C.; Pople, J. A. *Theor. Chim. Acta* **1973**, *28*, 213-222
[15] Dennington, R.; Keith, T. A.; Millam, J. M. Semichem Inc. Shawnee Mission KS, **2016**
